# The global burden of otitis media in 204 countries and territories from 1992 to 2021: a systematic analysis for the Global Burden of Disease study 2021

**DOI:** 10.3389/fpubh.2024.1519623

**Published:** 2025-01-21

**Authors:** Guan-Jiang Huang, Bao-Rui Lin, Pei-Shan Li, Na Tang, Zhi-Jun Fan, Biao-Qing Lu

**Affiliations:** Department of Otorhinolaryngology Head and Neck Surgery, Zhongshan Hospital of Traditional Chinese Medicine, Affiliated to Guangzhou University of Chinese Medicine, Zhongshan, China

**Keywords:** otitis media, Global Burden of Disease (GBD), incidence, prevalence, prediction

## Abstract

**Objectives:**

This study aims to analyze the global burden of otitis media and predict future trends using data from the Global Burden of Disease 2021 (GBD 2021).

**Study design:**

A cross-sectional analysis of GBD 2021 results was conducted.

**Methods:**

Age-standardized incidence rates (ASIR), age-standardized prevalence rates (ASPR), and age-standardized disability-adjusted life years (DALYs) rates (ASDR) were calculated. Trend analysis was conducted using estimated annual percentage change (EAPC), Joinpoint regression, age-period-cohort, and decomposition analyses. Future projections were generated using Bayesian age-period-cohort (BAPC) and auto-regressive integrated moving average (ARIMA) models.

**Results:**

The global incidence of otitis media rose from 322.1 million cases in 1992 to 391.3 million in 2021, with ASIR increasing slightly from 5345.09 to 5529.1 per 100,000 (EAPC: 0.11%). Despite this increase, the ASPR decreased from 1786.56 to 1593.74 (EAPC: -0.43%). DALYs increased from 2.16 million to 2.48 million; however, ASDR declined from 37.68 to 32.54 per 100,000 (EAPC: −0.51%). Notably, low and low-middle SDI regions showed significant disparities, with higher ASIRs (up to 5315.08 for males) but declining trends in ASPR. Regionally, East Asia exhibited the most substantial decline in ASPR (−1.14%) and ASDR (−1.22%), while Central Sub-Saharan Africa demonstrated stable ASDR. Future projections indicate a rising ASIR and declining ASPR and ASDR through 2036.

**Conclusion:**

The global burden of otitis media shows significant regional disparities, with stable incidence but declining prevalence and DALYs rate. Public health interventions have been effective in higher SDI regions, but targeted efforts are needed in low and low-middle SDI regions to further reduce the burden of otitis media.

## Introduction

Otitis media, a group of inflammatory diseases of the middle ear, remains one of the most common health concerns affecting children and adults worldwide (1–4). It is a significant global health concern, particularly affecting children and leading to severe complications like hearing loss and intracranial infections (1, 2, 5). Despite advances in medical treatment and public health initiatives, the global burden of otitis media continues to challenge healthcare systems, particularly in low and middle-income countries, where access to healthcare is limited (5). The disease is responsible for significant morbidity, particularly among children under 5 years, contributing to long-term consequences like speech and language development delays, poor academic performance, and diminished quality of life (6, 7). Understanding the trends in the incidence, prevalence, and burden of otitis media is crucial for developing targeted interventions and policies that can alleviate its global impact (4, 5).

The incidence, prevalence, and burden of otitis media are influenced by a myriad of factors, including socioeconomic status, access to healthcare, vaccination coverage, and environmental conditions (5, 8). In high-income countries, widespread use of vaccines against common pathogens like *Streptococcus pneumoniae* and *Haemophilus influenzae* has led to a decline in the incidence of acute otitis media (9, 10). However, in low and middle-income countries, where vaccination coverage may be inadequate, otitis media remains a pervasive issue. Furthermore, the condition is often exacerbated by factors such as overcrowding, poor nutrition, and limited access to clean water and sanitation (10–12). These disparities underscore the need for a nuanced understanding of the global epidemiology of otitis media, which can inform the allocation of resources and the implementation of effective public health strategies (5, 12).

The Socio-Demographic Index (SDI), a composite measure that considers income per capita, education level, and fertility rates, has been shown to correlate with the burden of otitis media (13, 14). High SDI regions generally report lower incidence and prevalence rates, likely due to better access to healthcare, higher vaccination coverage, and improved living conditions (9, 15). Conversely, low and middle SDI regions continue to struggle with high rates of otitis media, reflecting broader inequities in healthcare and social determinants of health (12, 14).

Given the persistent and widespread nature of otitis media, this study provides a comprehensive analysis of global trends in the incidence, prevalence, and disability-adjusted life years (DALYs) of otitis media from 1992 to 2021 and projections for future trends up to 2036, aiming to offer insights into areas where targeted interventions may be most effective.

## Materials and methods

### Data source

This study utilized the latest released data from the Global Burden of Disease (GBD) database,^1^ spanning from 1992 to 2021, to analyze global trends in otitis media. The GBD database, maintained by the Institute for Health Metrics and Evaluation (IHME), is a comprehensive resource that provides estimates of incidence, prevalence, mortality, and DALYs across a wide range of diseases (16, 17). The data extracted for this study included age-standardized incidence rates (ASIR), age-standardized prevalence rates (ASPR), and age-standardized DALY rates (ASDR), all standardized to a global age structure to facilitate comparisons across regions and time periods (16, 17).

### Statistical analysis

Trend analysis contained estimated annual percentage change (EAPC), Joinpoint regression, age-period-cohort (APC), and decomposition analyses. EAPC was calculated using a regression model where the natural logarithm of the rates was regressed against the calendar year (18). Joinpoint regression analysis was employed to detect significant changes on percentage change (APC) and average annual percentage change (AAPC) in trends over time for ASIR, ASPR, and ASDR (19). An age-period-cohort (APC) analysis was conducted to decompose the observed trends into age, period, and cohort effects (20). This analysis was essential for understanding how different factors contributed to the observed changes in otitis media burden over time. Decomposition analysis was performed to attribute changes in otitis media burden to specific factors such as population growth, aging, and changes in age-specific rates (21). Health inequality analysis of incidence, prevalence, and DALY rates were also conducted to assess the relationship with SDI in different countries or regions. Future trends were projected using Bayesian age-period-cohort (BAPC) and auto-regressive integrated moving average (ARIMA) models (20–22).

Except for the Joinpoint regression analysis, other statistical analyses were conducted using R software (version 4.4.1), with specific packages (such as “Joinpoint,” “Epi,” “BAPC,” “INLA,” etc.) utilized for the respective analyses. *p* < 0.05 suggests statistically significant. Joinpoint regression analysis was conducted using the Joinpoint software (version 5.3.0).

### Data visualization

Global distribution maps for ASIR, ASPR, and ASDR displayed the distribution of otitis media burden across different countries and regions, with color gradients representing the intensity of the burden. Trend maps were also created to visualize the EAPC in these metrics over the study period, highlighting regions with increasing or decreasing disease burdens. In the world map, red color represents the highest rate, while blue color represents the lowest rate. The rate gradually decreases from red to yellow, green, and blue. Line graphs were used to depict the trends in ASIR, ASPR, and ASDR from 1992 to 2021, with separate lines for different SDI regions and sexes. Joinpoint regression results were visualized with segmented line plots, showing periods of significant changes in trends. BAPC and ARIMA model projections were illustrated using line graphs with confidence intervals, indicating the predicted trends from 2022 to 2036. Decomposition analysis results were visualized using stacked bar charts and line plots, illustrating the contributions of various factors to changes in otitis media burden. Age-specific burden visualizations were created using line graphs and bar charts, showing the distribution of disease burden across different age groups. Deviation plots were used to illustrate the differences between observed and expected trends in incidence, prevalence, and DALYs across different periods and cohorts.

Except for the plots of the Joinpoint regression analysis, other visualizations were created using the R statistical software, with packages (such as “ggplot2,” “ggmap,” etc.) used to produce high-quality graphics. The plot of the joinpoint regression analysis was drawn by the Joinpoint software (version 5.3.0).

## Results

### Incidence, ASIR, and trends of otitis media from 1992 to 2021

The global number of otitis media cases rose from 322.1 million in 1992 to 391.3 million in 2021, with a slight increase in ASIR from 5345.09 to 5529.1 per 100,000 population (Table 1). This trend corresponds to a global EAPC of 0.11%, indicating relatively stable incidence rates over the nearly three-decade period. For males, ASIR rose slightly from 5090.68 to 5315.08 per 100,000, with an EAPC of just 0.01%. Similarly, females showed an almost negligible change in ASIR, moving from 5608.49 to 5750.94 per 100,000, resulting in an EAPC of 0%. In low and low-middle SDI regions, despite an increase in the absolute number of cases, ASIR declined slightly, leading to negative EAPCs of −0.03% and −0.02%, respectively. Conversely, middle and high-middle SDI regions experienced slight increases in ASIR, with EAPCs of 0.1 and 0.02%, respectively, indicating a steady or marginally increasing disease burden. Among the specific regions, the high-income Asia Pacific region stands out with a significant increase in ASIR from 4036.8 to 4276.84 per 100,000, along with an EAPC of 0.17%. Supplementary Table S1 depicts the cases of incidence and ASIR of otitis media and its temporal trends from 1992 to 2021 among 204 countries and territories.

**Table 1:** The cases of incidence and ASIR of otitis media in 1992 and 2021, and trends from 1992 to 2021.

Characteristics	1992		2021		1992–2021
	Cases of incidence (95%UI)	ASIR per 100,000 (95%UI)	Cases of incidence (95%UI)	ASIR per 100,000 (95%UI)	EAPC (95%CI)
Global	322,122,442 (237,750,281,439,782,053)	5345.09 (3984.29,7243.63)	391,325,570 (292,407,884,525,454,764)	5529.1 (4104.7,7511.93)	0.11 (0.09 to 0.13)
Male	157,647,708 (115,901,304,216,036,150)	5090.68 (3767.97,6936.41)	192,459,644 (142,609,587,260,988,842)	5315.08 (3920.83,7247.72)	0.14 (0.12 to 0.17)
Female	164,474,733 (123,329,797,223,012,386)	5608.49 (4222.55,7553.09)	198,865,926 (150,130,997,265,522,912)	5750.94 (4314.37,7768.79)	0.08 (0.06 to 0.10)
Low SDI	49,279,184 (35,472,046,68,372,423)	6180.68 (4525.38,8458.52)	92,117,447 (66,421,209,127,520,737)	6133.79 (4494.81,8374.16)	−0.03 (−0.03 to −0.02)
Low-middle SDI	96,832,017 (69,762,801,134,806,512)	6073.23 (4461.65,8291.61)	118,637,206 (86,901,144,161,816,363)	6048.17 (4439.74,8243.27)	−0.02 (−0.1 to 0.06)
Middle SDI	99,047,110 (73,527,363,135,262,738)	5039.67 (3782.36,6804.03)	105,611,414 (79,922,870,141,146,871)	5181.53 (3858.97,7027.36)	0.1 (−0.01 to 0.2)
High-middle SDI	45,074,954 (34,530,310,59,693,349)	4585.03 (3487.96,6122.75)	42,502,366 (33,261,061,55,362,032)	4605.87 (3494.03,6167.44)	0.02 (−0.1 to 0.15)
High SDI	31,643,968 (25,534,106,39,865,431)	4500.95 (3548.15,5760.22)	32,188,051 (26,424,578,39,784,782)	4619.95 (3655.99,5920.52)	0.07 (−0.04 to 0.18)
Andean Latin America	2,590,506 (1,888,037,3,611,924)	5182.69 (3849.48,7,118)	3,284,121 (2,457,377,4,488,735)	5178.6 (3846.33,7111.45)	0 (−0.12 to 0.12)
Australasia	870,838 (657,684,1,130,134)	5043.88 (3752.46,6649.01)	1,101,770 (847,743,1,418,457)	5042.59 (3752.06,6646.18)	0 (−0.15 to 0.14)
Caribbean	2,061,521 (1,527,555,2,787,684)	5181.77 (3867.59,6958.21)	2,148,915 (1,630,687,2,837,704)	5178.41 (3870.89,6932.51)	0 (−0.12 to 0.12)
Central Asia	3,694,217 (2,684,838,5,146,654)	4382.89 (3270.75,5961.58)	4,245,004 (3,154,393,5,798,285)	4380.57 (3270.21,5960.8)	0 (−0.06 to 0.06)
Central Europe	4,137,783 (3,226,830,5,399,075)	3956.71 (3012.33,5218.33)	3,039,906 (2,446,677,3,788,126)	3922.46 (3024.1,5092.75)	−0.01 (−0.06 to 0.05)
Central Latin America	11,753,845 (8,666,777,16,160,404)	5436.89 (4106.28,7353.82)	12,121,570 (9,235,553,16,268,294)	5431.18 (4098.97,7346.72)	0 (−0.12 to 0.12)
Central Sub-Saharan Africa	5,229,160 (3,775,231,7,271,756)	5813.73 (4287.71,7933.18)	11,039,251 (7,964,708,15,364,323)	5813.94 (4287.57,7932.96)	0 (−0.01 to 0.01)
East Asia	49,901,982 (38,168,510,66,641,024)	4161.77 (3186.21,5587.37)	44,204,222 (34,500,878,57,245,038)	4161.14 (3185.68,5589.49)	0 (−0.13 to 0.14)
Eastern Europe	9,016,047 (6,905,854,12,168,320)	4729.67 (3559.47,6427.85)	6,997,730 (5,471,560,9,188,911)	4733.59 (3560.77,6435.95)	0.01 (−0.07 to 0.08)
Eastern Sub-Saharan Africa	19,968,785 (14,271,423,27,797,588)	6,336 (4613.44,8572.44)	36,682,213 (26,199,405,50,679,725)	6323.13 (4613.79,8546.51)	−0.01 (−0.1 to 0.08)
High-income Asia Pacific	5,322,381 (4,223,849,6,712,723)	4036.8 (3102.25,5281.24)	4,449,167 (3,613,911,5,509,432)	4276.84 (3249.37,5598.59)	0.17 (0.01 to 0.34)
High-income North America	9,637,516 (8,223,984,11,333,885)	4070.69 (3439.66,4836.18)	10,350,200 (8,992,457,11,875,073)	4189.18 (3565.25,4940.81)	0.04 (0.03 to 0.06)
North Africa and Middle East	24,538,514 (17,833,210,34,228,130)	5261.93 (3927.52,7190.14)	32,815,165 (24,462,746,44,916,674)	5232.56 (3901.12,7161.98)	−0.02 (−0.1 to 0.07)
Oceania	421,801 (302,005,589,771)	4601.08 (3371.82,6313.21)	791,709 (569,619,1,098,648)	4600.95 (3373.54,6313.74)	0 (−0.12 to 0.12)
South Asia	96,808,594 (69,350,732,135,577,481)	6588.34 (4807.38,9047.38)	113,743,017 (83,401,466,155,471,064)	6606.1 (4817.88,9077.17)	0.01 (−0.06 to 0.08)
Southeast Asia	26,021,283 (19,171,097,35,594,687)	4632.99 (3460.34,6258.75)	28,857,204 (21,475,582,39,052,134)	4666.98 (3426.1,6369.43)	0.02 (−0.06 to 0.11)
Southern Latin America	2,576,312 (1,905,093,3,416,522)	4962.77 (3682.06,6560.15)	2,572,973 (1,963,222,3,372,344)	4959.19 (3678.12,6557.93)	0 (−0.14 to 0.14)
Southern Sub-Saharan Africa	4,287,537 (3,125,747,5,950,320)	6167.6 (4565.96,8390.33)	5,042,872 (3,716,938,6,864,688)	6156.35 (4555.24,8367.61)	−0.01 (−0.02 to 0.01)
Tropical Latin America	9,783,739 (7,276,595,13,347,552)	5661.65 (4241.87,7673.58)	10,652,185 (8,116,963,14,161,356)	5656.9 (4241.6,7671.86)	0 (−0.12 to 0.12)
Western Europe	14,758,284 (11,460,492,19,265,494)	5401.18 (4081.23,7312.97)	14,742,413 (11,546,753,19,040,425)	5419.03 (4093.69,7336.8)	0.02 (−0.18 to 0.22)
Western Sub-Saharan Africa	18,741,796 (13,569,087,25,819,044)	6051.3 (4467.43,8223.45)	42,443,961 (30,699,937,59,067,769)	6063.55 (4474.64,8246.03)	0.01 (−0.01 to 0.02)

ASIR, age-standardized incidence rate; EAPC, estimated annual percentage changes; SDI, Socio-demographic index; CI, confidence interval; UI, uncertainty interval.

**Figure 1:**
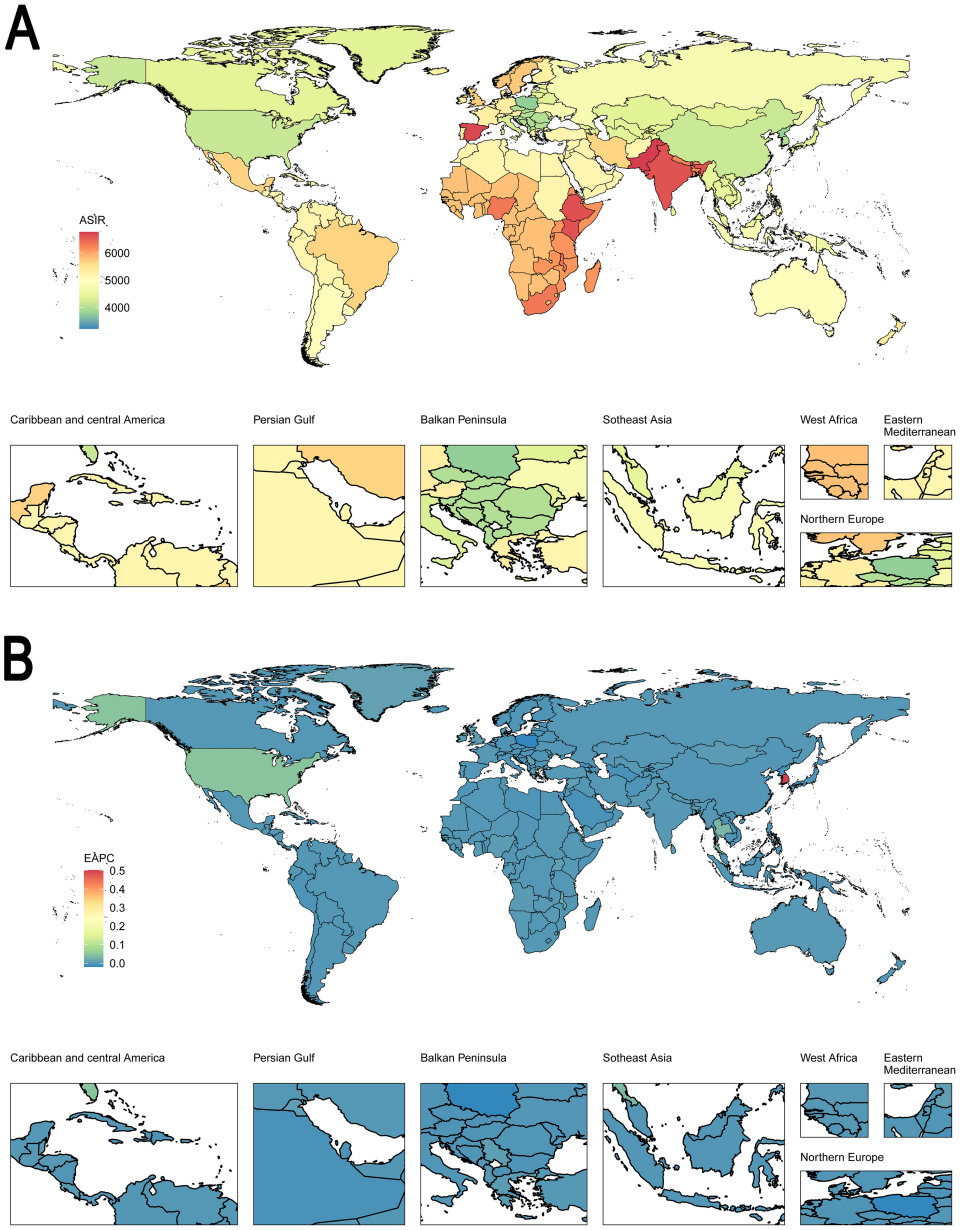
Global distribution in ASIR and trends in EAPC for both sexes in 204 countries or territories. **(A)** The ASIR per 100,000 in 2021. **(B)** The EAPC of ASIR from 1992 to 2021. **(A)** Highlights the geographic distribution of ASIR, with regions in red showing higher incidence rates and those in blue showing lower rates. Panel B displays the trends in ASIR, with blue regions indicating stable or decreasing rates and red regions indicating slight increases. This figure underscores the geographic variability in disease incidence and trends over time. ASIR, age-standardized incidence rate. EAPC, estimated annual percentage change.

In Figure 1A, the map shows the global distribution of ASIR in 2021, with varying intensities across different regions. Higher ASIR values are observed in parts of West Africa, Africa Asia, and South Asia, depicted in red, indicating regions with a higher incidence of the condition. In contrast, regions such as North America, Northern Europe, and parts of Southeast Asia show lower ASIR values, depicted in green, suggesting lower incidence rates. In Figure 1B, the map represents the EAPC of ASIR across the globe from 1992 to 2021. Regions shaded in blue demonstrate a stable or decreasing trend in ASIR, while regions with warmer colors, such as parts of North America and South Korea, indicate a slight increase in ASIR. Most regions globally show minimal changes in ASIR, signifying stable incidence rates over the observed period.

### Prevalence, ASPR, and trends of otitis media from 1992 to 2021

The number of prevalent cases increased from 102.5 million in 1992 to 121.2 million in 2021 (Table 2). Despite this increase in cases, the age-standardized prevalence rate (ASPR) per 100,000 population decreased from 1786.56 to 1593.74, reflecting an EAPC of −0.43%. For males, the ASPR decreased from 1879.04 in 1992 to 1672.3 in 2021, with an EAPC of −0.37%. Similarly, females experienced a decline in ASPR from 1693.1 to 1513.31, also resulting in an EAPC of −0.37%. In low SDI regions, the ASPR decreased slightly from 2231.33 to 2046.25, with an EAPC of −0.36%. However, the most significant decreases were observed in middle and high-middle SDI regions, where ASPR dropped from 1843.65 to 1513.89 and from 1548.36 to 1276.52, respectively, corresponding to EAPCs of −0.7% and −0.77%. Regionally, East Asia exhibited a notable decrease in ASPR from 1821.09 to 1335.55, with an EAPC of −1.14%, the largest decline among all regions. Similarly, Southeast Asia saw a significant reduction in ASPR from 1732.53 to 1433.26, resulting in an EAPC of −0.67%. Supplementary Table S2 depicts the cases of prevalence and ASPR of otitis media in 1992 and 2021, and its temporal trends from 1992 to 2021 among 204 countries and territories.

**Table 2:** The cases of prevalence and ASPR of otitis media in 1992 and 2021, and trends from 1992–2021.

Characteristics	1992		2021		1992–2021
	Cases of prevalence (95%UI)	ASPR per 100,000 (95%UI)	Cases of prevalence (95%UI)	ASPR per 100,000 (95%UI)	EAPC (95%CI)
Global	102,513,402 (88,326,416,118,618,455)	1786.56 (1534.22,2056.93)	121,229,852 (104,258,337,139,980,026)	1593.74 (1374.43,1833.01)	−0.43 (−0.44 to −0.41)
Male	54,703,010 (46,985,015,63,346,985)	1879.04 (1616.23,2160.97)	64,565,602 (55,461,984,74,332,380)	1672.3 (1443.28,1925.41)	−0.43 (−0.44 to −0.42)
Female	47,810,392 (41,287,775,55,318,928)	1693.1 (1452.38,1954.98)	56,664,250 (48,898,255,65,716,751)	1513.31 (1307.78,1744.19)	−0.42 (−0.43 to −0.41)
Low SDI	13,034,395 (11,227,575,15,235,822)	2231.33 (1888.88,2586.41)	25,194,728 (21,696,551,29,669,180)	2046.25 (1733.15,2366.82)	−0.36 (−0.47 to −0.25)
Low-middle SDI	29,248,318 (25,230,403,34,167,686)	2206.59 (1881.77,2550.25)	37,354,173 (31,921,091,43,295,998)	1889.25 (1617.56,2177.85)	−0.54 (−0.64 to −0.45)
Middle SDI	34,520,840 (29,490,228,40,125,761)	1843.65 (1586.73,2125.59)	34,916,351 (30,012,675,40,524,529)	1513.89 (1313.16,1746.04)	−0.7 (−0.81 to −0.6)
High-middle SDI	16,506,835 (14,136,335,19,280,752)	1548.36 (1334.11,1787.32)	14,502,040 (12,465,670,16,968,871)	1276.52 (1110.72,1472.22)	−0.77 (−0.87 to −0.66)
High SDI	9,123,276 (7,882,424,10,612,437)	1119.33 (979.27,1287.2)	9,176,520 (7,886,400,10,615,702)	1014.96 (885.51,1158.07)	−0.36 (−0.42 to −0.3)
Andean Latin America	683,991 (594,293,786,548)	1601.96 (1382.82,1857.86)	937,552 (808,891,1,088,334)	1429.39 (1235.47,1652.36)	−0.49 (−0.58 to −0.4)
Australasia	163,451 (140,130,189,580)	871.24 (749.39,1008.06)	205,545 (177,322,237,215)	811.13 (704.01,938.35)	−0.27 (−0.37 to −0.18)
Caribbean	601,537 (519,551,699,580)	1602.87 (1377.97,1843.31)	717,693 (615,272,835,395)	1570.79 (1355.97,1819.98)	−0.14 (−0.23 to −0.05)
Central Asia	1,140,686 (975,673,1,326,414)	1520.88 (1294.7,1775.18)	1,344,592 (1,156,070,1,571,027)	1397.8 (1200.86,1629.87)	−0.51 (−0.65 to −0.37)
Central Europe	1,674,413 (1,424,386,1,953,751)	1405.03 (1202.69,1626.52)	1,195,044 (1,016,231,1,409,203)	1212.22 (1040.46,1406.69)	−0.6 (−0.72 to −0.48)
Central Latin America	2,968,148 (2,603,032,3,397,830)	1563.05 (1365.73,1807)	3,626,112 (3,153,681,4,198,956)	1483.23 (1295.56,1704.76)	−0.23 (−0.31 to −0.14)
Central Sub-Saharan Africa	1,264,503 (1,084,738,1,474,242)	1942.79 (1639.75,2263.82)	3,028,765 (2,582,621,3,546,949)	2028.76 (1715.76,2362.44)	0.03 (−0.11 to 0.17)
East Asia	23,091,644 (19,490,980,27,248,560)	1821.09 (1561.82,2114.17)	17,366,491 (15,000,397,20,341,720)	1335.55 (1162.35,1545.94)	−1.14 (−1.26 to −1.03)
Eastern Europe	3,121,607 (2,723,496,3,613,566)	1476.02 (1289.34,1698.72)	2,498,537 (2,167,948,2,892,524)	1414.91 (1236.75,1625.44)	−0.33 (−0.46 to −0.21)
Eastern Sub-Saharan Africa	4,661,569 (4,052,781,5,447,968)	2051.08 (1737.29,2366.79)	8,948,542 (7,715,592,10,493,169)	1878.28 (1604.28,2180.55)	−0.39 (−0.51 to −0.27)
High-income Asia Pacific	1,799,067 (1,532,871,2,110,147)	1119.91 (971.95,1300.1)	1,485,143 (1,259,499,1,723,831)	1007.34 (871.46,1160.8)	−0.34 (−0.38 to −0.3)
High-income North America	2,578,533 (2,256,903,2,945,537)	985.34 (868.89,1110.49)	2,882,329 (2,507,884,3,276,271)	933.67 (822.78,1051.56)	−0.23 (−0.31 to −0.15)
North Africa and Middle East	6,590,105 (5,704,128,7,673,889)	1674.02 (1441.23,1945.08)	9,648,651 (8,286,391,11,218,087)	1527.13 (1317.92,1775.13)	−0.42 (−0.49 to −0.35)
Oceania	123,886 (106,053,144,024)	1644.12 (1403.03,1903.03)	233,647 (201,183,273,336)	1564.23 (1332.02,1817.08)	−0.12 (−0.23 to −0.01)
South Asia	30,226,114 (25,899,767,35,278,441)	2419.82 (2062.45,2799.59)	38,054,916 (32,324,529,44,386,663)	2048.28 (1755.16,2369.76)	−0.53 (−0.59 to −0.48)
Southeast Asia	8,897,140 (7,646,374,10,302,329)	1732.53 (1481.51,1990.62)	9,667,713 (8,291,700,11,261,955)	1433.26 (1235.42,1661.23)	−0.67 (−0.78 to −0.56)
Southern Latin America	615,200 (528,889,709,772)	1198.81 (1031.91,1387.08)	662,875 (565,808,776,441)	1080.67 (931.66,1249.58)	−0.39 (−0.42 to −0.37)
Southern Sub-Saharan Africa	1,055,655 (919,606,1,222,920)	1713.8 (1492.68,1959.93)	1,364,778 (1,184,872,1,577,501)	1661.57 (1445.55,1910.02)	−0.13 (−0.25 to −0.02)
Tropical Latin America	2,512,931 (2,183,043,2,927,978)	1517.92 (1320.03,1757.15)	3,018,830 (2,609,128,3,533,311)	1423.13 (1239.68,1655.18)	−0.23 (−0.31 to −0.16)
Western Europe	3,955,821 (3,358,103,4,628,329)	1150.88 (988.3,1329.85)	3,851,251 (3,247,538,4,525,785)	1051.98 (904.93,1214.86)	−0.3 (−0.33 to −0.28)
Western Sub-Saharan Africa	4,787,401 (4,126,108,5,599,683)	2115.45 (1789.24,2470.13)	10,490,846 (9,084,206,12,240,956)	1913.38 (1622.44,2213.17)	−0.51 (−0.63 to −0.39)

In Supplementary Figure S1A, the map shows the global distribution of ASPR, with higher prevalence rates observed in regions such as East Africa, West Africa, and Central African, depicted in red, indicating a greater burden of disease in these areas. In contrast, regions in Australia and North America exhibit lower ASPR values, indicated in blue, suggesting lower disease prevalence. In Supplementary Figure S1B, the map presents the EAPC of ASPR globally, indicating how the prevalence of the condition has changed over time. Regions shaded in red, including parts of North Africa, Central Africa, and East Africa, indicate an increasing trend in prevalence, while regions in green, such as East Asia and parts of Southeast Asia, show a decreasing trend, signifying a reduction in disease burden over time. The majority of the world shows minimal changes in prevalence, represented by shades of yellow, reflecting stable disease prevalence over the observed period.

### DALYs, ASDR, and trends of otitis media from 1992 to 2021

The number of DALYs increased from 2.16 million in 1992 to 2.48 million in 2021, yet the ASDR decreased from 37.68 to 32.54 per 100,000 population, resulting in an EAPC of −0.51% (Table 3). For males, ASDR declined from 39.91 in 1992 to 34.49 in 2021, with an EAPC of −0.48%. Similarly, females saw a reduction in ASDR from 35.42 to 30.52, also resulting in an EAPC of −0.48%. Low and low-middle SDI regions experienced significant declines in ASDR, with low SDI regions showing a decrease from 50.77 to 43.25 per 100,000 population, and an EAPC of −0.6%. Similarly, low-middle SDI regions had a reduction in ASDR from 46.31 to 38.62, resulting in an EAPC of −0.61%. The most pronounced decrease was observed in middle SDI regions, where ASDR fell from 38.47 to 30.52, corresponding to an EAPC of −0.78%. High-middle SDI regions also saw a significant reduction, with ASDR declining from 31.53 to 25.31, resulting in an EAPC of −0.85%. East Asia experienced the most significant decline in ASDR, dropping from 37.07 in 1992 to 26.59 in 2021, with an EAPC of −1.22%. Southeast Asia also showed a considerable decrease, with ASDR falling from 35.56 to 29.05, resulting in an EAPC of −0.71%.

**Table 3:** The cases of DALYs and ASDR of otitis media in 1992 and 2021, and trends from 1992 to 2021.

Characteristics	1992		2021		1992–2021
	Cases of DALYs (95%UI)	ASDR per 100,000 (95%UI)	Cases of DALYs (95%UI)	ASDR per 100,000 (95%UI)	EAPC (95%CI)
Global	2,162,289 (1,297,828,3,442,163)	37.68 (22.69,59.66)	2,479,623 (1,458,845,3,973,787)	32.54 (19.21,51.87)	−0.51 (−0.52 to −0.5)
Male	1,162,331 (692,711,1,853,828)	39.91 (23.69,63.22)	1,333,946 (784,893,2,140,127)	34.49 (20.36,55.24)	−0.51 (−0.52 to −0.5)
Female	999,958 (596,936,1,589,043)	35.42 (21.12,56.1)	1,145,677 (674,450,1,833,833)	30.52 (17.96,48.42)	−0.52 (−0.53 to −0.51)
Low SDI	305,439 (180,872,469,688)	50.77 (30.47,78.14)	533,422 (316,709,847,792)	43.25 (25.65,68.72)	−0.6 (−0.73 to −0.47)
Low-middle SDI	612,143 (358,635,974,593)	46.31 (27.4,73.81)	765,766 (447,966,1,233,392)	38.62 (22.57,61.92)	−0.61 (−0.72 to −0.5)
Middle SDI	719,828 (429,475,1,160,180)	38.47 (23.09,61.17)	707,715 (414,797,1,144,340)	30.52 (17.86,49.01)	−0.78 (−0.91 to −0.66)
High-middle SDI	337,021 (199,273,541,086)	31.53 (18.82,50.22)	290,348 (169,731,467,565)	25.31 (14.59,40.61)	−0.85 (−0.98 to −0.73)
High SDI	186,152 (112,165,293,545)	22.65 (13.51,35.67)	180,627 (106,868,288,748)	19.52 (11.23,31.04)	−0.49 (−0.58 to −0.4)
Andean Latin America	13,146 (7,579,21,093)	31.22 (18.21,50.44)	18,206 (10,687,29,182)	27.66 (16.28,44.45)	−0.52 (−0.62 to −0.42)
Australasia	3,135 (1812,5,013)	16.51 (9.51,26.31)	3,884 (2,268,6,125)	15 (8.6,24.05)	−0.37 (−0.47 to −0.28)
Caribbean	11,928 (6,927,19,245)	31.86 (18.63,51.09)	14,308 (8,376,22,926)	31.15 (18.26,49.66)	−0.17 (−0.27 to −0.06)
Central Asia	23,073 (13,402,37,439)	30.87 (18.1,50)	27,055 (15,684,44,045)	28.12 (16.33,45.7)	−0.56 (−0.71 to −0.4)
Central Europe	38,053 (24,051,59,166)	33.17 (21.54,50.63)	24,172 (14,105,38,971)	24.47 (14.22,39.46)	−0.94 (−1.09 to −0.8)
Central Latin America	65,212 (41,066,101,440)	34.5 (21.62,53.29)	72,412 (42,595,115,634)	29.42 (17.24,46.9)	−0.49 (−0.6 to −0.37)
Central Sub-Saharan Africa	26,142 (15,288,41,371)	40.22 (23.78,64.49)	62,171 (35,834,99,436)	41.76 (24.35,67.11)	0.01 (−0.14 to 0.17)
East Asia	471,682 (277,430,767,098)	37.07 (21.97,59.01)	349,271 (204,353,567,697)	26.59 (15.36,43.02)	−1.22 (−1.35 to −1.08)
Eastern Europe	63,670 (37,658,102,876)	30.04 (17.73,47.96)	50,477 (29,616,81,838)	28.56 (16.73,45.57)	−0.37 (−0.5 to −0.23)
Eastern Sub-Saharan Africa	132,349 (75,187,202,878)	53.7 (30.76,80.55)	198,384 (114,434,308,495)	41.64 (24.34,65.09)	−0.94 (−1.09 to −0.79)
High-income Asia Pacific	35,675 (20,978,58,624)	21.8 (12.68,35.49)	29,187 (17,171,47,364)	19.22 (10.96,30.76)	−0.4 (−0.45 to −0.34)
High-income North America	52,280 (31,997,82,727)	19.77 (11.83,31.48)	56,987 (34,267,91,067)	18.06 (10.51,29.04)	−0.33 (−0.42 to −0.24)
North Africa and Middle East	133,643 (78,618,213,594)	34.03 (19.86,54.23)	195,300 (114,028,307,934)	30.84 (17.98,48.66)	−0.44 (−0.53 to −0.36)
Oceania	2,471 (1,453,3,997)	33.05 (19.41,53.48)	4,653 (2,673,7,573)	31.33 (18.19,50.87)	−0.12 (−0.26 to 0.01)
South Asia	634,510 (373,686,1,009,927)	51 (30.35,81.38)	781,634 (455,249,1,265,214)	41.85 (24.42,67.34)	−0.61 (−0.68 to −0.54)
Southeast Asia	182,762 (107,122,295,577)	35.56 (20.84,57.07)	196,705 (113,452,317,617)	29.05 (16.77,46.71)	−0.71 (−0.84 to −0.58)
Southern Latin America	12,090 (7,122,19,226)	23.59 (13.89,37.43)	12,839 (7,609,20,436)	20.6 (12.01,33)	−0.5 (−0.55 to −0.45)
Southern Sub-Saharan Africa	21,309 (12,800,33,945)	34.88 (20.9,55.56)	27,309 (16,249,43,701)	33.19 (19.87,52.88)	−0.18 (−0.32 to −0.05)
Tropical Latin America	58,170 (37,368,89,682)	35.25 (22.83,53.47)	62,084 (37,253,99,215)	29.12 (17.26,46.23)	−0.4 (−0.53 to −0.26)
Western Europe	81,023 (48,951,125,160)	23.33 (14.05,36.05)	74,916 (44,383,120,255)	19.81 (11.46,31.23)	−0.5 (−0.57 to −0.43)
Western Sub-Saharan Africa	99,968 (57,639,159,247)	44.21 (25.72,71.47)	217,669 (127,418,348,524)	39.68 (23.31,63.79)	−0.55 (−0.69 to −0.42)

DALYs, disability-adjusted life years; ASDR, age-standardized DALYs rate; EAPC, estimated annual percentage changes; SDI, Socio-demographic index; CI, confidence interval; UI, uncertainty interval.

In contrast, certain regions showed more moderate changes. Central Sub-Saharan Africa, for instance, exhibited a slight increase in ASDR from 40.22 to 41.76 per 100,000, with an EAPC of 0.01%, indicating a stable or slightly increasing burden. Other regions, like Central Europe and Eastern Sub-Saharan Africa, saw significant decreases in ASDR, with EAPCs of −0.94% and −0.94%, respectively, reflecting successful efforts in reducing the burden of otitis media. Supplementary Table S3 depicts the cases of incidence and ASDR of otitis media in 1992 and 2021, and its temporal trends from 1992 to 2021 among 204 countries and territories.

**Figure 2:**
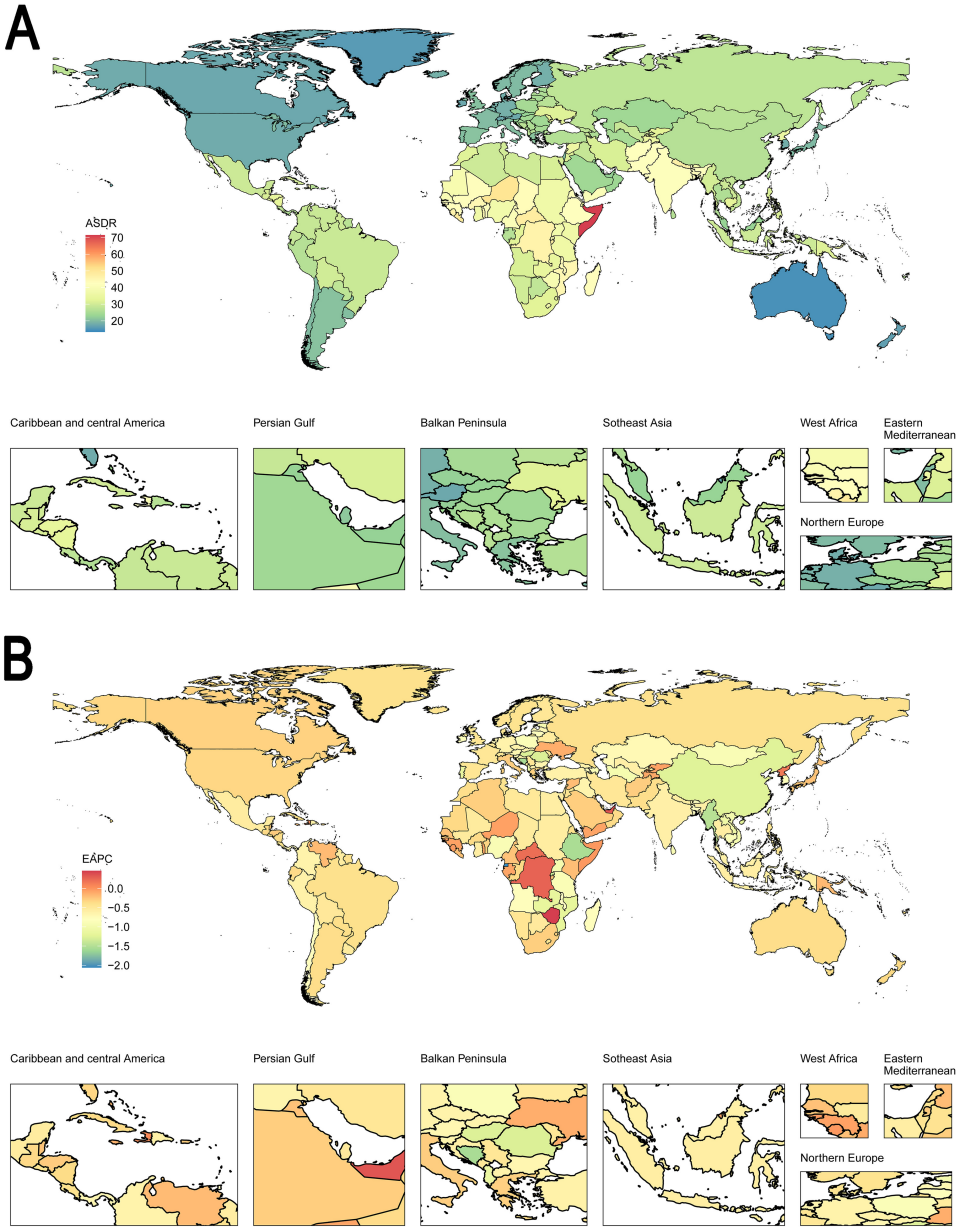
Global distribution in ASDR and trends in EAPC for both sexes in 204 countries or territories. **(A)** The ASDR per 100,000 in 2021. **(B)** The EAPC of ASDR from 1992 to 2021. **(A)** Shows the ASDR distribution globally, with regions in red representing higher DALY rates and those in blue indicating lower rates. **(B)** Illustrates the trends in DALYs, with red regions showing an increasing trend in disease burden and blue regions indicating a decline. This figure highlights the global variability in the burden of disease and temporal trends, providing insights for targeted public health interventions. ASDR, age-standardized DALY rate. EAPC, estimated annual percentage change. DALYs, disability-adjusted life years.

In Figure 2A, the map shows the distribution of ASDR across the globe, with regions such as East Africa, West Africa, and Central African showing higher DALY rates (indicated in red), reflecting a higher burden of disease in these areas. In contrast, regions in Australia and North America exhibit lower DALY rates (indicated in blue), suggesting a lower disease burden. In Figure 2B, the map presents the global trends in the EAPC of DALYs. Regions shaded in red, particularly in parts of Central Africa and East Africa, indicate an increasing trend in the burden of disease, while regions in green, such as East Asia and parts of Southeast Asia, demonstrate a decreasing trend, indicating an improvement in health outcomes over time. Most regions show minimal changes in DALYs, represented by shades of yellow, suggesting slight lower disease burdens.

### Incidence, prevalence, and DALYs of otitis media in different ages, year, and SDI

**Figure 3:**
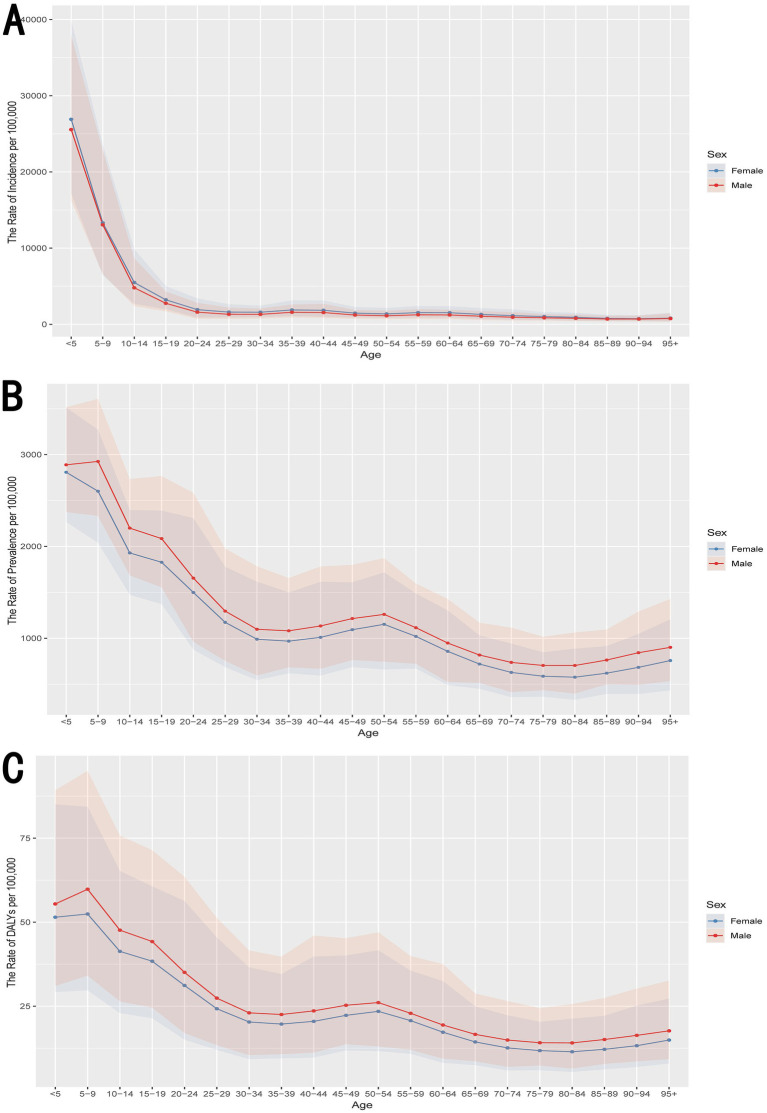
Age-specific rates of otitis media on incidence, prevalence, and DALYs. **(A)** Incidence. **(B)** Prevalence. **(C)** DALYs. The figures demonstrate that the burden of otitis media, in terms of incidence, prevalence, and DALYs, is significantly higher in younger age groups, particularly in males. The burden declines with age, with some variation observed in older age groups. The shaded areas represent the 95% confidence intervals. DALYs, disability-adjusted life years.

In terms of age, Figure 3A showed that the incidence rate per 100,000 population is highest in the youngest age group (<5 years), with a steep decline as age increases. This pattern is consistent for both males and females, though males tend to have slightly higher incidence rates across most age groups. Figure 3B depicts the prevalence rates per 100,000 population, which are also highest among children under 10 years of age. The prevalence decreases steadily as age progresses, with a slight increase observed in older age groups (70–79 years), suggesting a bimodal distribution. Similar to incidence, males generally exhibit higher prevalence rates than females. Figure 3C shows the age-specific rates of DALYs per 100,000 population, with the highest burden observed in children aged 5–9 years. The DALY rates decrease with age but exhibit less steep declines compared to incidence and prevalence, indicating a sustained burden of otitis media across older age groups. Males consistently show higher DALY rates across almost all age groups compared to females.

In terms of year, Supplementary Figure S2A demonstrates a gradual decline in the incidence rate per 100,000 population over the study period, with males consistently exhibiting lower incidence rates compared to females. Supplementary Figure S2B shows a similar downward trend in the prevalence rate per 100,000 population, with males maintaining higher prevalence rates throughout the period compared to females. Supplementary Figure S2C depicts the trend in DALYs per 100,000 population, revealing a continuous decrease across the years. Males consistently have higher DALY rates compared to females, though both sexes exhibit a notable reduction in the burden of otitis media as measured by DALYs.

In terms of SDI, Supplementary Figure S3 shifts the focus to socio-demographic differences. Supplementary Figure S3A demonstrates that the number of incidence cases is highest in low-middle SDI regions, with a slightly higher burden observed in males. Similarly, Supplementary Figure S3B shows that prevalence is also most significant in low and low-middle SDI regions, reflecting the widespread impact of otitis media in these socio-economic settings. Supplementary Figure S3C highlights that DALYs, representing the disease’s impact on quality of life, are predominantly higher in low and low-middle SDI regions.

### Joinpoint regression analysis

**Figure 4:**
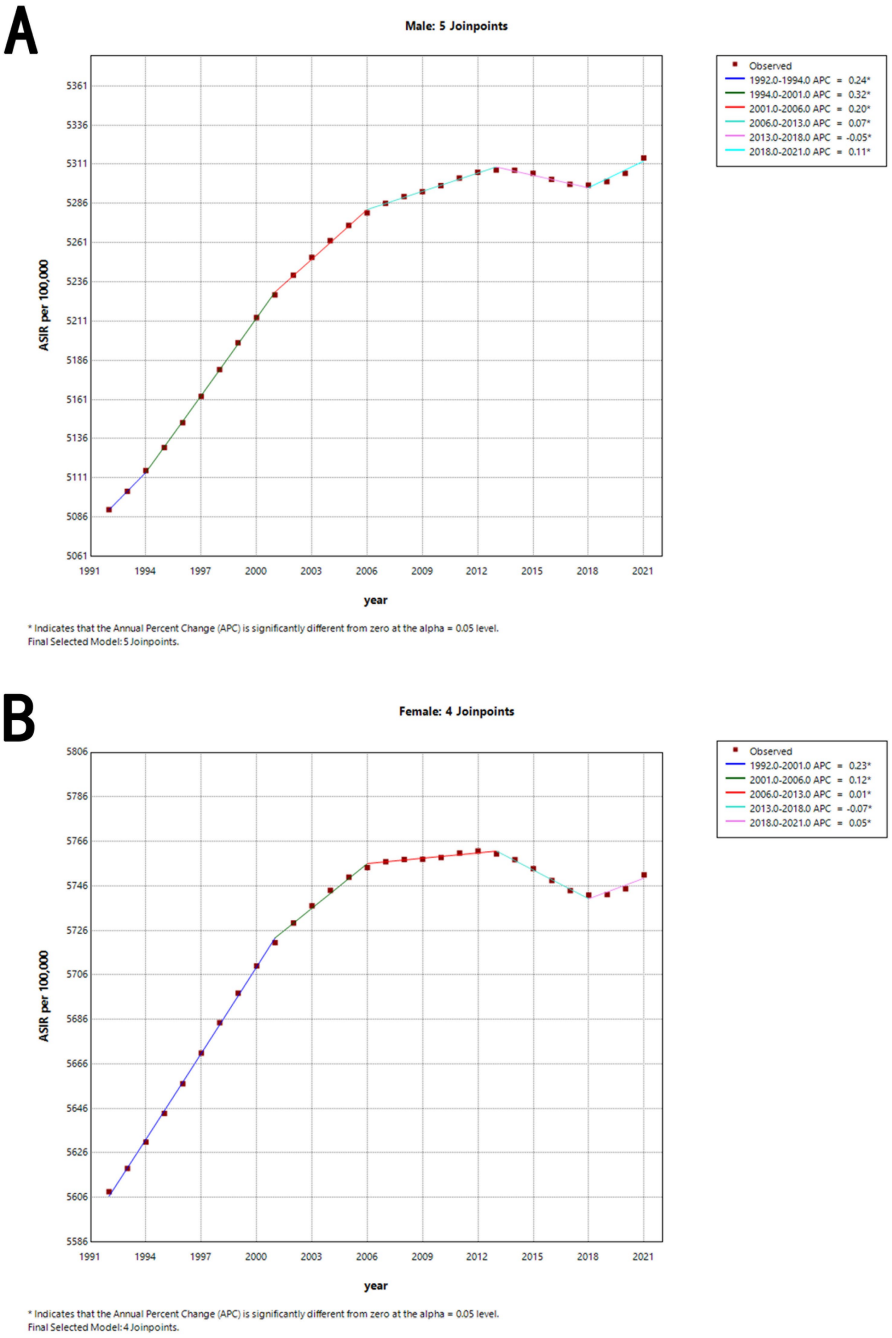
Joinpoint regression analysis of the sex-specific ASIR for otitis media from 1992 to 2021. **(A)** ASIR for males. **(B)** ASIR for females. The graphs show periods of significant change in ASIR, identified through joinpoint regression analysis, with the APC values indicating the rate of change during each period. ASIR, age-standardized incidence rate. APC, annual percentage change.

For ASIR, Figure 4A shows that for males, the ASIR increased until the early 2000s, followed by periods of stabilization and slight fluctuations, with a more recent uptick observed from 2018 onwards. Figure 4B indicates that the ASIR for females followed a similar trajectory, with a peak around 2006, followed by a slight decline, and then a mild increase in recent years. The analysis highlights key periods where the annual percentage change (APC) is significantly different from zero, indicating distinct shifts in incidence trends over time.

For ASPR, Supplementary Figure S4A shows that for males, the most significant decrease occurred between 1992 and 2018, with a slight deceleration in the decline afterward. Supplementary Figure S4B indicates that the ASPR for females showed a steady reduction in prevalence rates, with notable drops occurring in the early 2000s and continuing through 2018.

For ASDR, Supplementary Figure S5A shows that for males, the most pronounced decreases were observed in the late 1990s and early 2000s, with a gradual slowing of the decline in recent years. Supplementary Figure S5B shows that the female pattern mirrors that of males, showing consistent reductions in ASDR with key periods of change, particularly between 1992 and 2010.

The detailed data is shown in Table 4.

**Table 4:** Joinpoint regression analysis: trends in age-standardized incidence, prevalence, DALYs (per 100,000 persons) among both sexes, males, and females, 1992–2021.

Gender	ASIR			ASPR			ASDR		
	Period	APC (95%CI)	AAPC (95%CI)	Period	APC (95%CI)	AAPC (95%CI)	Period	APC (95%CI)	AAPC (95%CI)
Both	1992–1994	0.22 (0.18–0.25)	0.12 (0.11–0.12)	1992–1996	−0.29 (−0.32 to −0.26)	−0.39 (−0.41 to −0.38)	1992–1995	−0.54 (−0.62 to −0.47)	−0.51 (−0.52 to −0.49)
	1994–2001	0.27 (0.27–0.28)		1996–1999	−0.48 (−0.57 to −0.39)		1995–1999	−0.73 (−0.80 to −0.65)	
	2001–2006	0.16 (0.15–0.17)		1999–2005	−0.33 (−0.35 to −0.31)		1999–2005	−0.41 (−0.44 to −0.38)	
	2006–2013	0.04 (0.04–0.05)		2005–2010	−0.52 (−0.54 to −0.49)		2005–2018	−0.53 (−0.54 to −0.52)	
	2013–2018	−0.06 (−0.07 to −0.05)		2010–2018	−0.49 (−0.50 to −0.48)		2018–2021	−0.27 (−0.34 to −0.20)	
	2018–2021	0.08 (0.06–0.10)		2018–2021	−0.13 (−0.17 to −0.09)				
Female	1992–2001	0.23 (0.22–0.23)	0.09 (0.08–0.09)	1992–1996	−0.27 (−0.29 to −0.24)	−0.39 (−0.40 to −0.38)	1992–1995	−0.55 (−0.60 to −0.50)	−0.51 (−0.52 to −0.50)
	2001–2006	0.12 (0.10–0.13)		1996–1999	−0.43 (−0.51 to −0.36)		1995–1999	−0.72 (−0.77 to −0.67)	
	2006–2013	0.01 (0.01–0.02)		1999–2005	−0.33 (−0.34 to −0.31)		1999–2006	−0.44 (−0.45 to −0.42)	
	2013–2018	−0.07 (−0.09 to −0.06)		2005–2014	−0.51 (−0.52 to −0.50)		2006–2018	−0.53 (−0.54 to −0.53)	
	2018–2021	0.05 (0.03–0.08)		2014–2018	−0.47 (−0.51 to −0.44)		2018–2021	−0.29 (−0.34 to −0.24)	
				2018–2021	−0.14 (−0.17 to −0.10)				
Male	1992–1994	0.24 (0.19–0.28)	0.15 (0.14–0.15)	1992–1996	−0.32 (−0.35 to −0.28)	−0.40 (−0.42 to −0.39)	1992–1995	−0.55 (−0.65 to −0.44)	−0.50 (−0.53 to −0.48)
	1994–2001	0.32 (0.31–0.32)		1996–1999	−0.52 (−0.63 to −0.41)		1995–1999	−0.73 (−0.84 to −0.62)	
	2001–2006	0.20 (0.19–0.21)		1999–2005	−0.33 (−0.36 to −0.31)		1999–2005	−0.41 (−0.45 to −0.36)	
	2006–2013	0.07 (0.07–0.08)		2005–2009	−0.53 (−0.58 to −0.47)		2005–2019	−0.52 (−0.53 to −0.51)	
	2013–2018	−0.05 (−0.06 to −0.04)		2009–2018	−0.48 (−0.49 to −0.47)		2019–2021	−0.10 (−0.32–0.12)	
	2018–2021	0.11 (0.09–0.13)		2018–2021	−0.14 (−0.19 to −0.09)				

ASIR, age-standardized incidence rate; ASPR, age-standardized prevalence rate; DALYs, disability-adjusted life years; ASDR, age-standardized DALYs rate; AAPC, average annual percent change presented for full period; APC, annual percent change; CI, confidence interval.

### Age, period, and cohort effects on incidence, prevalence, and DALYs of otitis media

For incidence, Supplementary Figure S6 reveals significant age effects, with the highest incidence in early childhood, and period effects suggesting a peak in risk around the mid-2010s. Cohort effects appear relatively stable, with little variation in risk across different birth cohorts. For prevalence, Supplementary Figure S7 highlights significant age effects, with the highest prevalence in younger age groups, and period effects indicating a decline in prevalence risk over time. Cohort effects suggest a steady decrease in risk for more recent birth cohorts. For DALYs, Supplementary Figure S8 shows significant age effects, with DALYs concentrated in younger age groups, and period effects indicating a steady decline in DALY risk over time. Cohort effects suggest a substantial reduction in risk for more recent birth cohorts, reflecting improvements in healthcare and prevention strategies.

Supplementary Figure S9 presents a comprehensive period-cohort analysis of age-specific period and birth cohort effects on incidence, prevalence, and DALYs of otitis media. It reveals that the highest burden of otitis media is concentrated in the youngest age groups (<5 years), with a consistent decline in incidence, prevalence, and DALYs as age increases. This figure underscores the importance of focusing on younger populations for prevention and intervention strategies.

Supplementary Figure S10 and Supplementary Tables S4–S6 illustrate the age, period, and birth cohort effects on the relative risks (RRs) for incidence, prevalence, and DALYs of otitis media. It reveals that the age effect shows a marked decrease in relative risk as age increases, particularly after early childhood. Period effects are relatively stable, with slight declines observed in prevalence and DALYs. Cohort effects indicate a substantial increase in relative risk for individuals born after 1940, suggesting a higher burden of otitis media in more recent cohorts.

### Decomposition analysis of incidence, prevalence, and DALY rates

Supplementary Figure S11 presents a decomposition analysis of the factors contributing to changes in otitis media incidence, prevalence, and DALYs across different sexes and SDI levels. Supplementary Figures S11A,B highlights that population growth is the primary driver of increased incidence, especially in lower SDI regions. Supplementary Figures S11C,D shows a similar pattern for prevalence, with population growth again being the dominant factor. Supplementary Figures S11E,F indicates that population growth significantly contributes to rising DALYs, particularly in low SDI areas, while aging and epidemiological changes play varying roles depending on sex and SDI.

### Frontier analysis of ASIR, ASPR, and ASDR

Supplementary Figure S12 shows the relationship between SDI and the trends in ASIR, ASPR, and ASDR. Countries with low SDI generally show higher rates, but some have achieved significant improvements over time. Countries like Norway and Sweden show increasing ASIR despite high SDI, whereas countries like Niger show significant decreases. High-SDI countries like Japan maintain lower ASDR rates, while countries like Somalia show significant decreases despite lower SDI. Ireland and Papua New Guinea have diverging trends on ASIR despite similar SDI.

### Health inequality analysis of incidence, prevalence, and DALY rates

Supplementary Figures S13A,C,E depict the slope index of inequality for each health outcome, showing the relationship between the rates and relative SDI rank. Supplementary Figures S13B,D,F illustrate the concentration index, indicating the distribution of health outcomes across different SDI ranks.

### Prediction of ASIR, ASPR, and ASDR from 2022 to 2036

**Figure 5:**
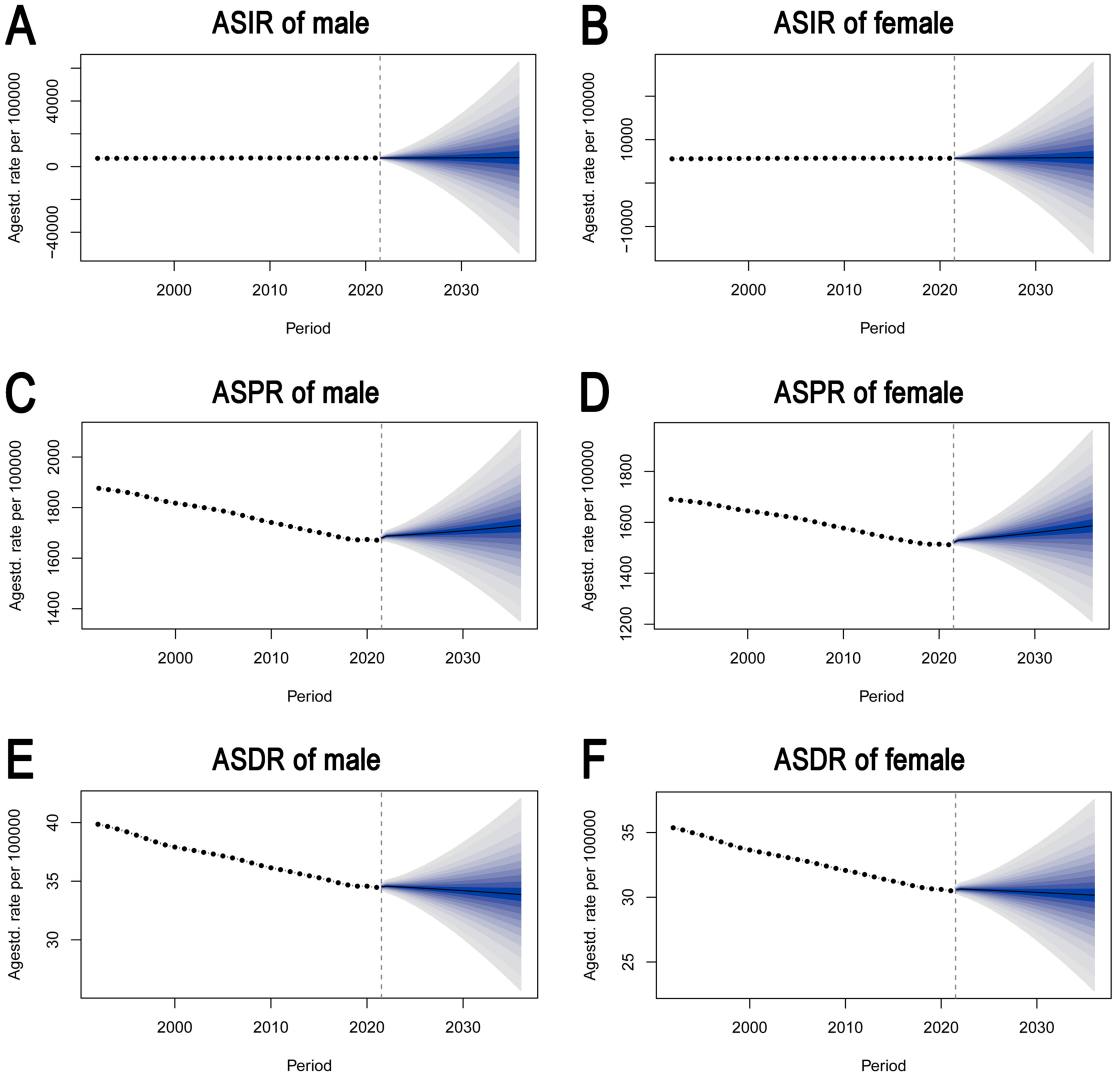
Bayesian age-period-cohort (BAPC) model predictions of ASIR, ASPR, and ASDR for males and females. **(A)** ASIR for males. **(B)** ASIR for females. **(C)** ASPR for males. **(D)** ASPR for females. **(E)** ASDR for males. **(F)** ASDR for females. It highlights the stability of the ASIR and ASPR up to 2020, followed by growing uncertainty in future projections, while ASDR trends continue to decline with widening variability in the future estimates. ASIR, age-standardized incidence rate. ASPR, age-standardized prevalence rate. ASDR, age-standardized DALY rate. DALY, disability-adjusted life year.

Figure 5 illustrates the BAPC model predictions for ASIR, ASPR, and ASDR for both males and females from 2022 to 2036. It highlights the stability of the ASIR and ASPR up to 2020, followed by growing uncertainty in future projections, while ASDR trends continue to decline with widening variability in the future estimates.

Supplementary Figure S14 presents the ARIMA model forecasts for ASIR, ASPR, and ASDR for both males and females from 2022 to 2036. It highlights a projected increase in ASIR and a decrease in ASPR and ASDR for both sexes, with varying degrees of confidence in the predictions.

### The correlation between SDI and the global burden of otitis media

Supplementary Figure S15 suggests that 21 regions or 204 countries (or territories) with higher SDI are associated with lower prevalence rates of ASIR, ASPR, and ASDR.

## Discussion

The present study provides a comprehensive analysis of the global burden of otitis media from 1992 to 2021, with projections up to 2036. Our findings reveal that while the global incidence of otitis media has increased in absolute numbers, ASIR has remained relatively stable, with an EAPC of 0.11%. In contrast, ASPR and ASDR have shown significant declines, with EAPCs of −0.43% and −0.51%, respectively. These trends suggest that, although otitis media remains a common condition globally, improvements in healthcare and preventive measures may have reduced its overall burden. However, disparities persist across different regions and SDI categories, with low and low-middle SDI regions continuing to bear the highest burden of the disease.

The relatively stable trend of ASIR is supported by previous research. For instance, Jin et al. reported a similar stabilization in the incidence of otitis media in high SDI regions, attributing this trend to widespread vaccination programs and improved access to healthcare (13). However, low and low-middle SDI regions continue to experience high rates of otitis media. This divergence highlighted the persistent burden of otitis media in low-resource settings, where access to preventive measures and healthcare services remains limited. The decline in ASPR and ASDR observed in our study is also supported by previous research. Improvements in hygiene, nutrition, and early treatment have contributed to a reduction in the prevalence and severity of otitis media, particularly in middle-income countries (11, 23–25). Our findings suggest that these improvements may have a broader impact, as evidenced by the significant declines in ASPR and ASDR across various regions. However, it is important to note that the magnitude of these declines varies by region, with East Asia and Southeast Asia showing the most pronounced reductions, possibly due to targeted public health interventions and increased awareness of the disease.

The observed trends in otitis media can be attributed to several factors. In high-income regions, widespread vaccination against pneumococcal and *Haemophilus influenzae* type b infections has likely played a crucial role in reducing the incidence and severity of otitis media (15, 26, 27). These vaccines have been shown to significantly reduce the occurrence of middle ear infections, particularly in young children, who are most vulnerable to the disease. Additionally, improvements in healthcare access, early diagnosis, and the availability of effective treatments have contributed to the declining burden of otitis media in these regions. In contrast, the high burden of otitis media in low and low-middle SDI regions may be linked to several factors, including limited access to healthcare, inadequate vaccination coverage, and poor environmental conditions (24, 25, 28). These factors may explain the persistently high incidence and prevalence of the disease in these regions, despite global efforts to reduce the burden of otitis media.

The findings of this study have significant implications for global public health, particularly in terms of targeting interventions to reduce the burden of otitis media in high-risk regions and populations. Given the persistently high burden of the disease in low and low-middle SDI regions, there is an urgent need for increased investment in healthcare infrastructure, vaccination programs, and public health education in these areas (29). Strategies such as improving access to clean water and sanitation, reducing indoor air pollution, and promoting breastfeeding could also help to reduce the incidence and severity of otitis media (29). In high-income regions, maintaining high vaccination coverage and ensuring access to timely and effective treatment for otitis media will be crucial in sustaining the observed declines in ASPR and ASDR (15, 26, 27). Additionally, ongoing surveillance and research are needed to monitor the long-term effects of vaccination and other public health interventions on the burden of otitis media. The study’s findings also highlight the importance of addressing the social determinants of health, as disparities in otitis media burden are closely linked to socio-economic factors. Efforts to reduce poverty, improve education, and enhance healthcare access in low-resource settings could have a significant impact on reducing the global burden of otitis media (14, 28). These trends underscore the effectiveness of public health interventions, particularly in regions with higher socio-demographic indices. However, persistent disparities in otitis media burden, particularly in low and low-middle SDI regions, highlight the need for continued efforts to address the underlying social determinants of health, improve access to healthcare, and enhance vaccination coverage in these areas.

This study has several strengths that contribute to the robustness and reliability of its findings. First, the use of data from the GBD database, which integrates data from multiple sources and uses advanced modeling techniques, ensures comprehensive and accurate estimates of otitis media burden across different regions and time periods (16). Second, the application of various statistical methods, including joinpoint regression, EAPC calculation, Bayesian age-period-cohort analysis, etc., allows for a nuanced understanding of the trends in otitis media driving these trends. Furthermore, the study’s use of data visualization techniques, such as geographic mapping and temporal trend analysis, enhances the interpretability of the findings and facilitates the identification of key patterns and trends in otitis media burden. These visualizations provide valuable insights that can inform public health strategies and policies aimed at reducing the global burden of otitis media.

Despite its strengths, this study has several limitations that should be acknowledged. One limitation is the potential for biases in self-reported data collection, data reporting, and data gaps, particularly in low-resource settings where healthcare infrastructure and surveillance systems may be less developed. Additionally, projecting trends beyond known variables is challenging due to the uncertainty of future changes. The GBD database relies on complex modeling techniques to estimate disease burden, which may introduce uncertainties in the estimates, particularly in regions with limited data availability. Another limitation is the generalizability of the findings, as the study’s focus on global trends may not fully capture the nuances of otitis media burden in specific populations or regions.

## Conclusion

The study provides a comprehensive analysis of the global burden of otitis media over nearly three decades. The global burden of otitis media shows significant regional disparities, with stable incidence but declining prevalence and DALYs rate. Future research should focus on understanding the factors contributing to the observed disparities and developing targeted interventions to further reduce the burden of otitis media. Additionally, there is a need for ongoing surveillance and more robust data collection in low-resource settings to improve the accuracy of global estimates.

## Data Availability

The raw data supporting the conclusions of this article will be made available by the authors, without undue reservation.

## Footnotes

^1^ https://vizhub.healthdata.org/gbd-results/
