# Supplementary material for: The global burden of otitis media in 204 countries and territories from 1992 to 2021: a systematic analysis for the Global Burden of Disease study 2021
Source: Front Public Health. 2025 Jan 21;12:1519623. doi: 10.3389/fpubh.2024.1519623 (PMC11790571; doi:10.3389/fpubh.2024.1519623)
Supplement: Supplementary file 1 [file Table_1.DOCX]

**The global burden of otitis media in 204 countries and territories** **from 1992 to 2021: a systematic analysis for the Global Burden of Disease Study 2021**

**Supplementary tables**

**Table S1** The cases of incidence and ASIR of otitis media in 1992 and 2021, and its temporal trends from 1992-2021 among 204 countries and territories.

**Table S2** The cases of prevalence and ASPR of otitis media in 1992 and 2021, and its temporal trends from 1992-2021 among 204 countries and territories.

**Table S3** The cases of DALYs and ASDR of otitis media in 1992 and 2021, and its temporal trends from 1992-2021 among 204 countries and territories.

**Table S4** APC model analysis of incidence.

**Table S5** APC model analysis of prevalence.

**Table S6** APC model analysis of DALYs.

**Supplementary figures**

**Figure S1** Global distribution in ASPR and trends in EAPC for both sexes in 204 countries or territories. **A**, the ASPR per 100,000 in 2021. **B**, the EAPC of ASPR from 1992-2021.

**Figure S2** Rates of otitis media on incidence, prevalence, and DALYs from 1992 to 2021. **A**, incidence. **B**, prevalence. **C**, DALYs.

**Figure S3** Cases of otitis media on incidence, prevalence, and DALYs in different SDI. **A**, incidence. **B**, prevalence. **C**, DALYs.

**Figure S4** Joinpoint regression analysis of the sex-specific ASPR for otitis media from 1992 to 2021. **A**, ASPR for males. **B**, ASPR for females.

**Figure S5** Joinpoint regression analysis of the sex-specific ASDR for otitis media from 1992 to 2021. **A**, ASDR for males. **B**, ASDR for females.

**Figure S6** Age, period and cohort effects on incidence of otitis media.

**Figure S7** Age, period and cohort effects on prevalence of otitis media.

**Figure S8** Age, period, and birth cohort effects on DALYs of otitis media.

**Figure S9** Age-specific period and birth cohort effects on incidence, prevalence, and DALYs of otitis media. **A**, period effects on incidence. **B**, birth cohort effects on incidence. **C**, period effects on prevalence. **D**, birth cohort effects on prevalence. **E**, period effects on DALYs. **F**, birth cohort effects on DALYs.

**Figure S10** The age, period, and birth cohort effects on the relative risks (RRs) for incidence, prevalence, and DALYs of otitis media. **A**, incidence. **B**, prevalence. **C**, DALYs.

**Figure S11** Decomposition analysis of incidence, prevalence, and DALYs of otitis media. **A**, incidence stratified by sex. **B**, incidence stratified by SDI levels. **C**, prevalence stratified by sex. **D**, prevalence stratified by SDI levels. **E**, DALYs stratified by sex. **F**, DALYs stratified by SDI levels.

**Figure S12** Frontier analysis of ASIR, ASPR, and ASDR in 2021. **A, B, and C** Frontier analysis for ASIR. **D, E, and F** Frontier analysis for ASPR. **G, H, and I** Frontier analysis for ASDR.

**Figure S13** Health inequality analysis of incidence, prevalence, and DALY rates from 1992 to 2021. **A,** slope index analysis of inequality on incidence. **B**, concentration index analysis on incidence. **C,** slope index analysis of inequality on prevalence. **D**, concentration index analysis on prevalence. **E,** slope index analysis of inequality on DALY rates. **F**, concentration index analysis on DALY rates.

**Figure S14** Autoregressive integrated moving average (ARIMA) model predictions of ASIR, ASPR, and ASDR for males and females. **A**, ASIR for males. **B**, ASIR for females. **C**, ASPR for males. **D**, ASPR for females. **E**, ASDR for males. **F**, ASDR for females.

**Figure S15** The correlation between SDI and global burden of otitis media. **A, C,** and **E** The change trends and correlation of ASIR, ASPR, ASDR and SDI from 1992 to 2021 in 21 regions. **B, D,** and **F** The correlation between ASIR, ASPR, ASDR and SDI of 2021 in 204 countries or territories.

| **Table S1** The cases of incidence and ASIR of otitis media in 1992 and 2021, and its temporal trends from 1992-2021 among 204 countries and territories. | | | | | | |
| --- | --- | --- | --- | --- | --- | --- |
| **Characteristics** | **1992** | |  | **2021** | | **1992-2021** |
|  | **Cases of incidence (95%UI)** | **ASIR per 100,000 (95%UI)** |  | **Cases of incidence (95%UI)** | **ASIR per 100,000 (95%UI)** | **EAPC (95%CI)** |
| Afghanistan | 793806 (575381,1107704) | 5184.66 (3851.99,7119.69) |  | 2407854 (1724762,3397709) | 5176.8 (3846.19,7108.79) | 0 (-0.12 to 0.12) |
| Albania | 149583 (107650,207893) | 4003.17 (2941.82,5472.84) |  | 75879 (59604,99552) | 4004.16 (2944,5472.74) | 0 (-0.06 to 0.06) |
| Algeria | 1803456 (1300815,2536816) | 5179.09 (3845.76,7113.56) |  | 2384809 (1754157,3304110) | 5177.04 (3844.8,7110.83) | 0 (-0.12 to 0.12) |
| American Samoa | 3150 (2286,4372) | 4599.28 (3402.87,6268.41) |  | 2090 (1552,2861) | 4602.35 (3405.87,6272.18) | 0 (-0.12 to 0.12) |
| Andorra | 2149 (1619,2797) | 5302.04 (3917.87,7238.65) |  | 2284 (1786,2926) | 5316.1 (3930.54,7260.46) | 0 (-0.19 to 0.2) |
| Angola | 993368 (717145,1378423) | 5810.91 (4284.64,7931.45) |  | 2859517 (2056475,4008189) | 5816.04 (4289.24,7936.83) | 0 (-0.01 to 0.01) |
| Antigua and Barbuda | 3288 (2418,4481) | 5189 (3844.97,7012.42) |  | 3357 (2590,4424) | 5183.79 (3841.34,6999.78) | 0 (-0.12 to 0.11) |
| Argentina | 1717729 (1270275,2289922) | 4957.46 (3675.76,6573.82) |  | 1779522 (1352149,2346223) | 4955.49 (3673.46,6570.87) | 0 (-0.14 to 0.14) |
| Armenia | 156717 (114981,216346) | 4382.5 (3264.65,5994.38) |  | 102171 (78700,136559) | 4381.23 (3263.4,5992.35) | 0 (-0.07 to 0.06) |
| Australia | 706603 (533605,917120) | 4966.2 (3694.39,6547.86) |  | 899876 (693235,1161996) | 4967.12 (3695.37,6548.29) | 0 (-0.15 to 0.15) |
| Austria | 289796 (223356,385769) | 5319.22 (3929.03,7307.91) |  | 289902 (225882,381333) | 5313.02 (3923.56,7299.8) | 0 (-0.2 to 0.2) |
| Azerbaijan | 372448 (273103,516115) | 4386.27 (3287.21,5968.63) |  | 383706 (293861,514455) | 4380.12 (3281.17,5965.69) | 0 (-0.07 to 0.06) |
| Bahamas | 14547 (10817,19369) | 5183.06 (3858.19,6917.16) |  | 14746 (11390,19219) | 5183.66 (3859.23,6917.75) | 0 (-0.11 to 0.11) |
| Bahrain | 30801 (22682,42560) | 5162.27 (3853.89,7092.23) |  | 58990 (45784,78080) | 5154.71 (3846.37,7082.42) | 0 (-0.12 to 0.11) |
| Bangladesh | 10397100 (7352088,14624268) | 6215.95 (4528.48,8469.08) |  | 9630842 (7031888,13057106) | 6223.39 (4533.86,8478.82) | 0 (-0.11 to 0.11) |
| Barbados | 11321 (8524,15187) | 5193.52 (3839.27,7091.34) |  | 9746 (7601,12650) | 5189.95 (3835.89,7084.38) | 0 (-0.12 to 0.12) |
| Belarus | 390584 (296430,527389) | 4383.57 (3253.16,5970.47) |  | 290294 (224393,384561) | 4379.76 (3249.07,5965.92) | 0 (-0.07 to 0.06) |
| Belgium | 377901 (290904,503197) | 5317.45 (3927.66,7305.78) |  | 394635 (305210,524819) | 5317.95 (3927.9,7306.36) | 0 (-0.2 to 0.2) |
| Belize | 14006 (10235,19303) | 5176.06 (3879.82,6933.37) |  | 21450 (16258,28582) | 5178.73 (3882.06,6935.67) | 0 (-0.12 to 0.12) |
| Benin | 498266 (357234,697124) | 5819.13 (4292.51,7936.21) |  | 1165812 (841310,1628704) | 5815.72 (4289.81,7935.21) | 0 (-0.01 to 0.01) |
| Bermuda | 2453 (1888,3214) | 5187.33 (3889.36,7010.39) |  | 1883 (1501,2398) | 5185.14 (3887.75,7005.23) | 0 (-0.13 to 0.13) |
| Bhutan | 53950 (38890,74906) | 6224.53 (4555.2,8481.01) |  | 41172 (30544,55404) | 6229.64 (4559.06,8486.67) | 0 (-0.11 to 0.11) |
| Bolivia (Plurinational State of) | 473576 (341842,664480) | 5183.24 (3850.01,7118.43) |  | 622323 (462826,855147) | 5180.99 (3848.03,7115.38) | 0 (-0.12 to 0.12) |
| Bosnia and Herzegovina | 156035 (118304,212939) | 3996.86 (2958.85,5519.6) |  | 87609 (69034,114956) | 3996.96 (2958.84,5519.46) | 0 (-0.05 to 0.04) |
| Botswana | 114049 (82338,158761) | 5816.56 (4289.8,7938.13) |  | 139848 (103299,190002) | 5814.62 (4288.01,7933.32) | 0 (-0.01 to 0.01) |
| Brazil | 9488282 (7060697,12944397) | 5677.3 (4252.74,7686.33) |  | 10295727 (7853593,13677733) | 5675.5 (4254.3,7688.3) | 0 (-0.12 to 0.12) |
| Brunei Darussalam | 12917 (9733,17138) | 4005.36 (3067.43,5236.6) |  | 14659 (11427,18722) | 4004.78 (3066.2,5234.21) | 0 (-0.16 to 0.15) |
| Bulgaria | 259843 (200085,343992) | 4007.04 (2948.2,5478.11) |  | 176590 (137361,228092) | 4003.32 (2944.23,5473.78) | 0 (-0.06 to 0.06) |
| Burkina Faso | 943545 (679713,1319390) | 5818.61 (4291.89,7937.32) |  | 2003929 (1447633,2794588) | 5816.69 (4289.61,7937.39) | 0 (-0.01 to 0.01) |
| Burundi | 565553 (403293,786354) | 6119.36 (4472.43,8337.76) |  | 1168249 (831393,1620550) | 6117.02 (4468.49,8335.98) | 0 (-0.09 to 0.09) |
| Cabo Verde | 31180 (22261,43662) | 5813.58 (4290.44,7928.98) |  | 28752 (21507,39153) | 5807.24 (4284.17,7922.61) | 0 (-0.01 to 0.01) |
| Cambodia | 708063 (499436,1008201) | 4461.25 (3244.18,6145.06) |  | 780624 (565984,1078334) | 4455.89 (3241.4,6139.61) | 0 (-0.09 to 0.09) |
| Cameroon | 1014289 (730196,1414675) | 5815.86 (4289.15,7934.73) |  | 2544907 (1837568,3538619) | 5814.22 (4288.08,7932.88) | 0 (-0.01 to 0.01) |
| Canada | 969766 (730480,1271325) | 4385.71 (3250.16,5880.5) |  | 1050809 (818308,1352665) | 4384.76 (3249.5,5879.23) | 0 (-0.03 to 0.03) |
| Central African Republic | 253506 (183052,351899) | 5815.03 (4288.42,7934.01) |  | 435277 (315495,605252) | 5816.31 (4289.7,7935.66) | 0 (-0.01 to 0.01) |
| Chad | 614643 (441395,855197) | 5816.23 (4290.28,7937.19) |  | 1725670 (1237568,2417206) | 5813.42 (4287.12,7933.59) | 0 (-0.01 to 0.01) |
| Chile | 715946 (527015,963938) | 4975.98 (3679.08,6683.14) |  | 672525 (514846,880709) | 4970.13 (3674.57,6677.07) | 0 (-0.15 to 0.15) |
| China | 48448048 (37012823,64736884) | 4183.54 (3199.25,5613.97) |  | 42988078 (33535242,55750275) | 4179.92 (3197.52,5614.57) | 0 (-0.13 to 0.13) |
| Colombia | 2096923 (1555438,2841073) | 5178.3 (3882.26,6934.79) |  | 2034971 (1568243,2659739) | 5176.11 (3881.56,6931.73) | 0 (-0.12 to 0.12) |
| Comoros | 44137 (31523,61190) | 6125.76 (4474.94,8341.39) |  | 49469 (36006,67905) | 6127.37 (4475.56,8342.38) | 0 (-0.09 to 0.09) |
| Congo | 208922 (151704,290627) | 5814.24 (4288.63,7935.02) |  | 364073 (265344,500183) | 5813.61 (4286.53,7933.45) | 0 (-0.01 to 0.01) |
| Cook Islands | 997 (726,1372) | 4590.29 (3420.18,6217.08) |  | 628 (484,840) | 4598.96 (3428.6,6229.27) | 0 (-0.12 to 0.12) |
| Costa Rica | 199772 (145683,274206) | 5179.1 (3847.27,6964.86) |  | 191791 (146028,252627) | 5182.29 (3849.89,6967.06) | 0 (-0.11 to 0.12) |
| Croatia | 147591 (114488,198626) | 4010.45 (2933.11,5568.41) |  | 108592 (85627,142576) | 4007.54 (2930.19,5566.18) | 0 (-0.05 to 0.05) |
| Cuba | 487486 (363032,645622) | 5176.89 (3845.25,6963.01) |  | 372609 (289267,478094) | 5175.52 (3844.35,6961.7) | 0 (-0.12 to 0.11) |
| Cyprus | 37781 (28361,51102) | 5313.76 (3941.72,7263.79) |  | 47954 (37382,63372) | 5317.09 (3943.97,7267.24) | 0 (-0.2 to 0.2) |
| Czechia | 320946 (247780,427508) | 4004.24 (2918.73,5481.63) |  | 294731 (228594,384703) | 3999.58 (2914.91,5476.62) | 0 (-0.06 to 0.05) |
| C么te d'Ivoire | 1176245 (847183,1640309) | 5810.26 (4283.45,7929.37) |  | 2230993 (1616215,3106935) | 5810.87 (4283.86,7927.74) | 0 (-0.01 to 0.01) |
| Democratic People's Republic of Korea | 845984 (640481,1121419) | 3795.28 (2879.8,5084.04) |  | 734460 (574492,928088) | 3788.21 (2872.24,5079.51) | 0 (-0.14 to 0.13) |
| Democratic Republic of the Congo | 3648696 (2631459,5077041) | 5814.25 (4288.6,7933.35) |  | 7150659 (5159660,9941273) | 5813.21 (4287.02,7931.6) | 0 (-0.01 to 0.01) |
| Denmark | 189271 (146485,245147) | 5314.8 (3945.86,7138.84) |  | 200874 (156119,261290) | 5313.03 (3944.22,7136.87) | 0 (-0.2 to 0.2) |
| Djibouti | 41470 (29841,57278) | 6135.03 (4477.06,8342.44) |  | 86388 (62616,117828) | 6140.04 (4479,8339.01) | 0 (-0.09 to 0.09) |
| Dominica | 4219 (3108,5783) | 5172.27 (3845.87,7006.28) |  | 2497 (1932,3298) | 5172.19 (3843.17,7011.61) | 0 (-0.12 to 0.12) |
| Dominican Republic | 480286 (355677,651701) | 5180.92 (3883.56,6938.83) |  | 543319 (409298,722524) | 5175.45 (3880.16,6931.95) | 0 (-0.12 to 0.12) |
| Ecuador | 683968 (499674,952399) | 5182.49 (3849.57,7117.61) |  | 900768 (673212,1232116) | 5181.31 (3848.34,7115.61) | 0 (-0.12 to 0.12) |
| Egypt | 3872708 (2805207,5420073) | 5176.82 (3844.33,7109.87) |  | 6389639 (4652999,8909342) | 5174.93 (3842.86,7107.65) | 0 (-0.12 to 0.12) |
| El Salvador | 369227 (272351,502951) | 5180.31 (3884.43,6935.44) |  | 324304 (243252,434528) | 5179.12 (3883.85,6933.59) | 0 (-0.12 to 0.12) |
| Equatorial Guinea | 42212 (30400,58790) | 5822.47 (4297.92,7940.31) |  | 107806 (77736,148683) | 5814.86 (4292.73,7920.87) | 0 (-0.01 to 0.01) |
| Eritrea | 295372 (210279,409817) | 6136.06 (4481.31,8346.33) |  | 516613 (370804,711625) | 6132.1 (4478.98,8344.73) | 0 (-0.09 to 0.09) |
| Estonia | 56691 (43292,75291) | 4386.18 (3277.07,5946.78) |  | 40151 (31486,51734) | 4378.04 (3269.39,5939.09) | 0 (-0.07 to 0.06) |
| Eswatini | 75517 (54392,105514) | 5818.29 (4290.41,7939.22) |  | 79005 (57427,108409) | 5818.45 (4292.14,7934.53) | 0 (-0.01 to 0.01) |
| Ethiopia | 5728647 (4093309,7999869) | 6640.22 (4857.78,9029.37) |  | 9627660 (6908553,13295607) | 6639.68 (4856.82,9029.7) | 0 (-0.07 to 0.07) |
| Fiji | 41858 (30187,58184) | 4605.63 (3379.4,6320.76) |  | 42910 (31507,59067) | 4605.6 (3378.83,6321.05) | 0 (-0.12 to 0.12) |
| Finland | 196521 (150480,260550) | 5291.76 (3920.82,7206.28) |  | 175085 (137520,228725) | 5286.38 (3916.26,7200.57) | 0 (-0.2 to 0.2) |
| France | 2333659 (1793351,3079915) | 5306.7 (3965.17,7191.97) |  | 2335835 (1808005,3035947) | 5307.65 (3965.85,7193.25) | 0 (-0.19 to 0.19) |
| Gabon | 82456 (59780,114715) | 5812.89 (4286.92,7933.81) |  | 121919 (88894,167502) | 5814.75 (4287.8,7938.25) | 0 (-0.01 to 0.01) |
| Gambia | 95534 (68903,132992) | 5811.12 (4282.8,7932) |  | 188051 (136057,261172) | 5814.83 (4288.39,7935) | 0 (-0.01 to 0.01) |
| Georgia | 213861 (160436,290435) | 4384.29 (3267.02,5996.18) |  | 126659 (96568,170339) | 4376 (3258.33,5987.33) | 0 (-0.07 to 0.06) |
| Germany | 2843013 (2186015,3793640) | 5316.9 (3950.14,7368.25) |  | 2700451 (2111614,3563532) | 5308.88 (3942.51,7359.14) | 0 (-0.2 to 0.19) |
| Ghana | 1339669 (968530,1867808) | 5814.66 (4287.88,7932.91) |  | 2492473 (1807578,3444379) | 5817.18 (4291.55,7935.24) | 0 (-0.01 to 0.01) |
| Greece | 380672 (289732,494044) | 5295.94 (3901.07,7117.43) |  | 305678 (238730,389242) | 5298.96 (3902.75,7121.72) | 0 (-0.21 to 0.21) |
| Greenland | 2401 (1773,3201) | 4364.09 (3223.79,5816.23) |  | 1934 (1476,2517) | 4375.2 (3233.5,5825.76) | 0 (-0.03 to 0.03) |
| Grenada | 5616 (4082,7868) | 5178.48 (3859.03,7101.4) |  | 4160 (3177,5549) | 5170.19 (3853.48,7092.92) | 0 (-0.12 to 0.11) |
| Guam | 7206 (5351,9705) | 4592.69 (3420.13,6223.07) |  | 6188 (4666,8283) | 4593.87 (3422.45,6223.03) | 0 (-0.12 to 0.12) |
| Guatemala | 708640 (509429,979508) | 5180.49 (3883.24,6938.32) |  | 847870 (635589,1144445) | 5180.99 (3884.55,6936.98) | 0 (-0.12 to 0.12) |
| Guinea | 591510 (425386,822604) | 5816.4 (4289.83,7935.59) |  | 1143722 (824558,1598955) | 5817.14 (4291.11,7936.64) | 0 (-0.01 to 0.01) |
| Guinea-Bissau | 95660 (69039,133742) | 5815.92 (4289.28,7936.27) |  | 170642 (123277,238018) | 5817.06 (4291.33,7935.91) | 0 (-0.01 to 0.01) |
| Guyana | 51354 (37913,69501) | 5179.11 (3881.6,6938.45) |  | 38896 (29246,51939) | 5177.13 (3881.48,6934.13) | 0 (-0.12 to 0.12) |
| Haiti | 485789 (353794,665227) | 5183.91 (3884.82,6942.97) |  | 766024 (568991,1035713) | 5180.09 (3883.47,6936.6) | 0 (-0.12 to 0.12) |
| Honduras | 381324 (275596,526561) | 5177.9 (3882.12,6933.5) |  | 566689 (424821,763949) | 5179.22 (3883.63,6934.53) | 0 (-0.12 to 0.12) |
| Hungary | 314818 (246761,417414) | 3996.62 (2952.06,5463.22) |  | 252414 (197868,327616) | 3993.23 (2949.07,5459.54) | 0 (-0.06 to 0.06) |
| Iceland | 12285 (9197,16478) | 5288.42 (3917.63,7201.85) |  | 13445 (10250,17756) | 5284.51 (3914.38,7198.52) | 0 (-0.2 to 0.2) |
| India | 73181676 (52637488,102020275) | 6624.1 (4828,9048.28) |  | 82912015 (61051757,112323205) | 6624.71 (4829.4,9046.91) | 0 (-0.09 to 0.09) |
| Indonesia | 10536047 (7718403,14593203) | 4823.59 (3557.3,6572.13) |  | 11741403 (8770169,15823428) | 4821.53 (3554.8,6569.79) | 0 (-0.08 to 0.08) |
| Iran (Islamic Republic of) | 4484006 (3253161,6270805) | 5671.74 (4269.46,7694.94) |  | 4074209 (3074387,5480014) | 5669.87 (4269.29,7692.03) | 0 (-0.12 to 0.12) |
| Iraq | 1414177 (1016086,1991013) | 5176.5 (3844.11,7110.01) |  | 2286033 (1688246,3153421) | 5175.41 (3843.61,7108.92) | 0 (-0.12 to 0.12) |
| Ireland | 167366 (125293,227569) | 5322.88 (3941.41,7270.52) |  | 191987 (147339,256872) | 5325.88 (3943.71,7274.25) | 0 (-0.2 to 0.2) |
| Israel | 287528 (212967,391943) | 5325.73 (3964.62,7230.52) |  | 490900 (366227,667596) | 5320.62 (3959.86,7223.99) | 0 (-0.2 to 0.2) |
| Italy | 1582266 (1258603,1977987) | 4310.71 (3236.63,5719.34) |  | 1424595 (1134066,1773666) | 4306.4 (3226.7,5697.3) | 0 (-0.21 to 0.21) |
| Jamaica | 142331 (104138,198062) | 5181.29 (3822.82,7112.56) |  | 110070 (84623,145558) | 5178.32 (3820.46,7109.32) | 0 (-0.12 to 0.12) |
| Japan | 3883613 (3099829,4875419) | 4403.26 (3339.56,5749.72) |  | 3146362 (2565354,3876046) | 4399.71 (3334.34,5746.55) | 0 (-0.16 to 0.16) |
| Jordan | 289718 (209261,406997) | 5174.23 (3841.62,7107.91) |  | 621533 (466228,844527) | 5171.76 (3839.67,7105.68) | 0 (-0.12 to 0.12) |
| Kazakhstan | 756158 (553834,1040228) | 4382.26 (3251.82,5969.44) |  | 831013 (615053,1129008) | 4382.11 (3251.12,5968.65) | 0 (-0.06 to 0.06) |
| Kenya | 2519199 (1795711,3516449) | 6635.93 (4854.76,9041.36) |  | 3958651 (2830163,5458940) | 6637.7 (4856.39,9046.74) | 0 (-0.07 to 0.08) |
| Kiribati | 4790 (3429,6693) | 4608.65 (3382.88,6324.2) |  | 6388 (4627,8861) | 4609.04 (3384.25,6324.09) | 0 (-0.12 to 0.12) |
| Kuwait | 91626 (66421,125120) | 5158.72 (3791.63,6995.48) |  | 172304 (131656,224403) | 5166.63 (3797.24,7001.16) | 0 (-0.11 to 0.12) |
| Kyrgyzstan | 247793 (178936,347136) | 4380.66 (3250.12,5967.88) |  | 332408 (241875,460195) | 4380.23 (3249.34,5966.7) | 0 (-0.06 to 0.06) |
| Lao People's Democratic Republic | 280165 (198340,397148) | 4457.33 (3241.68,6141.89) |  | 353584 (256198,489352) | 4454.1 (3239.77,6138.66) | 0 (-0.09 to 0.09) |
| Latvia | 95757 (72990,129313) | 4383.75 (3253.42,5970.86) |  | 56541 (44136,74374) | 4378.85 (3247.75,5965.22) | 0 (-0.07 to 0.06) |
| Lebanon | 187302 (137842,259415) | 5172.83 (3866.02,7102.82) |  | 238108 (181571,320805) | 5169.79 (3864.6,7099.97) | 0 (-0.12 to 0.12) |
| Lesotho | 127360 (92145,177438) | 5825.53 (4299.3,7944.85) |  | 120192 (88118,164588) | 5816.64 (4290.26,7935.15) | 0 (-0.01 to 0.01) |
| Liberia | 172185 (124597,240084) | 5814.76 (4285.82,7936.77) |  | 412241 (298763,571062) | 5811.76 (4285.42,7930.79) | 0 (-0.01 to 0.01) |
| Libya | 296337 (214400,415810) | 5182 (3846.15,7121.32) |  | 276544 (211725,365417) | 5177.49 (3845.06,7111.61) | 0 (-0.12 to 0.12) |
| Lithuania | 138372 (105701,183621) | 4387 (3277.85,5947.78) |  | 80541 (63436,104114) | 4381.84 (3272.98,5943.3) | 0 (-0.07 to 0.06) |
| Luxembourg | 14757 (11273,19027) | 5303.95 (3951.97,7072.98) |  | 21893 (16955,27999) | 5298.68 (3947.18,7066.68) | 0 (-0.2 to 0.2) |
| Madagascar | 1172659 (838238,1626545) | 6122.96 (4472.75,8339.6) |  | 2328719 (1661574,3231545) | 6124 (4473.84,8341.96) | 0 (-0.09 to 0.09) |
| Malawi | 1028515 (737152,1405683) | 6269.4 (4595.13,8405.99) |  | 1649981 (1187254,2229257) | 6382.92 (4664.69,8435.31) | 0.02 (-0.15 to 0.18) |
| Malaysia | 1015115 (728251,1425331) | 4452.83 (3238.75,6137.72) |  | 1223130 (897354,1656430) | 4450.04 (3237.08,6134.75) | 0 (-0.09 to 0.09) |
| Maldives | 15932 (11195,22867) | 4451.71 (3237.33,6139.11) |  | 17356 (12936,23086) | 4437.8 (3228.82,6121.72) | 0 (-0.09 to 0.08) |
| Mali | 840792 (605876,1168822) | 5814.78 (4288.01,7933.99) |  | 2217293 (1594614,3101602) | 5814.1 (4287.46,7933.2) | 0 (-0.01 to 0.01) |
| Malta | 16524 (12385,21943) | 5294.89 (3886.18,7201.13) |  | 14310 (11107,18381) | 5285.12 (3878.69,7191.05) | 0 (-0.21 to 0.2) |
| Marshall Islands | 3154 (2237,4460) | 4604.48 (3376.53,6320.01) |  | 2690 (1980,3704) | 4604.86 (3378.12,6320.41) | 0 (-0.12 to 0.12) |
| Mauritania | 188572 (136069,262774) | 5815.9 (4290.02,7934.93) |  | 347876 (251052,483655) | 5815.54 (4289.59,7935.26) | 0 (-0.01 to 0.01) |
| Mauritius | 49623 (36756,67200) | 4454.69 (3257.81,6065.28) |  | 37648 (29146,49255) | 4454.22 (3257.31,6064.74) | 0 (-0.1 to 0.1) |
| Mexico | 6288529 (4610129,8623299) | 5680.89 (4253.85,7693.85) |  | 6383274 (4834248,8509208) | 5680.67 (4255.79,7693.08) | 0 (-0.12 to 0.12) |
| Micronesia (Federated States of) | 6622 (4728,9313) | 4603.37 (3376.46,6317.12) |  | 4686 (3461,6426) | 4604.85 (3377.88,6319.98) | 0 (-0.12 to 0.12) |
| Monaco | 893 (703,1141) | 5308.11 (3861.15,7310.61) |  | 1135 (883,1466) | 5319.57 (3868.6,7328.32) | 0 (-0.2 to 0.21) |
| Mongolia | 127903 (91929,179204) | 4378.9 (3279.95,5958.64) |  | 161239 (117702,223523) | 4383.65 (3285.29,5965.72) | 0 (-0.06 to 0.06) |
| Montenegro | 22776 (17012,31237) | 4009.02 (2931.83,5566.58) |  | 18356 (14239,24605) | 4008.41 (2930.89,5569.64) | 0 (-0.05 to 0.05) |
| Morocco | 1699460 (1239371,2369312) | 5182.56 (3849.24,7118.11) |  | 1781402 (1338899,2421595) | 5178.89 (3846.51,7112.75) | 0 (-0.12 to 0.12) |
| Mozambique | 1268339 (908088,1757916) | 6126.9 (4475.6,8345.28) |  | 2822834 (2000471,3929441) | 6125.02 (4475.38,8344.35) | 0 (-0.09 to 0.09) |
| Myanmar | 2131345 (1532765,2976127) | 4457.41 (3241.92,6141.61) |  | 2419657 (1763065,3311791) | 4457.19 (3241.87,6140.8) | 0 (-0.09 to 0.09) |
| Namibia | 121754 (88246,169613) | 5814.86 (4288.27,7935.62) |  | 159027 (116271,217932) | 5816 (4289.54,7935.86) | 0 (-0.01 to 0.01) |
| Nauru | 651 (464,911) | 4602.38 (3374.29,6317.39) |  | 605 (437,841) | 4604.14 (3379.46,6318.12) | 0 (-0.12 to 0.12) |
| Nepal | 1828150 (1311420,2560579) | 6230.81 (4562.35,8486.18) |  | 1965541 (1436291,2672594) | 6234.13 (4565.3,8488.51) | 0 (-0.11 to 0.11) |
| Netherlands | 584179 (448672,771047) | 5321.75 (3932.74,7272.69) |  | 574497 (448307,754357) | 5321.18 (3932.3,7271.76) | 0 (-0.2 to 0.2) |
| New Zealand | 164235 (123553,217642) | 5414.14 (4000.29,7314.29) |  | 201894 (154581,264096) | 5409.01 (3997.57,7307.44) | 0 (-0.15 to 0.15) |
| Nicaragua | 310442 (225302,429370) | 5179.83 (3883.4,6936.28) |  | 347942 (260340,467625) | 5176.1 (3881.72,6931.28) | 0 (-0.12 to 0.12) |
| Niger | 834633 (600212,1161232) | 5814.57 (4288.27,7936.06) |  | 2432788 (1744717,3411874) | 5815.46 (4289.93,7934.62) | 0 (-0.01 to 0.01) |
| Nigeria | 8845325 (6351347,12421192) | 6331.43 (4677.36,8627.5) |  | 20794791 (14874395,29228252) | 6342.18 (4681.61,8634.91) | 0 (-0.01 to 0.02) |
| Niue | 112 (82,154) | 4600.87 (3405.89,6271.9) |  | 63 (48,85) | 4601.97 (3405.8,6273.24) | 0 (-0.12 to 0.12) |
| North Macedonia | 72926 (53385,99618) | 4003.85 (2903.18,5530.19) |  | 58055 (45236,75947) | 3998.87 (2898.58,5525.47) | 0 (-0.06 to 0.05) |
| Northern Mariana Islands | 2318 (1698,3137) | 4599.8 (3367.38,6265.84) |  | 1798 (1372,2412) | 4594.03 (3365.33,6254.17) | 0 (-0.13 to 0.12) |
| Norway | 186081 (142164,243890) | 5795.58 (4282.97,7804.37) |  | 206781 (159266,268871) | 5791 (4278.9,7799.98) | 0 (-0.21 to 0.21) |
| Oman | 150435 (108300,211255) | 5164.55 (3831.9,7099.89) |  | 228202 (168434,312148) | 5164.08 (3831.92,7099.92) | 0 (-0.12 to 0.12) |
| Pakistan | 11347718 (8096646,15795037) | 6764.88 (4936.43,9215.7) |  | 19193447 (13748099,26461394) | 6768 (4938.04,9218.64) | 0 (-0.11 to 0.12) |
| Palau | 734 (540,991) | 4604.24 (3389.56,6232.86) |  | 575 (444,760) | 4592.81 (3380.34,6224.27) | 0 (-0.12 to 0.12) |
| Palestine | 179148 (128225,253192) | 5180.14 (3847.79,7112.79) |  | 311858 (227518,433900) | 5178.21 (3845.88,7112.22) | 0 (-0.12 to 0.12) |
| Panama | 146015 (108849,198077) | 5174.19 (3879.66,6930.88) |  | 206074 (154604,275488) | 5172.84 (3879.21,6929.33) | 0 (-0.12 to 0.12) |
| Papua New Guinea | 271218 (194062,379859) | 4600.37 (3373.37,6313.33) |  | 611266 (439720,849629) | 4600.05 (3373.66,6313.27) | 0 (-0.12 to 0.12) |
| Paraguay | 295457 (212763,415128) | 5179.8 (3847.3,7114.69) |  | 356458 (267786,486224) | 5177.72 (3845.65,7111.66) | 0 (-0.12 to 0.12) |
| Peru | 1432962 (1047738,1994037) | 5182.66 (3849.32,7118.1) |  | 1761031 (1319806,2401268) | 5176.3 (3844.68,7107.83) | 0 (-0.12 to 0.12) |
| Philippines | 4109898 (2964310,5686657) | 4822.23 (3556.45,6570.09) |  | 5552873 (4092327,7607275) | 4820.53 (3555.68,6568.34) | 0 (-0.08 to 0.08) |
| Poland | 1281293 (1039271,1569576) | 3858.11 (3087.64,4775.39) |  | 970088 (823239,1138587) | 3767.26 (3089.27,4564.42) | -0.02 (-0.07 to 0.04) |
| Portugal | 380878 (295891,500081) | 5319.8 (3889.54,7322.24) |  | 310798 (244199,400883) | 5319.37 (3888.87,7323.65) | 0 (-0.2 to 0.2) |
| Puerto Rico | 176347 (134854,239542) | 5186.94 (3900.04,7096.49) |  | 93533 (75038,119854) | 5185.72 (3899.52,7094.75) | 0 (-0.13 to 0.13) |
| Qatar | 25249 (18503,34144) | 5138.15 (3808.83,6936.81) |  | 109256 (82975,142239) | 5140.43 (3809.87,6941.95) | 0 (-0.12 to 0.11) |
| Republic of Korea | 1318271 (678273,1730108) | 3296.29 (1547.71,4411.16) |  | 1136985 (909879,1407302) | 4013.07 (3074.09,5246.48) | 0.51 (0.3 to 0.72) |
| Republic of Moldova | 186902 (140090,254498) | 4387.32 (3289.17,5968.76) |  | 102732 (81356,133293) | 4381.34 (3283.06,5963.76) | 0 (-0.07 to 0.06) |
| Romania | 780516 (596480,1048302) | 4006.84 (2948.22,5478.18) |  | 517622 (402227,675590) | 4003.85 (2944.92,5474.47) | 0 (-0.06 to 0.06) |
| Russian Federation | 6097927 (4631282,8254730) | 4766.46 (3575.79,6490.54) |  | 5091675 (3966163,6725294) | 4765.67 (3574.77,6488.43) | 0 (-0.07 to 0.07) |
| Rwanda | 714680 (508880,990688) | 6120.86 (4473.68,8341.3) |  | 1006645 (726533,1388810) | 6127.45 (4477.33,8345.37) | 0 (-0.09 to 0.09) |
| Saint Kitts and Nevis | 2360 (1735,3260) | 5176.62 (3846.4,7016.95) |  | 2050 (1595,2636) | 5175.07 (3845.35,7015.9) | 0 (-0.12 to 0.12) |
| Saint Lucia | 8649 (6287,11920) | 5184.94 (3843.27,7009.04) |  | 6097 (4739,7966) | 5177.82 (3837.69,6996.19) | 0 (-0.12 to 0.11) |
| Saint Vincent and the Grenadines | 6547 (4826,9103) | 5175.85 (3857.32,7099.5) |  | 4593 (3509,6156) | 5172.97 (3854.61,7095.6) | 0 (-0.12 to 0.12) |
| Samoa | 10480 (7548,14598) | 4599.06 (3384.77,6234.29) |  | 12195 (8829,16849) | 4600.67 (3387.38,6230.92) | 0 (-0.12 to 0.12) |
| San Marino | 842 (660,1100) | 5324.96 (3963.28,7229.39) |  | 953 (754,1232) | 5323.54 (3962.12,7226.01) | 0 (-0.2 to 0.2) |
| Sao Tome and Principe | 10868 (7784,15154) | 5809.76 (4286.42,7924.73) |  | 14524 (10586,20003) | 5807.63 (4284.52,7923.06) | 0 (-0.01 to 0.01) |
| Saudi Arabia | 1166841 (839639,1637468) | 5168.37 (3835.16,7103.27) |  | 1501571 (1142867,1999654) | 5162.13 (3831.75,7093.65) | 0 (-0.12 to 0.12) |
| Senegal | 731214 (526973,1021729) | 5813.91 (4287.2,7935.88) |  | 1206801 (873083,1673929) | 5814.48 (4288.92,7930.78) | 0 (-0.01 to 0.01) |
| Serbia | 316781 (239204,427036) | 3993.41 (2890.12,5517.45) |  | 230828 (180643,301916) | 3999.37 (2897.96,5519.84) | 0 (-0.05 to 0.06) |
| Seychelles | 3524 (2552,4890) | 4454.15 (3240.26,6137.56) |  | 3878 (2862,5206) | 4449.53 (3237.4,6133.04) | 0 (-0.09 to 0.09) |
| Sierra Leone | 365747 (263283,506777) | 5814.71 (4286.84,7935.44) |  | 692517 (502804,962072) | 5812.75 (4285.67,7933.44) | 0 (-0.01 to 0.01) |
| Singapore | 107580 (83272,137521) | 4012.61 (3019.08,5229.88) |  | 151161 (118124,193826) | 4011.36 (3019.38,5226.93) | 0 (-0.16 to 0.16) |
| Slovakia | 185112 (139746,252780) | 4011.59 (2934.36,5568.52) |  | 149773 (116572,197839) | 4006.95 (2929.61,5564.92) | 0 (-0.05 to 0.05) |
| Slovenia | 60802 (46848,81187) | 4004.51 (2918.85,5481.83) |  | 55122 (43186,71965) | 3997.89 (2913.32,5474.6) | 0 (-0.06 to 0.05) |
| Solomon Islands | 24339 (17428,34296) | 4600.63 (3373.42,6314.29) |  | 39616 (28481,55177) | 4602.8 (3376.89,6316.58) | 0 (-0.12 to 0.12) |
| Somalia | 754441 (539500,1046542) | 6141.51 (4482.51,8346.19) |  | 2110129 (1506288,2930317) | 6134.46 (4480.29,8345.92) | 0 (-0.09 to 0.09) |
| South Africa | 2931010 (2149747,4051928) | 6336.08 (4690.15,8641.36) |  | 3357045 (2495965,4524608) | 6335.66 (4684.71,8634.99) | 0 (-0.01 to 0.02) |
| South Sudan | 556029 (398508,768831) | 6130.23 (4473.14,8338.29) |  | 848946 (612115,1176510) | 6135.14 (4478.4,8343.44) | 0 (-0.09 to 0.09) |
| Spain | 1760209 (1443758,2215122) | 6705.22 (5335.68,8587.79) |  | 1729521 (1411903,2152976) | 6702.59 (5333.1,8584.72) | 0 (-0.2 to 0.2) |
| Sri Lanka | 806111 (583373,1113701) | 4455.01 (3240.09,6139.58) |  | 815056 (605070,1096497) | 4456.87 (3241.61,6140.83) | 0 (-0.09 to 0.09) |
| Sudan | 1643848 (1188693,2305989) | 5175.74 (3844.63,7110.91) |  | 2773150 (2017309,3865971) | 5176.26 (3844.44,7109.68) | 0 (-0.12 to 0.12) |
| Suriname | 22055 (16484,29833) | 5179.57 (3882.6,6936.46) |  | 25918 (19706,34212) | 5175.73 (3881.25,6930.04) | 0 (-0.12 to 0.12) |
| Sweden | 372147 (282702,489113) | 5762.48 (4265.03,7800.43) |  | 404998 (311375,529201) | 5757.84 (4265.54,7787.34) | 0 (-0.2 to 0.2) |
| Switzerland | 256283 (197741,332718) | 5314.17 (3945.28,7138.24) |  | 293454 (228609,379900) | 5311 (3942.83,7134.41) | 0 (-0.2 to 0.19) |
| Syrian Arab Republic | 1017871 (730219,1434977) | 5177.24 (3845.01,7111.3) |  | 627035 (478780,833256) | 5185.87 (3853.43,7121.43) | 0 (-0.12 to 0.12) |
| Taiwan (Province of China) | 607950 (457543,805703) | 3219.15 (2403.89,4328.55) |  | 481684 (378905,627775) | 3220.69 (2405.95,4330.49) | 0 (-0.13 to 0.13) |
| Tajikistan | 342031 (245901,483526) | 4380.01 (3281.04,5960.62) |  | 521373 (380109,723395) | 4378.74 (3279.87,5960.29) | 0 (-0.06 to 0.06) |
| Thailand | 2356404 (1912207,2908365) | 4229.68 (3434.35,5185.26) |  | 1824865 (1413962,2374710) | 4454.81 (3240.11,6139.51) | 0.03 (-0.07 to 0.14) |
| Timor-Leste | 52560 (37216,74056) | 4450.5 (3237.26,6137.49) |  | 75779 (54392,106071) | 4452.35 (3238.18,6138.52) | 0 (-0.09 to 0.09) |
| Togo | 351340 (253042,491505) | 5815.7 (4289.66,7935.1) |  | 629684 (455857,872157) | 5816.87 (4290.48,7933.67) | 0 (-0.01 to 0.01) |
| Tokelau | 86 (61,120) | 4606.79 (3382.94,6320.71) |  | 57 (42,79) | 4600.96 (3374.72,6314.82) | 0 (-0.12 to 0.12) |
| Tonga | 6135 (4425,8539) | 4603 (3388.93,6234.35) |  | 5993 (4346,8272) | 4602.46 (3389.51,6233.25) | 0 (-0.12 to 0.12) |
| Trinidad and Tobago | 66866 (48805,92625) | 5179 (3820.61,7110) |  | 52485 (40410,69514) | 5176.3 (3818.89,7107.41) | 0 (-0.12 to 0.12) |
| Tunisia | 530031 (387329,737036) | 5178.61 (3846.16,7112.94) |  | 516161 (390175,697394) | 5176.17 (3845.13,7108.58) | 0 (-0.12 to 0.12) |
| Turkey | 3486834 (2580306,4808109) | 5178.36 (3846.22,7111.76) |  | 3446804 (2625297,4621428) | 5178.35 (3845.92,7112.08) | 0 (-0.12 to 0.12) |
| Turkmenistan | 219442 (158161,307850) | 4382.44 (3283.52,5963.6) |  | 230886 (172017,315401) | 4372.83 (3274.35,5954.8) | 0 (-0.07 to 0.06) |
| Tuvalu | 560 (403,778) | 4604.49 (3384.05,6315.19) |  | 587 (430,808) | 4598.45 (3372.03,6311.3) | 0 (-0.12 to 0.12) |
| Uganda | 1875172 (1339656,2614816) | 6121.93 (4472.52,8339.57) |  | 3942444 (2805476,5484902) | 6131.84 (4478.96,8345.12) | 0 (-0.09 to 0.09) |
| Ukraine | 2049813 (1601355,2744663) | 4778.7 (3604.74,6566.33) |  | 1335796 (1055249,1712500) | 4774.91 (3601.34,6560.83) | 0 (-0.07 to 0.07) |
| United Arab Emirates | 115466 (84797,159320) | 5148.08 (3841.22,7076.51) |  | 308403 (237683,403388) | 5148.68 (3839.4,7078.71) | 0 (-0.12 to 0.12) |
| United Kingdom | 2473191 (1888650,3295445) | 5709.86 (4256.87,7859.21) |  | 2597467 (1994501,3408400) | 5721.19 (4264.9,7871.17) | 0.01 (-0.2 to 0.21) |
| United Republic of Tanzania | 2591153 (1856739,3599881) | 6126.06 (4474.8,8343.45) |  | 4892549 (3496370,6777616) | 6124.74 (4474.36,8341.92) | 0 (-0.09 to 0.09) |
| United States of America | 8665132 (7456537,10055376) | 4037.65 (3446.66,4728.65) |  | 9297294 (8148939,10611731) | 4168.22 (3571.78,4848.98) | 0.05 (0.03 to 0.06) |
| United States Virgin Islands | 5634 (4163,7637) | 5192.24 (3829.8,7044.68) |  | 2760 (2171,3575) | 5183.57 (3825.91,7029.45) | 0 (-0.12 to 0.12) |
| Uruguay | 142512 (106795,187641) | 4956.6 (3674.21,6572.38) |  | 120784 (93101,157201) | 4956.19 (3673.81,6570.86) | 0 (-0.14 to 0.14) |
| Uzbekistan | 1257864 (905217,1767984) | 4382.19 (3283.23,5962.98) |  | 1555549 (1159227,2121909) | 4381.58 (3282.82,5964.03) | 0 (-0.06 to 0.06) |
| Vanuatu | 10743 (7662,15105) | 4602.32 (3374.75,6315.82) |  | 17828 (12782,24842) | 4605.38 (3379.16,6320.1) | 0 (-0.12 to 0.12) |
| Venezuela (Bolivarian Republic of) | 1252972 (915825,1741638) | 5175.24 (3857.24,7099.03) |  | 1218655 (918306,1649412) | 5176.92 (3859.25,7100.65) | 0 (-0.12 to 0.12) |
| Viet Nam | 3918749 (2794897,5544749) | 4457.45 (3241.95,6141.25) |  | 3971100 (2896345,5420798) | 4451.64 (3238.42,6136.29) | 0 (-0.09 to 0.09) |
| Yemen | 1245814 (893128,1760794) | 5177.08 (3845.18,7109.4) |  | 2270693 (1641534,3181398) | 5179.03 (3846.78,7112.69) | 0 (-0.12 to 0.12) |
| Zambia | 798327 (573396,1112339) | 6119.35 (4470.68,8338.18) |  | 1641001 (1167888,2277849) | 6121.82 (4472.26,8339.3) | 0 (-0.09 to 0.09) |
| Zimbabwe | 917847 (662025,1280257) | 5814 (4286.84,7934.96) |  | 1187755 (859873,1645207) | 5817.6 (4290.56,7937.25) | 0 (-0.01 to 0.01) |
| ASIR, age-standardized incidence rate. EAPC, estimated annual percentage changes. | | | | | | |

| **Table S2** The cases of prevalence and ASPR of otitis media in 1992 and 2021, and its temporal trends from 1992-2021 among 204 countries and territories. | | | | | | |
| --- | --- | --- | --- | --- | --- | --- |
| **Characteristics** | **1992** | |  | **2021** | | **1992-2021** |
|  | **Cases of prevalence (95%UI)** | **ASPR per 100,000 (95%UI)** |  | **Cases of prevalence (95%UI)** | **ASPR per 100,000 (95%UI)** | **EAPC (95%CI)** |
| Afghanistan | 231759 (201530,268426) | 2074.21 (1740.94,2424.02) |  | 706160 (607252,825379) | 2049.88 (1729.33,2393.61) | -0.29 (-0.44 to -0.14) |
| Albania | 56486 (48067,66437) | 1635.06 (1390.11,1909.05) |  | 32361 (27167,38343) | 1346.31 (1149.54,1572.46) | -0.83 (-0.95 to -0.71) |
| Algeria | 456142 (394533,531023) | 1529.7 (1311.85,1770.38) |  | 654854 (563490,761198) | 1460.82 (1259.33,1697.49) | -0.27 (-0.38 to -0.16) |
| American Samoa | 708 (608,822) | 1213.7 (1048.38,1421.64) |  | 630 (536,737) | 1265.66 (1086.26,1467.51) | 0.12 (0.01 to 0.22) |
| Andorra | 517 (429,611) | 989.55 (846.32,1151.05) |  | 635 (522,757) | 941.91 (804.02,1097.49) | -0.19 (-0.21 to -0.16) |
| Angola | 221210 (190919,257756) | 1762.38 (1492.79,2049.79) |  | 592186 (510270,688052) | 1542.89 (1325.91,1787.89) | -0.67 (-0.79 to -0.55) |
| Antigua and Barbuda | 863 (743,1004) | 1372.95 (1183.77,1580.68) |  | 1029 (870,1217) | 1292.44 (1111.09,1493.24) | -0.2 (-0.28 to -0.11) |
| Argentina | 405247 (348680,465982) | 1181.76 (1016.35,1365.67) |  | 456894 (390150,537020) | 1092.88 (942.54,1266.25) | -0.31 (-0.34 to -0.27) |
| Armenia | 56331 (48225,65766) | 1617.55 (1381.39,1880.92) |  | 38537 (32441,45378) | 1408.36 (1203.65,1636.92) | -0.77 (-0.91 to -0.64) |
| Australia | 132159 (113308,154047) | 850.18 (730.99,986.89) |  | 166912 (143746,192437) | 793.21 (684.78,919.06) | -0.26 (-0.37 to -0.15) |
| Austria | 73181 (61189,86564) | 1045.94 (884.99,1221.24) |  | 71643 (59565,85175) | 940.02 (801.66,1104.57) | -0.36 (-0.39 to -0.33) |
| Azerbaijan | 114193 (97465,134405) | 1444.84 (1233.1,1685.69) |  | 127947 (109153,150855) | 1288.93 (1108.42,1502.46) | -0.83 (-1.01 to -0.65) |
| Bahamas | 3444 (2970,4008) | 1250.73 (1087.89,1446.81) |  | 4376 (3728,5134) | 1244.23 (1073.14,1437.85) | -0.04 (-0.12 to 0.05) |
| Bahrain | 7249 (6191,8514) | 1300.15 (1120.5,1514.43) |  | 16930 (14155,20324) | 1230.59 (1057.53,1441.89) | -0.24 (-0.34 to -0.13) |
| Bangladesh | 3314394 (2849792,3905984) | 2637.14 (2234.81,3133.22) |  | 3570877 (3029501,4197006) | 2154.9 (1844.67,2504.12) | -0.75 (-0.83 to -0.67) |
| Barbados | 3278 (2813,3834) | 1373.52 (1186.4,1583.8) |  | 3544 (3016,4166) | 1383.83 (1192.87,1603.57) | 0.06 (-0.03 to 0.15) |
| Belarus | 145228 (124326,168938) | 1471.76 (1266.38,1706.46) |  | 107435 (91643,125427) | 1344.67 (1165.09,1552.15) | -0.51 (-0.64 to -0.39) |
| Belgium | 99148 (83400,116531) | 1118.72 (955.52,1298.92) |  | 99415 (84142,116163) | 1024.49 (867.47,1197.57) | -0.31 (-0.33 to -0.28) |
| Belize | 3548 (3092,4092) | 1621.2 (1396.85,1882.29) |  | 6580 (5619,7695) | 1517.19 (1303.47,1752.7) | -0.21 (-0.3 to -0.12) |
| Benin | 124287 (106004,145505) | 2169.09 (1820.87,2543.81) |  | 300609 (257255,352142) | 2006.68 (1703.59,2355.48) | -0.32 (-0.44 to -0.19) |
| Bermuda | 646 (550,764) | 1207.05 (1040,1404.54) |  | 549 (467,648) | 1121.86 (967.77,1300.38) | -0.31 (-0.39 to -0.23) |
| Bhutan | 16602 (14088,19625) | 2293.48 (1946.81,2664.98) |  | 13602 (11506,15988) | 1847.01 (1584.19,2137.67) | -0.78 (-0.85 to -0.7) |
| Bolivia (Plurinational State of) | 126224 (109902,145943) | 1737.52 (1494.82,2022.26) |  | 184096 (157643,213510) | 1540.76 (1322.49,1774.84) | -0.46 (-0.55 to -0.38) |
| Bosnia and Herzegovina | 84713 (70808,101039) | 1892.63 (1594.69,2229.88) |  | 38974 (32709,46299) | 1343.23 (1145.83,1575.56) | -1.28 (-1.43 to -1.13) |
| Botswana | 26827 (23189,31016) | 1638.95 (1421.57,1896.2) |  | 34940 (30131,40678) | 1438.1 (1241.07,1669.44) | -0.46 (-0.58 to -0.34) |
| Brazil | 2441142 (2120421,2844263) | 1518.04 (1321.03,1758.42) |  | 2919799 (2524475,3414294) | 1424.96 (1241.36,1658.3) | -0.23 (-0.3 to -0.16) |
| Brunei Darussalam | 2574 (2200,2988) | 872.26 (752.82,1012.16) |  | 3599 (3040,4264) | 876.74 (755.24,1025.27) | -0.05 (-0.08 to -0.02) |
| Bulgaria | 107392 (90704,126365) | 1363.89 (1160.3,1596.74) |  | 70888 (59325,82878) | 1231.33 (1056,1428.84) | -0.49 (-0.62 to -0.36) |
| Burkina Faso | 251865 (216804,293647) | 2337.7 (1963.3,2759.86) |  | 530709 (455185,623233) | 2129.23 (1801.71,2515.36) | -0.36 (-0.48 to -0.23) |
| Burundi | 131326 (112883,153755) | 1999.51 (1694.63,2330.37) |  | 312974 (267676,366912) | 2156.93 (1824.41,2509.8) | 0.23 (0.11 to 0.35) |
| Cabo Verde | 8355 (7145,9747) | 2055.51 (1744.26,2410.49) |  | 9289 (7811,11041) | 1682.24 (1430.06,1968.19) | -0.78 (-0.91 to -0.65) |
| Cambodia | 236934 (203654,277188) | 2001.28 (1677.1,2337.54) |  | 279178 (235829,331132) | 1609.91 (1365.42,1889.62) | -0.85 (-0.98 to -0.73) |
| Cameroon | 250910 (215986,291557) | 1989.28 (1668.81,2315.85) |  | 684620 (589216,798704) | 1938.84 (1641.18,2258.12) | -0.2 (-0.33 to -0.07) |
| Canada | 245866 (210474,286517) | 978.57 (836.27,1128.1) |  | 282438 (241797,330213) | 921.95 (795.89,1062.84) | -0.23 (-0.28 to -0.17) |
| Central African Republic | 66935 (57368,78228) | 2130.1 (1796.86,2489.89) |  | 134270 (114337,158367) | 2290.43 (1920.94,2703.44) | 0.22 (0.11 to 0.33) |
| Chad | 159897 (136299,187491) | 2307.73 (1931.02,2723.23) |  | 415139 (356789,481631) | 2082.74 (1757.37,2459.82) | -0.5 (-0.63 to -0.37) |
| Chile | 173002 (148722,201178) | 1238.8 (1068.1,1435.92) |  | 174026 (148467,203761) | 1053.9 (910.25,1214.04) | -0.6 (-0.62 to -0.57) |
| China | 22497779 (18985083,26552083) | 1835.2 (1574.08,2131.42) |  | 16730890 (14468523,19600015) | 1332.11 (1158.46,1541.88) | -1.18 (-1.3 to -1.06) |
| Colombia | 570406 (497124,658234) | 1569.39 (1362.24,1815.65) |  | 655881 (565335,764550) | 1438.16 (1247.85,1652.49) | -0.34 (-0.43 to -0.25) |
| Comoros | 9446 (8069,11098) | 1694.64 (1444.18,1972.6) |  | 13335 (11315,15666) | 1720.68 (1453.31,2010.49) | 0.04 (-0.08 to 0.16) |
| Congo | 48344 (41506,56167) | 1684.96 (1433.99,1959.92) |  | 92600 (79210,109472) | 1603.37 (1370.52,1880.68) | -0.3 (-0.42 to -0.19) |
| Cook Islands | 258 (220,299) | 1253.6 (1081.47,1459.32) |  | 177 (151,206) | 1104.51 (948.51,1291.4) | -0.39 (-0.49 to -0.3) |
| Costa Rica | 53820 (46780,62159) | 1551.7 (1348.15,1803.02) |  | 61525 (52947,72056) | 1408.68 (1220.79,1639.97) | -0.36 (-0.45 to -0.28) |
| Croatia | 58345 (49251,68363) | 1311.77 (1122,1519.84) |  | 42439 (35633,49471) | 1194.94 (1015.73,1390.82) | -0.44 (-0.55 to -0.32) |
| Cuba | 167760 (142858,197139) | 1581.78 (1367.13,1834.76) |  | 150829 (128069,178634) | 1519.19 (1300.04,1763.14) | -0.3 (-0.4 to -0.2) |
| Cyprus | 8984 (7630,10512) | 1162.24 (984.94,1354.22) |  | 12387 (10371,14688) | 1058.66 (899.45,1227.79) | -0.32 (-0.35 to -0.28) |
| Czechia | 116029 (98302,136419) | 1225.56 (1045.99,1433.88) |  | 101991 (86275,121005) | 1125.33 (962.84,1325.41) | -0.36 (-0.47 to -0.24) |
| C么te d'Ivoire | 288871 (248992,338083) | 1944.1 (1641.8,2270.6) |  | 572989 (488223,670066) | 1862.64 (1574.35,2182.82) | -0.12 (-0.25 to 0.01) |
| Democratic People's Republic of Korea | 361861 (306290,422506) | 1679.58 (1428.25,1966.17) |  | 450187 (372121,539941) | 1776.59 (1492.33,2082.81) | 0.13 (0.01 to 0.26) |
| Democratic Republic of the Congo | 901914 (772983,1051585) | 2012.04 (1701.51,2346.88) |  | 2161711 (1841071,2542420) | 2235.34 (1882.69,2627.6) | 0.26 (0.11 to 0.42) |
| Denmark | 49778 (41850,58908) | 1096.47 (923.4,1280.04) |  | 49181 (41430,57204) | 996.39 (845.23,1158.79) | -0.33 (-0.35 to -0.3) |
| Djibouti | 9084 (7722,10758) | 1625.83 (1375.19,1891.71) |  | 21259 (18109,24896) | 1606.34 (1371.56,1875.48) | -0.06 (-0.18 to 0.07) |
| Dominica | 1159 (1006,1340) | 1547.94 (1334.59,1791.95) |  | 909 (770,1068) | 1469.01 (1262.57,1706.79) | -0.2 (-0.29 to -0.11) |
| Dominican Republic | 128476 (111625,148414) | 1603.37 (1384.51,1850.73) |  | 148981 (128238,173674) | 1373.47 (1189.13,1588.42) | -0.57 (-0.65 to -0.48) |
| Ecuador | 175961 (152841,202908) | 1547.45 (1341.69,1786.17) |  | 259842 (225425,302912) | 1444.61 (1255.12,1677.27) | -0.34 (-0.43 to -0.25) |
| Egypt | 1108858 (950731,1279920) | 1760.35 (1502.82,2041.81) |  | 1723067 (1485225,2005171) | 1524.73 (1313.75,1769.11) | -0.54 (-0.65 to -0.43) |
| El Salvador | 103136 (89980,118620) | 1742.32 (1504.97,2025.28) |  | 100928 (86769,117152) | 1565.65 (1347.04,1817.74) | -0.39 (-0.48 to -0.3) |
| Equatorial Guinea | 10036 (8642,11760) | 1948.07 (1632.94,2280.41) |  | 21615 (18419,25501) | 1264.71 (1083.92,1473.7) | -1.87 (-2.1 to -1.63) |
| Eritrea | 70195 (59990,82111) | 1957.27 (1655.95,2288.32) |  | 131750 (112893,155609) | 1830.31 (1553.47,2147.68) | -0.09 (-0.22 to 0.04) |
| Estonia | 20912 (17937,24378) | 1451.87 (1247,1687.04) |  | 13559 (11648,15867) | 1246.21 (1083.44,1445.42) | -0.64 (-0.76 to -0.53) |
| Eswatini | 17270 (14978,19897) | 1690.12 (1464.43,1958.91) |  | 19852 (16963,23065) | 1574.59 (1350.82,1825) | -0.23 (-0.35 to -0.11) |
| Ethiopia | 1403297 (1218627,1638155) | 2282.51 (1942.27,2648.07) |  | 2356072 (2026138,2759460) | 1931.58 (1665.72,2238.22) | -0.68 (-0.81 to -0.56) |
| Fiji | 12317 (10475,14351) | 1481.48 (1271.9,1716.06) |  | 12629 (10814,14739) | 1348.97 (1157.07,1567.48) | -0.29 (-0.39 to -0.18) |
| Finland | 51970 (43812,61791) | 1124.27 (959.45,1327.49) |  | 45262 (38367,53011) | 995.21 (843.85,1153.58) | -0.38 (-0.41 to -0.34) |
| France | 607289 (515545,708648) | 1162.9 (991.37,1351.98) |  | 602189 (509622,703049) | 1072.71 (918.71,1240.86) | -0.29 (-0.34 to -0.25) |
| Gabon | 16063 (13733,18753) | 1355.76 (1165.79,1582.36) |  | 26382 (22663,31053) | 1344.3 (1151.7,1570.52) | -0.05 (-0.17 to 0.06) |
| Gambia | 24524 (20943,28828) | 2077.52 (1752.9,2432.06) |  | 54567 (46369,64155) | 2094.95 (1760.36,2448.46) | 0.02 (-0.11 to 0.14) |
| Georgia | 76030 (65285,88990) | 1440 (1233.62,1686) |  | 45574 (38678,53271) | 1380.87 (1188.02,1610.96) | -0.49 (-0.65 to -0.32) |
| Germany | 770945 (645902,912058) | 1090.32 (926.16,1267.55) |  | 693723 (581218,819198) | 988.92 (847.3,1153.2) | -0.34 (-0.36 to -0.32) |
| Ghana | 354265 (305679,414054) | 2045.54 (1714.89,2389.18) |  | 659018 (562293,769738) | 1771.19 (1507.59,2064.72) | -0.53 (-0.65 to -0.4) |
| Greece | 113132 (95201,132581) | 1197.67 (1015.01,1385.15) |  | 94294 (78848,112032) | 1117.97 (947.32,1297.36) | -0.26 (-0.3 to -0.22) |
| Greenland | 504 (432,582) | 926.36 (802.46,1065.92) |  | 424 (364,490) | 852.31 (735.07,982.86) | -0.36 (-0.42 to -0.3) |
| Grenada | 1499 (1303,1725) | 1586.59 (1366.61,1835.02) |  | 1342 (1148,1565) | 1400.25 (1208.68,1611.52) | -0.44 (-0.53 to -0.35) |
| Guam | 1579 (1342,1833) | 1070.19 (917.39,1250.91) |  | 1514 (1293,1765) | 1033.13 (880.15,1206.48) | -0.16 (-0.25 to -0.06) |
| Guatemala | 173511 (151358,198444) | 1693.97 (1469.38,1957.79) |  | 257035 (222306,297427) | 1573 (1354.41,1821.7) | -0.28 (-0.38 to -0.19) |
| Guinea | 149783 (129128,174714) | 2120.43 (1794.31,2483.2) |  | 302761 (261134,354019) | 2049.83 (1732.52,2390.89) | -0.07 (-0.2 to 0.06) |
| Guinea-Bissau | 25372 (21800,29894) | 2200.26 (1859.21,2588) |  | 48453 (40986,56848) | 2150.86 (1800.22,2527.02) | -0.03 (-0.16 to 0.09) |
| Guyana | 14550 (12588,16914) | 1748.12 (1508.79,2030.4) |  | 11499 (9895,13438) | 1497.62 (1298.47,1739.26) | -0.47 (-0.56 to -0.38) |
| Haiti | 139383 (121050,161061) | 1933.61 (1648.81,2246.75) |  | 264535 (223689,309737) | 1982.73 (1684.37,2308.43) | 0.06 (-0.03 to 0.16) |
| Honduras | 100865 (87880,115897) | 1797.91 (1539.67,2085.47) |  | 178480 (153180,207334) | 1694.04 (1460.18,1951.27) | -0.22 (-0.31 to -0.13) |
| Hungary | 125140 (105601,147456) | 1315.06 (1117.73,1530.7) |  | 95930 (80608,113449) | 1179.02 (1001.09,1370.4) | -0.43 (-0.55 to -0.31) |
| Iceland | 2706 (2303,3165) | 1094.42 (930.32,1278.24) |  | 3081 (2613,3611) | 1001.39 (853.08,1164.49) | -0.33 (-0.35 to -0.31) |
| India | 23291424 (19966645,27165702) | 2409 (2058.42,2784.19) |  | 28343124 (24063462,33028262) | 2026.9 (1739.06,2344.54) | -0.53 (-0.63 to -0.43) |
| Indonesia | 3491473 (2991632,4049814) | 1714.11 (1468.85,1970.27) |  | 3910807 (3352053,4567803) | 1456.77 (1261.02,1689.92) | -0.55 (-0.66 to -0.44) |
| Iran (Islamic Republic of) | 1180951 (1017068,1368782) | 1709.14 (1477.27,1970.67) |  | 1249081 (1078595,1455290) | 1554.33 (1348.29,1790.9) | -0.44 (-0.54 to -0.33) |
| Iraq | 350131 (302953,405907) | 1615.48 (1376.95,1862.18) |  | 618594 (530991,723822) | 1427.6 (1228.76,1662.28) | -0.61 (-0.72 to -0.49) |
| Ireland | 40911 (34893,47347) | 1182.51 (1003.72,1369.26) |  | 41580 (34949,48474) | 953.94 (809.97,1104.11) | -0.7 (-0.74 to -0.65) |
| Israel | 61987 (53847,71304) | 1179.45 (1027.02,1364.58) |  | 100832 (85463,116802) | 1071.87 (903.97,1245.89) | -0.3 (-0.34 to -0.26) |
| Italy | 564248 (476627,664323) | 1137.54 (980.85,1316.7) |  | 520159 (438675,614349) | 1054.79 (914.72,1219.53) | -0.22 (-0.25 to -0.19) |
| Jamaica | 39724 (34222,46004) | 1570.18 (1353.77,1820.76) |  | 39770 (33890,47035) | 1521.32 (1311.56,1762.53) | -0.11 (-0.2 to -0.03) |
| Japan | 1238005 (1063775,1444744) | 1108.57 (963.96,1275.79) |  | 1042242 (891987,1200180) | 1035.9 (901.36,1197.6) | -0.21 (-0.24 to -0.17) |
| Jordan | 75910 (65609,87977) | 1671.37 (1443.21,1940.03) |  | 195110 (166531,229592) | 1549.73 (1333.73,1803.62) | -0.41 (-0.52 to -0.3) |
| Kazakhstan | 228390 (195512,265704) | 1370.4 (1176.01,1603.23) |  | 227053 (195742,267202) | 1204.07 (1035.27,1425.46) | -0.68 (-0.81 to -0.55) |
| Kenya | 515490 (443524,603217) | 1772.76 (1524.8,2052.16) |  | 954348 (815652,1120640) | 1734.22 (1492.62,2008.92) | -0.09 (-0.22 to 0.04) |
| Kiribati | 1485 (1271,1722) | 1788.08 (1516.22,2074.78) |  | 2246 (1919,2602) | 1769.24 (1510.29,2056.45) | 0 (-0.12 to 0.11) |
| Kuwait | 20933 (17915,24778) | 1204.69 (1048.27,1407.89) |  | 46561 (38758,55876) | 1162.55 (1006.99,1352.86) | -0.19 (-0.29 to -0.08) |
| Kyrgyzstan | 77500 (65842,90390) | 1584.72 (1347.03,1858.95) |  | 115078 (97541,134602) | 1620.3 (1368.67,1890.51) | -0.06 (-0.21 to 0.09) |
| Lao People's Democratic Republic | 91450 (79023,106010) | 1903.61 (1601.47,2225.25) |  | 110707 (94263,129917) | 1453.49 (1235.95,1695.4) | -1 (-1.12 to -0.88) |
| Latvia | 35358 (30255,41475) | 1446.69 (1244.53,1673.46) |  | 19745 (16908,23036) | 1273.79 (1102.53,1485.1) | -0.59 (-0.71 to -0.47) |
| Lebanon | 51257 (43952,59369) | 1562.95 (1336.4,1814.58) |  | 75720 (64344,88942) | 1458.59 (1259.16,1696.4) | -0.33 (-0.44 to -0.22) |
| Lesotho | 36278 (31758,41916) | 2093.96 (1784.29,2434.01) |  | 36849 (31567,42693) | 1853.01 (1591.2,2130.49) | -0.43 (-0.56 to -0.31) |
| Liberia | 49071 (42131,56751) | 2344.61 (1961.36,2766.53) |  | 136519 (115155,161324) | 2349.97 (1968.25,2776.01) | -0.23 (-0.37 to -0.1) |
| Libya | 64271 (55119,75070) | 1279.87 (1097.46,1483.65) |  | 96181 (80013,113858) | 1478.98 (1261.53,1724.55) | 0.42 (0.3 to 0.55) |
| Lithuania | 50329 (43467,58661) | 1450.03 (1251.31,1679.7) |  | 27832 (23668,32466) | 1245.89 (1082.15,1452.82) | -0.67 (-0.78 to -0.55) |
| Luxembourg | 3456 (2930,4048) | 1016.51 (864.48,1179.78) |  | 4840 (4068,5734) | 905.14 (766.07,1051.4) | -0.39 (-0.41 to -0.36) |
| Madagascar | 276104 (235167,323663) | 1927.39 (1629.5,2249.83) |  | 604477 (513848,718450) | 1905.12 (1612.94,2247.18) | -0.09 (-0.21 to 0.02) |
| Malawi | 243370 (207758,284352) | 2070.51 (1748.31,2399.68) |  | 433216 (370198,509885) | 2017.32 (1721.41,2352.09) | -0.14 (-0.27 to -0.01) |
| Malaysia | 278987 (238771,326372) | 1367.3 (1169.11,1592.37) |  | 346777 (294677,409902) | 1153.84 (983.17,1365.49) | -0.58 (-0.69 to -0.47) |
| Maldives | 4213 (3613,4936) | 1521.52 (1295.75,1761.64) |  | 5832 (4874,6979) | 1256.23 (1074.54,1480.02) | -0.7 (-0.82 to -0.59) |
| Mali | 228319 (194677,267159) | 2364.25 (1979.79,2802.68) |  | 555145 (477354,643507) | 2060.89 (1741,2415.91) | -0.51 (-0.63 to -0.38) |
| Malta | 4392 (3730,5152) | 1225.53 (1046.87,1428.62) |  | 3800 (3191,4450) | 1041.61 (889.77,1218.68) | -0.53 (-0.55 to -0.5) |
| Marshall Islands | 899 (772,1041) | 1675.48 (1413.52,1952.85) |  | 934 (789,1095) | 1609.38 (1370.51,1877.35) | -0.13 (-0.24 to -0.02) |
| Mauritania | 48009 (41286,56017) | 2009.72 (1701.54,2347.26) |  | 90574 (77186,105931) | 1865.05 (1572.14,2174.12) | -0.33 (-0.45 to -0.2) |
| Mauritius | 15870 (13538,18869) | 1404.97 (1206.17,1643.78) |  | 12995 (11036,15399) | 1194.45 (1014,1397.62) | -0.5 (-0.62 to -0.39) |
| Mexico | 1527552 (1332695,1752542) | 1540.87 (1343.89,1767.41) |  | 1825647 (1588983,2114796) | 1471.13 (1284.65,1689.46) | -0.19 (-0.27 to -0.11) |
| Micronesia (Federated States of) | 1985 (1703,2303) | 1689.46 (1453.52,1962.56) |  | 1714 (1443,2013) | 1629.96 (1381.81,1904.82) | -0.11 (-0.22 to 0) |
| Monaco | 186 (158,216) | 818.69 (697.05,952.31) |  | 211 (176,245) | 757.7 (637.13,889.7) | -0.28 (-0.29 to -0.27) |
| Mongolia | 40174 (34322,46418) | 1657.35 (1399.45,1925.49) |  | 46747 (40090,54580) | 1349.05 (1159.82,1576.76) | -0.87 (-0.99 to -0.75) |
| Montenegro | 8411 (7129,9944) | 1365.58 (1168.9,1598) |  | 7016 (5944,8279) | 1260.76 (1085.8,1476.36) | -0.46 (-0.58 to -0.33) |
| Morocco | 495479 (427223,576706) | 1757.24 (1510.4,2049.94) |  | 581246 (497841,679606) | 1586.08 (1369.85,1847.78) | -0.37 (-0.48 to -0.25) |
| Mozambique | 341027 (291432,399348) | 2327.71 (1944.16,2743.32) |  | 678534 (576436,797334) | 1934.13 (1630.1,2260.86) | -0.75 (-0.87 to -0.63) |
| Myanmar | 914041 (776148,1072746) | 2102.48 (1789.17,2456.25) |  | 838092 (709557,983347) | 1486.4 (1258.92,1740.54) | -1.38 (-1.51 to -1.26) |
| Namibia | 28931 (25074,33500) | 1680.39 (1446.24,1947.5) |  | 39804 (34256,46436) | 1527.42 (1327.19,1778.38) | -0.4 (-0.53 to -0.28) |
| Nauru | 149 (128,174) | 1258.27 (1079.77,1473.13) |  | 157 (134,182) | 1310.32 (1124.85,1527.21) | 0.1 (-0.08 to 0.27) |
| Nepal | 586198 (501319,683622) | 2650.23 (2234.47,3118.73) |  | 737392 (624814,868271) | 2291.21 (1943.2,2668.48) | -0.48 (-0.58 to -0.38) |
| Netherlands | 148787 (125729,173804) | 1099.37 (937.07,1272.13) |  | 142301 (119182,167740) | 990.76 (836.66,1153.53) | -0.37 (-0.4 to -0.34) |
| New Zealand | 31293 (26880,35978) | 973.34 (839.9,1118.97) |  | 38633 (33144,44553) | 898.34 (764.94,1039.42) | -0.3 (-0.34 to -0.27) |
| Nicaragua | 83792 (73030,96474) | 1807.04 (1548.59,2071.97) |  | 113882 (97961,133296) | 1680.03 (1456.7,1949.1) | -0.33 (-0.42 to -0.24) |
| Niger | 220951 (189227,258976) | 2403.7 (2018.62,2846.66) |  | 648663 (553757,753296) | 2381.99 (1981.32,2822.83) | -0.07 (-0.2 to 0.05) |
| Nigeria | 2224637 (1918857,2596207) | 2084.93 (1766.22,2438.88) |  | 4739987 (4062758,5543116) | 1800.74 (1538.35,2085.54) | -0.82 (-0.94 to -0.69) |
| Niue | 33 (29,39) | 1428.39 (1221.14,1657.15) |  | 20 (17,24) | 1296.89 (1106.68,1515.33) | -0.41 (-0.52 to -0.31) |
| North Macedonia | 27976 (23613,33054) | 1425.75 (1208.15,1667.84) |  | 25201 (21270,29923) | 1309.56 (1109.39,1515.87) | -0.4 (-0.52 to -0.28) |
| Northern Mariana Islands | 508 (433,597) | 1066.18 (907.8,1249.9) |  | 502 (431,589) | 1109.7 (950.2,1298.99) | 0.3 (0.19 to 0.4) |
| Norway | 44529 (38309,51714) | 1182.06 (1018.01,1371.16) |  | 48072 (41368,56165) | 1070.05 (920.01,1241.67) | -0.37 (-0.39 to -0.35) |
| Oman | 30339 (25825,35248) | 1277.34 (1099.02,1484.09) |  | 54612 (45883,64460) | 1193.48 (1022.43,1388.53) | -0.33 (-0.43 to -0.23) |
| Pakistan | 3017495 (2593369,3507598) | 2282.98 (1944.66,2638.61) |  | 5389921 (4630577,6263912) | 2087.68 (1792.7,2406.6) | -0.32 (-0.39 to -0.25) |
| Palau | 215 (182,253) | 1334.43 (1144.12,1557.5) |  | 201 (171,239) | 1253.01 (1080.17,1466.61) | -0.22 (-0.32 to -0.11) |
| Palestine | 47196 (40854,54560) | 1907.67 (1610.52,2223.31) |  | 96606 (82386,112698) | 1759.28 (1505.65,2060.91) | -0.24 (-0.36 to -0.13) |
| Panama | 41273 (35931,47466) | 1565.92 (1357.66,1816.55) |  | 56001 (48214,65538) | 1331.43 (1149.59,1546.85) | -0.62 (-0.7 to -0.53) |
| Papua New Guinea | 80648 (68985,93356) | 1696.13 (1449.47,1968.81) |  | 178717 (153863,208852) | 1586.14 (1346.8,1843.32) | -0.18 (-0.29 to -0.07) |
| Paraguay | 71789 (61908,83378) | 1528.07 (1308.02,1768.45) |  | 99032 (85068,115208) | 1390.19 (1203.85,1612.65) | -0.34 (-0.43 to -0.25) |
| Peru | 381806 (330920,442450) | 1588.48 (1367.44,1841.03) |  | 493614 (424281,575563) | 1385.77 (1194.64,1605.19) | -0.59 (-0.67 to -0.5) |
| Philippines | 1258000 (1085194,1459346) | 1720.13 (1486.62,1980.55) |  | 1763855 (1520832,2046264) | 1522.47 (1318.53,1763.81) | -0.41 (-0.52 to -0.3) |
| Poland | 533767 (455625,621570) | 1449.97 (1248.17,1675.44) |  | 394778 (338287,461599) | 1219.33 (1053.4,1415.4) | -0.63 (-0.75 to -0.52) |
| Portugal | 108072 (90964,127461) | 1172.55 (994.82,1375.47) |  | 92865 (76802,110066) | 1053.04 (893.26,1234.96) | -0.35 (-0.39 to -0.32) |
| Puerto Rico | 46309 (40096,53627) | 1290.95 (1115.9,1489.15) |  | 31485 (26937,36870) | 1212.39 (1043.29,1401.78) | -0.22 (-0.3 to -0.13) |
| Qatar | 5686 (4807,6647) | 1213.92 (1051.27,1415.76) |  | 26584 (21702,31948) | 1101.36 (942.32,1280.98) | -0.47 (-0.57 to -0.36) |
| Republic of Korea | 527313 (436104,635079) | 1195.12 (1005.81,1438.35) |  | 399539 (329632,477036) | 958.09 (819.44,1109.86) | -0.75 (-0.84 to -0.66) |
| Republic of Moldova | 73501 (62459,86049) | 1671.6 (1421.07,1946.26) |  | 49875 (41422,58926) | 1599.92 (1364.52,1865.61) | -0.31 (-0.46 to -0.17) |
| Romania | 312406 (262782,366935) | 1396.81 (1182.94,1631.42) |  | 195720 (164506,231567) | 1194.44 (1023.92,1391.77) | -0.68 (-0.8 to -0.56) |
| Russian Federation | 2046265 (1786447,2370967) | 1447.32 (1267.13,1666.16) |  | 1708388 (1480441,1976476) | 1373.88 (1203.74,1581.06) | -0.37 (-0.49 to -0.25) |
| Rwanda | 170383 (145913,199364) | 2007.76 (1682.9,2337.38) |  | 251705 (213114,295989) | 1753 (1484.61,2040.58) | -0.62 (-0.75 to -0.48) |
| Saint Kitts and Nevis | 608 (525,702) | 1406.48 (1211.93,1630.17) |  | 643 (548,763) | 1262.4 (1092.11,1473.4) | -0.37 (-0.45 to -0.28) |
| Saint Lucia | 2284 (1971,2664) | 1517.18 (1306.3,1765.38) |  | 2238 (1892,2633) | 1424.43 (1228.46,1645.1) | -0.22 (-0.31 to -0.14) |
| Saint Vincent and the Grenadines | 1864 (1611,2160) | 1598.88 (1371.52,1850.96) |  | 1551 (1323,1821) | 1455.45 (1249.02,1691) | -0.34 (-0.43 to -0.26) |
| Samoa | 3040 (2601,3545) | 1608.69 (1369.05,1872.26) |  | 3400 (2917,3943) | 1470.84 (1259.06,1720.26) | -0.35 (-0.46 to -0.24) |
| San Marino | 210 (177,247) | 1013.55 (865.26,1177.13) |  | 258 (216,309) | 978.36 (831.4,1141.4) | -0.13 (-0.18 to -0.07) |
| Sao Tome and Principe | 2909 (2499,3372) | 2097.78 (1763.23,2440.05) |  | 4385 (3757,5155) | 1899.32 (1623.27,2207.83) | -0.44 (-0.57 to -0.31) |
| Saudi Arabia | 249229 (215089,290212) | 1271.02 (1097.41,1485.06) |  | 410289 (345666,489376) | 1209.73 (1042.86,1417.77) | -0.26 (-0.36 to -0.16) |
| Senegal | 182145 (156624,212426) | 2025.85 (1713.39,2374.86) |  | 331634 (284538,387365) | 1917.03 (1619.84,2244.9) | -0.22 (-0.34 to -0.09) |
| Serbia | 127253 (107499,149199) | 1382.63 (1184.56,1597.67) |  | 99229 (83024,116463) | 1284.78 (1101.51,1494.94) | -0.46 (-0.58 to -0.33) |
| Seychelles | 999 (857,1168) | 1296.11 (1110.99,1520.63) |  | 1121 (948,1311) | 1152.54 (980.29,1356.56) | -0.37 (-0.48 to -0.26) |
| Sierra Leone | 98407 (84029,115733) | 2187.88 (1833.93,2577.01) |  | 211450 (177818,248466) | 2217.02 (1855.29,2602.84) | -0.04 (-0.18 to 0.11) |
| Singapore | 31174 (26215,37130) | 1039.48 (897.33,1213.05) |  | 39763 (33417,46911) | 851.7 (733.24,997.27) | -0.72 (-0.75 to -0.68) |
| Slovakia | 66198 (56219,77627) | 1299.42 (1109.35,1507.78) |  | 53355 (45245,62896) | 1146.47 (981.77,1344.39) | -0.55 (-0.66 to -0.43) |
| Slovenia | 22472 (19014,26605) | 1234.49 (1055.48,1448.36) |  | 19767 (16649,23476) | 1130.52 (969.33,1320.85) | -0.37 (-0.48 to -0.25) |
| Solomon Islands | 7220 (6239,8372) | 1806.12 (1527.25,2094.96) |  | 12805 (11021,14810) | 1748.8 (1486.13,2028.15) | -0.05 (-0.17 to 0.06) |
| Somalia | 219880 (188644,257204) | 2670.58 (2251.04,3162.54) |  | 665267 (563809,784523) | 2925.5 (2451.33,3494.39) | 0.25 (0.13 to 0.37) |
| South Africa | 719666 (623586,831892) | 1687.95 (1468.63,1936.95) |  | 899353 (781174,1043714) | 1613.32 (1410.54,1865.96) | -0.23 (-0.34 to -0.11) |
| South Sudan | 109400 (93958,128184) | 1529.05 (1316.78,1789.32) |  | 193041 (164146,229011) | 1763.3 (1490.32,2091.95) | 0.37 (0.24 to 0.51) |
| Spain | 428028 (360356,501399) | 1255.01 (1072.19,1445.1) |  | 418487 (350888,495551) | 1132.6 (965.48,1306.83) | -0.35 (-0.38 to -0.32) |
| Sri Lanka | 299078 (252057,351479) | 1661.74 (1408.26,1934.74) |  | 274782 (233342,322683) | 1315.56 (1121.95,1547.14) | -0.85 (-0.96 to -0.73) |
| Sudan | 474584 (407561,551837) | 2024.99 (1713.14,2365.21) |  | 837411 (717570,974017) | 1788.52 (1513.95,2081.7) | -0.55 (-0.66 to -0.43) |
| Suriname | 6078 (5250,7041) | 1489 (1290.96,1727.1) |  | 7761 (6666,9014) | 1393.46 (1204.83,1603.12) | -0.35 (-0.44 to -0.27) |
| Sweden | 91040 (77689,106191) | 1176.26 (1007.41,1366.8) |  | 95115 (81546,110966) | 1077.93 (920.34,1244.61) | -0.36 (-0.39 to -0.34) |
| Switzerland | 62950 (53031,74242) | 1035.98 (879.38,1201.86) |  | 70607 (59688,83297) | 956.8 (821.37,1108.07) | -0.3 (-0.31 to -0.29) |
| Syrian Arab Republic | 274404 (237085,319603) | 1807.49 (1529.6,2106.25) |  | 245984 (208186,290604) | 1756.12 (1508.65,2045.56) | -0.17 (-0.28 to -0.05) |
| Taiwan (Province of China) | 232003 (197119,275724) | 1144.5 (989.03,1342.12) |  | 185414 (156566,218634) | 969.33 (835.97,1131.44) | -0.55 (-0.66 to -0.44) |
| Tajikistan | 99693 (85677,115280) | 1625.36 (1366.29,1901.98) |  | 180739 (153727,210946) | 1700.45 (1437.36,2004.62) | -0.1 (-0.27 to 0.08) |
| Thailand | 891680 (758466,1041503) | 1511.54 (1298.3,1727.96) |  | 729388 (613909,857061) | 1290.72 (1105.8,1501.18) | -0.54 (-0.66 to -0.41) |
| Timor-Leste | 15700 (13374,18330) | 1737.87 (1462.95,2022.52) |  | 22463 (19097,26205) | 1481.65 (1255.97,1725.32) | -0.77 (-0.91 to -0.64) |
| Togo | 94677 (80729,110995) | 2221.6 (1869.65,2621.31) |  | 194211 (165236,228210) | 2166.25 (1822.75,2537.17) | -0.07 (-0.2 to 0.06) |
| Tokelau | 26 (23,31) | 1599.62 (1369.47,1864.91) |  | 19 (16,23) | 1421.8 (1220.03,1656.2) | -0.47 (-0.58 to -0.37) |
| Tonga | 1752 (1500,2023) | 1590.6 (1355.34,1855.15) |  | 1683 (1452,1944) | 1470.99 (1258.3,1702.99) | -0.24 (-0.35 to -0.13) |
| Trinidad and Tobago | 18176 (15680,21000) | 1430.24 (1237.23,1649.27) |  | 14981 (12827,17461) | 1224.6 (1052.16,1424.09) | -0.72 (-0.81 to -0.63) |
| Tunisia | 156108 (134141,181764) | 1680.03 (1433.31,1959.39) |  | 168267 (143401,196700) | 1500.33 (1283.78,1744.28) | -0.39 (-0.5 to -0.28) |
| Turkey | 965613 (824149,1125957) | 1530.55 (1317.81,1771.27) |  | 1030574 (870903,1207249) | 1322.89 (1127.74,1531.16) | -0.56 (-0.67 to -0.45) |
| Turkmenistan | 61969 (53193,71919) | 1474.91 (1262.23,1718.72) |  | 65146 (55915,76416) | 1242.97 (1066.65,1457.49) | -0.84 (-0.99 to -0.7) |
| Tuvalu | 176 (150,203) | 1756.23 (1491.76,2046) |  | 201 (171,234) | 1596.25 (1353.8,1846.29) | -0.25 (-0.37 to -0.14) |
| Uganda | 418167 (358440,488502) | 2013.7 (1687.89,2351.79) |  | 870090 (746475,1024976) | 1757.56 (1489.54,2046.73) | -0.54 (-0.66 to -0.42) |
| Ukraine | 750015 (652040,867613) | 1548.45 (1346.66,1780.03) |  | 571702 (488282,667669) | 1573.16 (1358.86,1808.26) | -0.13 (-0.25 to -0.01) |
| United Arab Emirates | 22970 (19488,26799) | 1072.81 (923.08,1247.56) |  | 90425 (72290,112459) | 1155.9 (991.7,1341.94) | 0.29 (0.2 to 0.39) |
| United Kingdom | 616131 (526286,721747) | 1186.59 (1016.66,1376.7) |  | 636922 (543299,740404) | 1089.9 (937.28,1261.26) | -0.29 (-0.31 to -0.27) |
| United Republic of Tanzania | 569239 (492908,659020) | 1850.16 (1580.57,2134.39) |  | 1097015 (935056,1280635) | 1671.96 (1424.55,1950.93) | -0.45 (-0.58 to -0.33) |
| United States of America | 2332106 (2044089,2660363) | 986.02 (870.84,1109.77) |  | 2599422 (2263298,2948567) | 934.8 (821.5,1051.57) | -0.23 (-0.31 to -0.15) |
| United States Virgin Islands | 1356 (1168,1568) | 1250.11 (1080.23,1434.58) |  | 804 (686,953) | 1161.14 (1002.96,1353.01) | -0.28 (-0.38 to -0.19) |
| Uruguay | 36921 (31770,42736) | 1205.89 (1043.07,1395.7) |  | 31919 (27312,37279) | 1067.27 (918.52,1242.85) | -0.44 (-0.48 to -0.41) |
| Uzbekistan | 386405 (328977,447853) | 1634.31 (1384.91,1908.85) |  | 497772 (425058,578367) | 1437.64 (1230.25,1670.24) | -0.56 (-0.69 to -0.44) |
| Vanuatu | 3059 (2642,3542) | 1726.52 (1472.76,2001.61) |  | 5608 (4820,6562) | 1672.21 (1426.2,1954.48) | -0.1 (-0.21 to 0.01) |
| Venezuela (Bolivarian Republic of) | 313792 (271360,360654) | 1454.29 (1261.52,1691.72) |  | 376733 (324185,440521) | 1471.93 (1270.04,1709.25) | -0.13 (-0.22 to -0.03) |
| Viet Nam | 1385809 (1168111,1623946) | 1837.23 (1550.07,2127.68) |  | 1358231 (1142941,1584348) | 1406.07 (1187.37,1634.61) | -0.94 (-1.05 to -0.83) |
| Yemen | 317390 (275227,367277) | 1925.83 (1633.07,2249.08) |  | 715393 (603132,838503) | 1977.95 (1650.09,2318.39) | -0.07 (-0.2 to 0.06) |
| Zambia | 171637 (147037,202300) | 1784.51 (1511.17,2083.42) |  | 357668 (301610,422628) | 1605.23 (1355.99,1889.81) | -0.55 (-0.68 to -0.42) |
| Zimbabwe | 226683 (195824,263090) | 1811.76 (1552.18,2105.88) |  | 333979 (287960,387932) | 1945.91 (1654.28,2266.36) | 0.41 (0.28 to 0.54) |
| ASPR, age-standardized prevalence rate. EAPC, estimated annual percentage changes. | | | | | | |

| **Table S3** The cases of DALYs and ASDR of otitis media in 1992 and 2021, and its temporal trends from 1992-2021 among 204 countries and territories. | | | | | | |
| --- | --- | --- | --- | --- | --- | --- |
| **Characteristics** | **1992** | |  | **2021** | | **1992-2021** |
|  | **Cases of DALYs (95%UI)** | **ASDR per 100,000 (95%UI)** |  | **Cases of DALYs (95%UI)** | **ASDR per 100,000 (95%UI)** | **EAPC (95%CI)** |
| Afghanistan | 4726 (2785,7386) | 42.47 (24.93,66.55) |  | 14482 (8382,23390) | 42.01 (24.81,67.05) | -0.29 (-0.45 to -0.13) |
| Albania | 1286 (776,1991) | 36.93 (22.32,57.23) |  | 656 (384,1027) | 27.16 (15.79,43.9) | -1.12 (-1.25 to -0.98) |
| Algeria | 9223 (5396,14724) | 30.97 (18.07,49.57) |  | 13197 (7685,21058) | 29.46 (17.17,47.12) | -0.29 (-0.43 to -0.16) |
| American Samoa | 14 (8,22) | 23.82 (13.67,38.39) |  | 12 (7,20) | 24.84 (14.26,40.06) | 0.12 (0 to 0.24) |
| Andorra | 10 (6,16) | 18.26 (10.29,29.37) |  | 12 (7,20) | 17.25 (9.84,27.38) | -0.21 (-0.26 to -0.17) |
| Angola | 4560 (2703,7275) | 36.47 (21.66,58.4) |  | 11849 (6916,19172) | 31.16 (18.29,50.26) | -0.77 (-0.91 to -0.63) |
| Antigua and Barbuda | 17 (10,27) | 26.94 (15.71,42.69) |  | 20 (12,33) | 25.21 (14.51,40.15) | -0.22 (-0.32 to -0.11) |
| Argentina | 7868 (4556,12618) | 22.97 (13.3,36.88) |  | 8841 (5259,14166) | 20.84 (12.26,33.48) | -0.37 (-0.42 to -0.32) |
| Armenia | 1152 (666,1861) | 33.09 (19.18,53.32) |  | 779 (457,1282) | 28.35 (16.51,45.86) | -0.85 (-1 to -0.7) |
| Australia | 2545 (1479,4058) | 16.16 (9.33,25.59) |  | 3158 (1850,4989) | 14.67 (8.36,23.48) | -0.38 (-0.5 to -0.26) |
| Austria | 1578 (989,2428) | 22.55 (14.11,34.65) |  | 1402 (825,2237) | 17.71 (10.21,27.83) | -0.69 (-0.81 to -0.58) |
| Azerbaijan | 2300 (1334,3744) | 29.15 (17.25,46.96) |  | 2575 (1491,4267) | 25.77 (14.91,42.23) | -0.89 (-1.08 to -0.69) |
| Bahamas | 67 (38,107) | 24.37 (14.04,38.49) |  | 87 (50,137) | 24.17 (13.88,38.21) | -0.1 (-0.22 to 0.02) |
| Bahrain | 144 (82,235) | 25.9 (14.77,41.94) |  | 339 (197,542) | 24.34 (14.24,39.18) | -0.26 (-0.39 to -0.13) |
| Bangladesh | 68342 (39166,110355) | 54.96 (32.13,88.82) |  | 73176 (42249,117563) | 43.96 (25.46,70.29) | -0.83 (-0.91 to -0.74) |
| Barbados | 65 (37,104) | 26.93 (15.66,43.18) |  | 70 (41,113) | 27.08 (15.68,42.76) | 0.06 (-0.05 to 0.17) |
| Belarus | 2948 (1767,4703) | 29.82 (17.82,47.75) |  | 2164 (1277,3538) | 27.03 (15.85,43.8) | -0.55 (-0.68 to -0.41) |
| Belgium | 1985 (1179,3087) | 22.06 (13.02,34.05) |  | 1915 (1104,3105) | 19.17 (11.05,30.45) | -0.45 (-0.51 to -0.39) |
| Belize | 70 (41,111) | 32.26 (18.82,51.93) |  | 131 (76,210) | 29.97 (17.45,48.15) | -0.23 (-0.34 to -0.12) |
| Benin | 2585 (1489,4099) | 45.23 (26.53,72.98) |  | 6252 (3618,10028) | 41.76 (24.56,67.82) | -0.33 (-0.47 to -0.19) |
| Bermuda | 13 (7,21) | 23.46 (13.51,38.3) |  | 11 (6,17) | 21.6 (12.31,34.31) | -0.34 (-0.45 to -0.24) |
| Bhutan | 341 (198,555) | 47.47 (28.12,76.1) |  | 276 (162,449) | 37.3 (21.99,60.17) | -0.86 (-0.95 to -0.78) |
| Bolivia (Plurinational State of) | 2435 (1414,3940) | 34.17 (20.11,55.16) |  | 3586 (2131,5766) | 30 (17.92,48) | -0.5 (-0.6 to -0.4) |
| Bosnia and Herzegovina | 1836 (1119,2912) | 41.42 (25.28,64.66) |  | 790 (463,1261) | 27.21 (15.9,43.44) | -1.56 (-1.72 to -1.39) |
| Botswana | 530 (309,855) | 32.72 (19.14,52.07) |  | 683 (397,1098) | 28.03 (16.4,45.7) | -0.53 (-0.66 to -0.4) |
| Brazil | 56739 (36553,87387) | 35.43 (23,53.65) |  | 60112 (36125,96175) | 29.19 (17.32,46.4) | -0.4 (-0.53 to -0.26) |
| Brunei Darussalam | 48 (28,76) | 16.48 (9.48,25.83) |  | 69 (39,111) | 16.54 (9.34,26.46) | -0.06 (-0.1 to -0.02) |
| Bulgaria | 2286 (1386,3683) | 29.82 (18.24,47.02) |  | 1430 (838,2304) | 24.8 (14.27,39.89) | -0.68 (-0.81 to -0.55) |
| Burkina Faso | 5276 (3063,8417) | 49.01 (28.27,78.27) |  | 11090 (6353,17851) | 44.55 (26.25,71.13) | -0.36 (-0.5 to -0.22) |
| Burundi | 3668 (2016,6207) | 52.33 (29.53,83.24) |  | 7137 (4052,11240) | 49 (28.16,77.31) | -0.24 (-0.4 to -0.08) |
| Cabo Verde | 175 (103,281) | 43.03 (25.37,68.79) |  | 192 (112,323) | 34.66 (20.2,57.04) | -0.84 (-0.98 to -0.7) |
| Cambodia | 4878 (2845,7760) | 41.33 (24.2,65.4) |  | 5723 (3344,9256) | 32.94 (19.23,53.89) | -0.89 (-1.03 to -0.75) |
| Cameroon | 5197 (3022,8240) | 41.33 (24.14,66.93) |  | 14272 (8204,23139) | 40.32 (23.72,64.78) | -0.23 (-0.38 to -0.09) |
| Canada | 4925 (2899,7793) | 19.3 (11.25,30.65) |  | 5552 (3251,8664) | 17.61 (10.19,28.08) | -0.31 (-0.37 to -0.25) |
| Central African Republic | 1410 (831,2225) | 44.72 (26.45,70.62) |  | 2810 (1634,4445) | 47.92 (28.32,74.88) | 0.22 (0.08 to 0.35) |
| Chad | 3347 (1933,5303) | 48.45 (28.36,77.55) |  | 8640 (5056,13966) | 43.45 (25.76,70.08) | -0.53 (-0.68 to -0.39) |
| Chile | 3500 (2096,5510) | 25.13 (15.12,39.39) |  | 3379 (1982,5398) | 20.08 (11.65,32.17) | -0.79 (-0.85 to -0.74) |
| China | 459897 (270545,748033) | 37.39 (22.16,59.51) |  | 336499 (196870,547209) | 26.53 (15.31,42.96) | -1.26 (-1.39 to -1.12) |
| Colombia | 12828 (8254,19880) | 35.35 (22.49,54.4) |  | 13184 (7862,21167) | 28.6 (17.08,45.8) | -0.64 (-0.76 to -0.51) |
| Comoros | 243 (140,388) | 41.79 (25.14,65.29) |  | 285 (166,454) | 37.01 (21.53,58.86) | -0.42 (-0.53 to -0.3) |
| Congo | 982 (561,1564) | 34.4 (19.92,55.05) |  | 1870 (1092,2987) | 32.39 (19.02,51.5) | -0.36 (-0.49 to -0.22) |
| Cook Islands | 5 (3,8) | 24.7 (14.3,39.75) |  | 3 (2,6) | 21.47 (12.29,34.2) | -0.43 (-0.55 to -0.31) |
| Costa Rica | 1062 (618,1721) | 30.82 (18.18,49.02) |  | 1220 (718,1972) | 27.61 (16.03,44.65) | -0.4 (-0.51 to -0.3) |
| Croatia | 1241 (732,1989) | 28.41 (16.99,44.61) |  | 856 (514,1375) | 24.02 (14.01,38.68) | -0.6 (-0.74 to -0.47) |
| Cuba | 3365 (1973,5548) | 31.5 (18.43,51.4) |  | 3037 (1789,4883) | 30.16 (17.48,48.22) | -0.36 (-0.48 to -0.23) |
| Cyprus | 171 (99,277) | 21.87 (12.6,35.48) |  | 238 (138,386) | 19.68 (11.26,31.08) | -0.35 (-0.41 to -0.3) |
| Czechia | 2581 (1606,3986) | 28.14 (17.99,43.41) |  | 2050 (1182,3311) | 22.57 (13.06,36.53) | -0.64 (-0.79 to -0.48) |
| C么te d'Ivoire | 5981 (3424,9637) | 40.3 (23.46,65.15) |  | 11854 (6898,18846) | 38.55 (22.8,61.77) | -0.12 (-0.26 to 0.03) |
| Democratic People's Republic of Korea | 7197 (4197,11752) | 33.42 (19.54,54.03) |  | 9110 (5404,14483) | 35.41 (20.74,56.43) | 0.14 (0 to 0.28) |
| Democratic Republic of the Congo | 18666 (10947,29623) | 41.67 (24.61,66.98) |  | 44698 (25734,71900) | 46.27 (26.8,74.57) | 0.27 (0.1 to 0.44) |
| Denmark | 971 (562,1534) | 20.78 (11.95,32.97) |  | 934 (529,1484) | 18.24 (10.31,28.76) | -0.43 (-0.48 to -0.38) |
| Djibouti | 212 (124,345) | 37.48 (22,58.93) |  | 443 (260,700) | 33.74 (19.89,53.01) | -0.42 (-0.49 to -0.34) |
| Dominica | 23 (13,37) | 30.69 (17.8,49.14) |  | 18 (11,29) | 28.92 (16.62,45.81) | -0.22 (-0.32 to -0.11) |
| Dominican Republic | 2534 (1476,4138) | 31.87 (18.7,51.33) |  | 2929 (1688,4740) | 26.92 (15.53,43.51) | -0.61 (-0.72 to -0.51) |
| Ecuador | 3420 (1978,5443) | 30.39 (17.72,48.24) |  | 5113 (2967,8131) | 28.34 (16.41,45.14) | -0.35 (-0.46 to -0.24) |
| Egypt | 22674 (13093,36331) | 36.09 (20.72,57.7) |  | 34894 (20275,54820) | 30.9 (18.06,48.04) | -0.58 (-0.71 to -0.45) |
| El Salvador | 2039 (1194,3272) | 34.76 (20.33,56.23) |  | 1999 (1154,3185) | 30.92 (17.82,49.27) | -0.42 (-0.53 to -0.32) |
| Equatorial Guinea | 207 (123,332) | 40.33 (23.76,64.45) |  | 424 (244,689) | 24.86 (14.49,40.16) | -2.08 (-2.34 to -1.82) |
| Eritrea | 1857 (1039,3125) | 49.54 (28.58,77.42) |  | 2954 (1719,4621) | 41.21 (24.1,64.86) | -0.4 (-0.52 to -0.29) |
| Estonia | 483 (305,750) | 33.24 (20.9,51.2) |  | 274 (163,436) | 25.04 (14.66,39.79) | -0.97 (-1.1 to -0.84) |
| Eswatini | 341 (197,552) | 33.92 (19.77,54.44) |  | 389 (226,616) | 30.92 (17.91,49.61) | -0.29 (-0.42 to -0.16) |
| Ethiopia | 42774 (23505,72302) | 63.27 (34.67,99.69) |  | 51476 (29954,81612) | 42.23 (24.67,66.93) | -1.55 (-1.68 to -1.42) |
| Fiji | 244 (144,391) | 29.46 (17.49,47.06) |  | 250 (143,410) | 26.63 (15.28,43.68) | -0.31 (-0.43 to -0.18) |
| Finland | 1025 (600,1687) | 21.75 (12.7,35.58) |  | 872 (496,1387) | 18.54 (10.52,29.72) | -0.48 (-0.52 to -0.43) |
| France | 12299 (7434,19038) | 23.41 (14.18,36.53) |  | 11586 (6726,18716) | 20.1 (11.55,32.33) | -0.48 (-0.56 to -0.4) |
| Gabon | 317 (182,507) | 27.01 (15.49,43.18) |  | 521 (305,839) | 26.6 (15.52,41.92) | -0.08 (-0.21 to 0.05) |
| Gambia | 511 (293,815) | 43.36 (25.46,68.89) |  | 1144 (649,1840) | 43.74 (25.32,70.66) | 0.02 (-0.12 to 0.16) |
| Georgia | 1574 (931,2541) | 29.81 (17.81,47.93) |  | 916 (532,1493) | 27.71 (16.18,44.98) | -0.63 (-0.81 to -0.46) |
| Germany | 16351 (10006,25154) | 23.14 (14.12,35.1) |  | 13570 (8076,21647) | 18.65 (10.87,29.37) | -0.64 (-0.73 to -0.55) |
| Ghana | 7375 (4269,11736) | 42.62 (24.83,68.45) |  | 13613 (7798,21780) | 36.56 (21.05,58.7) | -0.56 (-0.7 to -0.42) |
| Greece | 2205 (1308,3448) | 22.76 (13.19,36.07) |  | 1836 (1080,2976) | 21.02 (12.04,33.92) | -0.3 (-0.36 to -0.23) |
| Greenland | 9 (6,15) | 17.42 (10.09,27.91) |  | 8 (5,13) | 15.88 (8.98,25.48) | -0.4 (-0.46 to -0.33) |
| Grenada | 29 (17,47) | 31.43 (18.41,50.11) |  | 27 (16,43) | 27.47 (16.02,44.01) | -0.48 (-0.59 to -0.37) |
| Guam | 31 (18,50) | 20.82 (12.09,33.47) |  | 30 (17,48) | 20.06 (11.63,32.65) | -0.16 (-0.28 to -0.04) |
| Guatemala | 3467 (2066,5526) | 34.3 (20.17,55.17) |  | 5090 (2995,8098) | 31.07 (18.41,49.05) | -0.34 (-0.45 to -0.24) |
| Guinea | 3108 (1785,4950) | 44.25 (26.26,71.35) |  | 6322 (3715,10177) | 42.76 (25.61,68.96) | -0.07 (-0.21 to 0.08) |
| Guinea-Bissau | 530 (306,856) | 45.93 (26.99,73.39) |  | 1015 (584,1629) | 44.95 (26.31,71.79) | -0.02 (-0.16 to 0.12) |
| Guyana | 289 (170,460) | 34.91 (20.65,55.94) |  | 227 (134,362) | 29.56 (17.52,46.9) | -0.56 (-0.69 to -0.44) |
| Haiti | 2755 (1595,4407) | 38.68 (22.38,61.95) |  | 5283 (3041,8547) | 39.7 (23.19,63.35) | 0.06 (-0.05 to 0.18) |
| Honduras | 2009 (1188,3248) | 36.31 (21.76,58.36) |  | 3552 (2122,5742) | 33.71 (20.14,53.93) | -0.27 (-0.37 to -0.18) |
| Hungary | 3472 (2368,5040) | 40.5 (28.54,57.31) |  | 1962 (1149,3141) | 24.09 (14.11,38.69) | -1.28 (-1.52 to -1.04) |
| Iceland | 53 (31,84) | 21.21 (12.31,33.64) |  | 59 (35,94) | 18.66 (10.72,29.71) | -0.44 (-0.51 to -0.37) |
| India | 492456 (291595,784318) | 51.04 (30.42,81.21) |  | 584652 (338758,945963) | 41.56 (24.23,66.89) | -0.6 (-0.72 to -0.49) |
| Indonesia | 71232 (41712,115023) | 34.93 (20.62,56.35) |  | 79327 (46355,128722) | 29.39 (17.08,47.63) | -0.58 (-0.71 to -0.44) |
| Iran (Islamic Republic of) | 23868 (13998,38168) | 34.56 (20.15,55.11) |  | 25152 (14701,40343) | 31.13 (18.09,49.93) | -0.48 (-0.61 to -0.35) |
| Iraq | 7058 (4189,11245) | 32.72 (19.3,52.22) |  | 12416 (7167,19609) | 28.55 (16.67,44.95) | -0.66 (-0.79 to -0.52) |
| Ireland | 804 (476,1273) | 23.16 (13.74,36.66) |  | 786 (457,1261) | 17.61 (10.11,27.97) | -0.88 (-0.95 to -0.8) |
| Israel | 1223 (735,1913) | 23.34 (14,36.75) |  | 1894 (1095,3014) | 20.1 (11.59,32.12) | -0.44 (-0.5 to -0.38) |
| Italy | 11254 (6634,17682) | 22.17 (13.04,34.82) |  | 10359 (6135,16459) | 20.36 (11.82,32.13) | -0.28 (-0.35 to -0.22) |
| Jamaica | 786 (457,1273) | 31.21 (18.19,50.04) |  | 797 (457,1290) | 30.09 (17.38,48.36) | -0.13 (-0.24 to -0.02) |
| Japan | 24452 (14350,38950) | 21.39 (12.29,34.19) |  | 20448 (12134,33117) | 19.78 (11.31,31.77) | -0.23 (-0.27 to -0.19) |
| Jordan | 1541 (901,2478) | 34.04 (19.76,55.07) |  | 3968 (2300,6390) | 31.32 (18.22,50.38) | -0.44 (-0.58 to -0.3) |
| Kazakhstan | 4575 (2636,7360) | 27.47 (15.93,44.36) |  | 4517 (2596,7267) | 23.96 (13.75,38.53) | -0.72 (-0.86 to -0.57) |
| Kenya | 11531 (6784,18186) | 39.28 (23.59,61.32) |  | 20048 (11840,31985) | 36.57 (21.48,58.86) | -0.21 (-0.35 to -0.06) |
| Kiribati | 30 (17,48) | 36.32 (21.27,57.93) |  | 45 (26,74) | 35.59 (20.84,57.83) | -0.03 (-0.16 to 0.1) |
| Kuwait | 415 (239,672) | 23.81 (13.59,38.42) |  | 926 (542,1485) | 22.84 (13.21,36.55) | -0.21 (-0.34 to -0.08) |
| Kyrgyzstan | 1571 (919,2592) | 32.23 (18.88,52.46) |  | 2339 (1378,3840) | 32.96 (19.34,54.06) | -0.07 (-0.24 to 0.1) |
| Lao People's Democratic Republic | 1883 (1107,3006) | 39.35 (23.14,62.9) |  | 2250 (1299,3652) | 29.54 (17.21,47.87) | -1.06 (-1.19 to -0.92) |
| Latvia | 747 (453,1184) | 30.48 (18.16,48.18) |  | 403 (242,635) | 25.77 (15.17,40.65) | -0.71 (-0.82 to -0.6) |
| Lebanon | 1036 (603,1649) | 31.66 (18.42,50.55) |  | 1526 (889,2480) | 29.26 (17.01,47.39) | -0.37 (-0.5 to -0.23) |
| Lesotho | 728 (424,1174) | 42.42 (24.95,67.97) |  | 737 (431,1192) | 37 (21.66,58.71) | -0.47 (-0.61 to -0.34) |
| Liberia | 1026 (591,1645) | 48.77 (28.33,78.83) |  | 2874 (1644,4599) | 49.09 (28.84,78.26) | -0.23 (-0.38 to -0.08) |
| Libya | 1275 (741,2053) | 25.5 (14.76,40.99) |  | 1954 (1150,3151) | 29.75 (17.52,47.77) | 0.45 (0.3 to 0.6) |
| Lithuania | 1080 (658,1716) | 31 (18.83,48.83) |  | 558 (327,886) | 24.89 (14.13,39.12) | -0.83 (-0.96 to -0.7) |
| Luxembourg | 65 (38,106) | 18.68 (10.68,30.51) |  | 91 (52,147) | 16.36 (9.24,26.2) | -0.44 (-0.48 to -0.4) |
| Madagascar | 8749 (5348,12871) | 54.82 (33.95,82.93) |  | 13738 (8082,21995) | 43.2 (25.64,69.28) | -0.78 (-0.91 to -0.65) |
| Malawi | 7157 (4002,12426) | 54.61 (31.67,86.25) |  | 9369 (5367,15001) | 44.15 (25.57,69.76) | -0.8 (-0.99 to -0.6) |
| Malaysia | 5628 (3290,9042) | 27.65 (16.2,44.59) |  | 6953 (4002,11121) | 23 (13.13,36.75) | -0.62 (-0.75 to -0.49) |
| Maldives | 85 (49,136) | 30.92 (17.59,48.88) |  | 118 (69,194) | 25.18 (14.6,41.09) | -0.75 (-0.88 to -0.61) |
| Mali | 4771 (2761,7566) | 49.51 (29.06,78.47) |  | 11550 (6732,18347) | 42.95 (25.17,68.59) | -0.53 (-0.66 to -0.39) |
| Malta | 84 (48,137) | 23.21 (13.27,36.92) |  | 73 (43,117) | 19.36 (11.01,30.63) | -0.59 (-0.63 to -0.54) |
| Marshall Islands | 18 (10,29) | 33.64 (19.46,54.57) |  | 19 (11,30) | 32.11 (18.79,51.66) | -0.14 (-0.28 to -0.01) |
| Mauritania | 1000 (574,1601) | 41.96 (24.65,67.45) |  | 1884 (1091,3062) | 38.74 (22.86,63.08) | -0.35 (-0.49 to -0.21) |
| Mauritius | 322 (186,524) | 28.38 (16.45,45.37) |  | 261 (153,421) | 23.81 (13.69,38.54) | -0.55 (-0.68 to -0.42) |
| Mexico | 35036 (22535,53620) | 35.5 (22.86,54.33) |  | 36544 (21406,58603) | 29.22 (16.98,46.79) | -0.55 (-0.68 to -0.42) |
| Micronesia (Federated States of) | 40 (23,63) | 34 (19.77,54.63) |  | 35 (20,56) | 32.68 (19.28,52.62) | -0.12 (-0.25 to 0.01) |
| Monaco | 3 (2,6) | 14.7 (8.34,23.54) |  | 4 (2,6) | 13.43 (7.54,21.99) | -0.33 (-0.35 to -0.31) |
| Mongolia | 813 (471,1317) | 33.68 (19.83,54.87) |  | 936 (547,1514) | 27.06 (15.81,44.01) | -0.92 (-1.05 to -0.79) |
| Montenegro | 170 (99,273) | 27.53 (16.05,44.26) |  | 141 (83,230) | 25.26 (14.76,41.22) | -0.49 (-0.63 to -0.35) |
| Morocco | 10104 (5881,16306) | 35.88 (20.96,57.48) |  | 11794 (6821,19101) | 32.09 (18.59,51.85) | -0.39 (-0.52 to -0.26) |
| Mozambique | 10732 (5932,19966) | 64.74 (36.77,110.86) |  | 15823 (8930,26655) | 44.9 (25.79,71.57) | -1.19 (-1.37 to -1.01) |
| Myanmar | 19130 (10890,30438) | 43.86 (25.42,69.23) |  | 17160 (9783,27696) | 30.36 (17.4,48.87) | -1.48 (-1.62 to -1.34) |
| Namibia | 571 (333,902) | 33.58 (19.88,53.28) |  | 781 (457,1258) | 30.01 (17.52,47.81) | -0.46 (-0.59 to -0.33) |
| Nauru | 3 (2,5) | 24.77 (14.32,40.29) |  | 3 (2,5) | 25.82 (15.05,41.5) | 0.1 (-0.1 to 0.29) |
| Nepal | 12183 (7066,19507) | 55.82 (32.36,88.71) |  | 15145 (8806,24198) | 46.95 (27.5,75.41) | -0.56 (-0.67 to -0.46) |
| Netherlands | 3146 (1950,4881) | 23.05 (14.32,35.16) |  | 2786 (1618,4438) | 18.75 (10.7,29.79) | -0.66 (-0.74 to -0.57) |
| New Zealand | 590 (338,966) | 18.22 (10.37,29.85) |  | 726 (416,1172) | 16.62 (9.44,26.81) | -0.35 (-0.36 to -0.33) |
| Nicaragua | 1666 (978,2679) | 36.34 (21.5,57.79) |  | 2265 (1340,3658) | 33.33 (19.71,53.63) | -0.37 (-0.47 to -0.26) |
| Niger | 4631 (2694,7476) | 50.5 (29.95,80.82) |  | 13697 (7722,22002) | 50.21 (29.33,80.54) | -0.06 (-0.2 to 0.08) |
| Nigeria | 46589 (26941,74606) | 43.65 (25.41,70.34) |  | 97767 (56803,158107) | 37.11 (21.69,59.9) | -0.9 (-1.04 to -0.76) |
| Niue | 1 (0,1) | 28.39 (16.68,45.75) |  | 0 (0,1) | 25.59 (14.85,41.15) | -0.45 (-0.57 to -0.32) |
| North Macedonia | 614 (375,969) | 31.73 (19.34,49.93) |  | 510 (299,830) | 26.31 (15.1,42.31) | -0.63 (-0.77 to -0.48) |
| Northern Mariana Islands | 10 (6,16) | 20.66 (12.03,32.62) |  | 10 (6,16) | 21.66 (12.41,35) | 0.33 (0.2 to 0.46) |
| Norway | 866 (517,1368) | 22.46 (13.21,35.53) |  | 914 (532,1454) | 19.79 (11.24,31.79) | -0.43 (-0.48 to -0.38) |
| Oman | 603 (342,975) | 25.59 (14.68,40.91) |  | 1090 (628,1742) | 23.73 (13.7,37.6) | -0.36 (-0.49 to -0.23) |
| Pakistan | 61187 (36123,97445) | 46.89 (27.59,74.98) |  | 108384 (63407,175026) | 42.1 (24.74,67.88) | -0.39 (-0.47 to -0.31) |
| Palau | 4 (2,7) | 26.38 (15.41,42.7) |  | 4 (2,7) | 24.62 (14.3,39.76) | -0.24 (-0.37 to -0.11) |
| Palestine | 962 (558,1541) | 39.16 (22.97,61.83) |  | 1975 (1123,3184) | 35.85 (20.72,57.11) | -0.26 (-0.4 to -0.13) |
| Panama | 819 (481,1323) | 31.17 (18.52,49.51) |  | 1097 (642,1756) | 25.96 (15.15,41.52) | -0.68 (-0.78 to -0.58) |
| Papua New Guinea | 1612 (952,2603) | 34.16 (20.09,55.17) |  | 3559 (2040,5794) | 31.8 (18.43,51.47) | -0.18 (-0.32 to -0.04) |
| Paraguay | 1432 (835,2249) | 30.7 (17.86,49.04) |  | 1972 (1155,3159) | 27.59 (16.09,43.96) | -0.38 (-0.48 to -0.27) |
| Peru | 7292 (4197,11757) | 30.76 (17.98,49.96) |  | 9507 (5535,15309) | 26.54 (15.53,42.71) | -0.63 (-0.72 to -0.53) |
| Philippines | 26052 (15532,41800) | 35.66 (21.17,57.05) |  | 36155 (21199,58349) | 31.13 (18.17,50.32) | -0.45 (-0.58 to -0.32) |
| Poland | 11936 (7472,18651) | 33.24 (21.13,51.57) |  | 7989 (4712,13027) | 24.66 (14.36,40.1) | -0.89 (-1.04 to -0.74) |
| Portugal | 2636 (1723,3913) | 30.38 (20.27,44.77) |  | 1868 (1115,2983) | 20.41 (11.86,32.25) | -1.15 (-1.32 to -0.98) |
| Puerto Rico | 910 (523,1442) | 25.25 (14.57,40.04) |  | 636 (380,1008) | 23.85 (13.95,37.76) | -0.25 (-0.36 to -0.13) |
| Qatar | 112 (65,183) | 24.05 (13.96,38.66) |  | 526 (306,852) | 21.56 (12.5,34.98) | -0.53 (-0.66 to -0.39) |
| Republic of Korea | 10556 (6250,17226) | 23.71 (14.06,38.4) |  | 7899 (4521,12651) | 18.28 (10.53,29.35) | -0.86 (-0.95 to -0.77) |
| Republic of Moldova | 1618 (995,2546) | 36.83 (22.57,57.7) |  | 1023 (609,1649) | 32.71 (19.37,52.64) | -0.49 (-0.62 to -0.36) |
| Romania | 7545 (4896,11434) | 37.01 (24.6,55) |  | 3967 (2323,6380) | 24.19 (14.02,39.2) | -1.28 (-1.45 to -1.12) |
| Russian Federation | 41571 (24427,66911) | 29.32 (17.16,46.99) |  | 34434 (20292,55299) | 27.67 (16.12,44.7) | -0.4 (-0.54 to -0.27) |
| Rwanda | 4738 (2729,8107) | 53.56 (31.6,86.36) |  | 5463 (3134,8883) | 38.49 (22.21,61.63) | -1.42 (-1.58 to -1.26) |
| Saint Kitts and Nevis | 12 (7,19) | 27.64 (15.92,44.32) |  | 13 (7,20) | 24.51 (14.33,39.19) | -0.4 (-0.5 to -0.3) |
| Saint Lucia | 45 (26,72) | 29.97 (17.51,47.23) |  | 45 (27,72) | 27.94 (16.62,44.6) | -0.24 (-0.35 to -0.14) |
| Saint Vincent and the Grenadines | 37 (22,58) | 31.72 (18.84,50.15) |  | 31 (18,50) | 28.62 (16.59,45.98) | -0.37 (-0.48 to -0.27) |
| Samoa | 60 (35,97) | 32.28 (18.94,52.16) |  | 67 (39,110) | 29.3 (17.1,48.12) | -0.38 (-0.51 to -0.25) |
| San Marino | 4 (2,6) | 19.35 (11.18,30.63) |  | 5 (3,8) | 18.06 (10.19,28.94) | -0.22 (-0.29 to -0.15) |
| Sao Tome and Principe | 61 (35,98) | 43.97 (25.32,70.97) |  | 92 (53,147) | 39.51 (22.88,63.05) | -0.47 (-0.61 to -0.33) |
| Saudi Arabia | 4916 (2818,7746) | 25.2 (14.51,40.35) |  | 8185 (4723,13274) | 23.87 (13.63,38.2) | -0.29 (-0.42 to -0.16) |
| Senegal | 3777 (2177,6023) | 42.13 (24.37,67.77) |  | 6898 (3995,11007) | 39.77 (23.34,63.46) | -0.23 (-0.37 to -0.08) |
| Serbia | 2588 (1531,4131) | 27.99 (16.57,45.33) |  | 2004 (1195,3257) | 25.76 (14.96,41.85) | -0.49 (-0.63 to -0.35) |
| Seychelles | 20 (12,32) | 26.11 (15.25,41.61) |  | 22 (13,36) | 22.94 (13.24,36.57) | -0.41 (-0.54 to -0.28) |
| Sierra Leone | 2043 (1191,3239) | 45.61 (26.73,73.26) |  | 4433 (2534,7094) | 46.36 (27.48,74.08) | -0.03 (-0.18 to 0.13) |
| Singapore | 618 (363,999) | 20.32 (11.88,32.58) |  | 771 (451,1257) | 16.08 (9.19,25.47) | -0.81 (-0.86 to -0.76) |
| Slovakia | 1369 (805,2173) | 26.97 (15.85,42.86) |  | 1068 (641,1707) | 22.82 (13.37,37.16) | -0.66 (-0.8 to -0.52) |
| Slovenia | 496 (305,778) | 28.25 (17.8,43.74) |  | 395 (227,635) | 22.41 (12.75,36.36) | -0.67 (-0.81 to -0.52) |
| Solomon Islands | 144 (83,233) | 36.58 (21.25,59.33) |  | 257 (149,417) | 35.33 (20.67,57.55) | -0.05 (-0.2 to 0.09) |
| Somalia | 6640 (3587,15020) | 73.29 (39.85,142.87) |  | 16838 (9020,29635) | 71.44 (39.86,118.04) | -0.02 (-0.2 to 0.16) |
| South Africa | 14697 (8812,23449) | 34.65 (20.85,54.66) |  | 18105 (10640,28762) | 32.32 (19.09,51.4) | -0.3 (-0.44 to -0.16) |
| South Sudan | 3086 (1748,5701) | 40.17 (22.57,69.58) |  | 4933 (2727,8792) | 43.42 (24.86,71.2) | 0.22 (0.04 to 0.4) |
| Spain | 8386 (4936,13273) | 24.08 (14.07,38.7) |  | 8038 (4677,13051) | 20.89 (11.7,33.02) | -0.46 (-0.52 to -0.41) |
| Sri Lanka | 6150 (3522,10127) | 34.05 (19.87,55.59) |  | 5552 (3228,8883) | 26.47 (15.42,42.17) | -0.91 (-1.04 to -0.78) |
| Sudan | 9708 (5576,15655) | 41.62 (24.26,66.36) |  | 17119 (9724,27172) | 36.52 (21.27,58.1) | -0.57 (-0.71 to -0.43) |
| Suriname | 120 (69,193) | 29.39 (17.23,47.17) |  | 153 (89,245) | 27.28 (15.7,43.25) | -0.4 (-0.51 to -0.29) |
| Sweden | 1756 (1034,2798) | 22.18 (12.76,35.44) |  | 1810 (1065,2896) | 19.93 (11.52,31.66) | -0.43 (-0.46 to -0.39) |
| Switzerland | 1241 (727,1961) | 19.97 (11.6,31.18) |  | 1345 (774,2138) | 17.54 (9.9,27.73) | -0.45 (-0.5 to -0.4) |
| Syrian Arab Republic | 5597 (3263,8918) | 37 (21.76,58.5) |  | 5046 (2977,7946) | 35.73 (20.79,57.27) | -0.19 (-0.33 to -0.05) |
| Taiwan (Province of China) | 4588 (2655,7391) | 22.45 (12.95,35.85) |  | 3662 (2136,5835) | 18.63 (10.76,29.87) | -0.61 (-0.74 to -0.49) |
| Tajikistan | 2009 (1174,3271) | 33.03 (19.48,52.81) |  | 3678 (2127,5965) | 34.67 (20.18,55.18) | -0.1 (-0.29 to 0.09) |
| Thailand | 18301 (10586,29764) | 30.87 (18,49.18) |  | 14799 (8689,23780) | 25.95 (14.9,41.52) | -0.56 (-0.7 to -0.43) |
| Timor-Leste | 320 (183,514) | 35.67 (20.61,57.7) |  | 456 (265,736) | 30.13 (17.51,48.84) | -0.81 (-0.96 to -0.67) |
| Togo | 1982 (1135,3143) | 46.48 (27.17,74.3) |  | 4078 (2392,6552) | 45.34 (26.92,71.53) | -0.06 (-0.21 to 0.08) |
| Tokelau | 1 (0,1) | 31.97 (18.42,51.77) |  | 0 (0,1) | 28.24 (16.15,45.69) | -0.5 (-0.63 to -0.38) |
| Tonga | 35 (20,56) | 31.88 (18.5,51.22) |  | 33 (19,54) | 29.28 (16.98,47.64) | -0.26 (-0.39 to -0.13) |
| Trinidad and Tobago | 358 (205,585) | 28.17 (16.16,45.66) |  | 294 (170,469) | 23.74 (13.5,38.22) | -0.79 (-0.91 to -0.68) |
| Tunisia | 3188 (1847,5106) | 34.27 (20.12,54.35) |  | 3401 (1985,5502) | 30.21 (17.71,48.59) | -0.43 (-0.56 to -0.3) |
| Turkey | 19535 (11367,31359) | 30.96 (17.96,49.12) |  | 20669 (12213,32582) | 26.32 (15.46,41.86) | -0.62 (-0.76 to -0.49) |
| Turkmenistan | 1268 (746,2023) | 30.3 (17.99,48.85) |  | 1300 (747,2103) | 24.79 (14.27,40.1) | -0.98 (-1.14 to -0.83) |
| Tuvalu | 4 (2,6) | 35.42 (20.96,57.5) |  | 4 (2,7) | 31.97 (18.64,51.47) | -0.27 (-0.4 to -0.13) |
| Uganda | 10860 (5811,20117) | 48.38 (27.74,79.57) |  | 18457 (10450,30075) | 37.52 (21.7,60.44) | -0.97 (-1.13 to -0.8) |
| Ukraine | 15221 (8968,24452) | 31.4 (18.58,50.11) |  | 11621 (6922,18963) | 31.98 (18.8,50.86) | -0.13 (-0.26 to 0.01) |
| United Arab Emirates | 447 (258,722) | 20.89 (12.05,33.11) |  | 1806 (1024,2914) | 22.73 (13.06,36.97) | 0.33 (0.21 to 0.46) |
| United Kingdom | 12839 (7853,19978) | 24.38 (14.78,37.76) |  | 12452 (7389,19743) | 20.71 (12.01,32.93) | -0.47 (-0.54 to -0.39) |
| United Republic of Tanzania | 15357 (8325,25489) | 46.34 (26.41,74.14) |  | 23712 (13316,38981) | 36.16 (20.64,59.7) | -0.89 (-1.04 to -0.75) |
| United States of America | 47344 (28930,74991) | 19.82 (11.87,31.59) |  | 51426 (30947,82322) | 18.11 (10.53,29.13) | -0.33 (-0.43 to -0.24) |
| United States Virgin Islands | 27 (15,43) | 24.48 (14.12,39.98) |  | 16 (9,25) | 22.4 (12.71,35.57) | -0.37 (-0.48 to -0.26) |
| Uruguay | 722 (419,1133) | 23.47 (13.59,36.93) |  | 619 (357,990) | 20.32 (11.71,32.46) | -0.53 (-0.58 to -0.48) |
| Uzbekistan | 7810 (4501,12796) | 33.21 (19.1,54.32) |  | 10016 (5709,16294) | 28.94 (16.57,46.99) | -0.6 (-0.74 to -0.46) |
| Vanuatu | 61 (35,98) | 34.83 (20.22,55.6) |  | 112 (66,184) | 33.62 (19.97,55.16) | -0.11 (-0.24 to 0.03) |
| Venezuela (Bolivarian Republic of) | 6285 (3713,9980) | 29.35 (17.46,46.87) |  | 7461 (4368,11974) | 29 (17.03,46.52) | -0.19 (-0.3 to -0.08) |
| Viet Nam | 28495 (16535,45509) | 37.83 (22.08,60.25) |  | 27653 (15957,44970) | 28.53 (16.34,46.13) | -0.99 (-1.12 to -0.87) |
| Yemen | 6439 (3749,10170) | 39.35 (23.12,62.13) |  | 14653 (8443,23317) | 40.48 (23.81,64.11) | -0.07 (-0.23 to 0.08) |
| Zambia | 4645 (2602,7830) | 45.06 (26.06,72.49) |  | 7536 (4292,12209) | 34.13 (19.58,54.87) | -1.17 (-1.31 to -1.02) |
| Zimbabwe | 4441 (2576,7123) | 36.04 (21.05,58.59) |  | 6614 (3777,10636) | 38.87 (22.33,61.98) | 0.46 (0.32 to 0.6) |
| DALYs, disability-adjusted life years. ASDR, age-standardized DALYs rate. EAPC, estimated annual percentage changes. | | | | | | |

| **Table S4** APC model analysis of incidence. | | | | | | | | |
| --- | --- | --- | --- | --- | --- | --- | --- | --- |
| **Group** | **Variable** | **Coef** | **SE** | **Z** | **P** | **RR** | **95%CI** | |
|  |  |  |  |  |  |  | **Lower** | **Upper** |
| **Age** |  |  |  |  |  |  |  |  |
|  | <5 | 2.262 | 0.001 | 4262.820 | <0.001 | 9.606 | 9.596 | 9.616 |
|  | 5-9 | 1.620 | 0.000 | 3434.820 | <0.001 | 5.051 | 5.046 | 5.055 |
|  | 10-14 | 0.719 | 0.000 | 1735.610 | <0.001 | 2.053 | 2.052 | 2.055 |
|  | 15-19 | 0.227 | 0.000 | 629.520 | <0.001 | 1.254 | 1.253 | 1.255 |
|  | 20-24 | -0.247 | 0.000 | -794.990 | <0.001 | 0.781 | 0.781 | 0.782 |
|  | 25-29 | -0.388 | 0.000 | -1464.490 | <0.001 | 0.678 | 0.678 | 0.679 |
|  | 30-34 | -0.348 | 0.000 | -1547.320 | <0.001 | 0.706 | 0.706 | 0.707 |
|  | 35-39 | -0.125 | 0.000 | -649.120 | <0.001 | 0.883 | 0.882 | 0.883 |
|  | 40-44 | -0.101 | 0.000 | -554.740 | <0.001 | 0.904 | 0.904 | 0.905 |
|  | 45-49 | -0.285 | 0.000 | -1440.220 | <0.001 | 0.752 | 0.752 | 0.752 |
|  | 50-54 | -0.312 | 0.000 | -1371.460 | <0.001 | 0.732 | 0.731 | 0.732 |
|  | 55-59 | -0.149 | 0.000 | -568.740 | <0.001 | 0.862 | 0.861 | 0.862 |
|  | 60-64 | -0.117 | 0.000 | -380.300 | <0.001 | 0.890 | 0.889 | 0.890 |
|  | 65-69 | -0.224 | 0.000 | -617.500 | <0.001 | 0.799 | 0.799 | 0.800 |
|  | 70-74 | -0.307 | 0.000 | -722.220 | <0.001 | 0.736 | 0.735 | 0.736 |
|  | 75-79 | -0.352 | 0.000 | -711.630 | <0.001 | 0.703 | 0.702 | 0.704 |
|  | 80-84 | -0.425 | 0.001 | -720.100 | <0.001 | 0.654 | 0.653 | 0.655 |
|  | 85-89 | -0.536 | 0.001 | -705.670 | <0.001 | 0.585 | 0.584 | 0.586 |
|  | 90-94 | -0.529 | 0.001 | -481.320 | <0.001 | 0.589 | 0.588 | 0.590 |
|  | 95+ | -0.384 | 0.002 | -204.890 | <0.001 | 0.681 | 0.679 | 0.684 |
| **Period** |  |  |  |  |  |  |  |  |
|  | 1992~1996 | 0.105 | 0.000 | 655.690 | <0.001 | 1.111 | 1.111 | 1.111 |
|  | 1997~2001 | 0.067 | 0.000 | 656.370 | <0.001 | 1.069 | 1.069 | 1.069 |
|  | 2002~2006 | 0.025 | 0.000 | 488.840 | <0.001 | 1.025 | 1.025 | 1.025 |
|  | 2007~2011 | -0.020 | 0.000 | -395.550 | <0.001 | 0.980 | 0.980 | 0.980 |
|  | 2012~2016 | -0.066 | 0.000 | -652.180 | <0.001 | 0.936 | 0.936 | 0.936 |
|  | 2017~2021 | -0.111 | 0.000 | -686.990 | <0.001 | 0.895 | 0.895 | 0.895 |
| **Birth cohort** |  |  |  |  |  |  |  |  |
|  | 1897-1901 | -0.579 | 0.007 | -85.430 | <0.001 | 0.560 | 0.553 | 0.568 |
|  | 1902-1906 | -0.502 | 0.003 | -166.260 | <0.001 | 0.605 | 0.602 | 0.609 |
|  | 1907-1911 | -0.441 | 0.002 | -256.730 | <0.001 | 0.643 | 0.641 | 0.645 |
|  | 1912-1916 | -0.392 | 0.001 | -321.580 | <0.001 | 0.676 | 0.674 | 0.677 |
|  | 1917-1921 | -0.355 | 0.001 | -353.010 | <0.001 | 0.701 | 0.700 | 0.703 |
|  | 1922-1926 | -0.310 | 0.001 | -357.610 | <0.001 | 0.734 | 0.732 | 0.735 |
|  | 1927-1931 | -0.255 | 0.001 | -326.800 | <0.001 | 0.775 | 0.774 | 0.776 |
|  | 1932-1936 | -0.218 | 0.001 | -307.750 | <0.001 | 0.804 | 0.803 | 0.805 |
|  | 1937-1941 | -0.176 | 0.001 | -273.150 | <0.001 | 0.839 | 0.838 | 0.840 |
|  | 1942-1946 | -0.144 | 0.001 | -247.410 | <0.001 | 0.866 | 0.865 | 0.867 |
|  | 1947-1951 | -0.105 | 0.001 | -201.650 | <0.001 | 0.900 | 0.899 | 0.901 |
|  | 1952-1956 | -0.058 | 0.000 | -127.880 | <0.001 | 0.943 | 0.942 | 0.944 |
|  | 1957-1961 | -0.015 | 0.000 | -37.520 | <0.001 | 0.985 | 0.984 | 0.986 |
|  | 1962-1966 | 0.032 | 0.000 | 94.620 | <0.001 | 1.032 | 1.032 | 1.033 |
|  | 1967-1971 | 0.080 | 0.000 | 287.240 | <0.001 | 1.083 | 1.083 | 1.084 |
|  | 1972-1976 | 0.129 | 0.000 | 580.640 | <0.001 | 1.137 | 1.137 | 1.138 |
|  | 1977-1981 | 0.176 | 0.000 | 1066.090 | <0.001 | 1.193 | 1.192 | 1.193 |
|  | 1982-1986 | 0.222 | 0.000 | 1965.490 | <0.001 | 1.249 | 1.249 | 1.249 |
|  | 1987-1991 | 0.262 | 0.000 | 3366.140 | <0.001 | 1.300 | 1.299 | 1.300 |
|  | 1992-1996 | 0.313 | 0.000 | 3412.410 | <0.001 | 1.367 | 1.367 | 1.367 |
|  | 1997-2001 | 0.371 | 0.000 | 2611.780 | <0.001 | 1.449 | 1.449 | 1.449 |
|  | 2002-2006 | 0.424 | 0.000 | 2130.680 | <0.001 | 1.528 | 1.528 | 1.529 |
|  | 2007-2011 | 0.471 | 0.000 | 1821.330 | <0.001 | 1.602 | 1.601 | 1.602 |
|  | 2012-2016 | 0.515 | 0.000 | 1611.650 | <0.001 | 1.674 | 1.673 | 1.676 |
|  | 2017-2021 | 0.555 | 0.000 | 1442.780 | <0.001 | 1.742 | 1.741 | 1.743 |
| APC, age-period-cohort. SE, standard error. RR, relative risk. CI, confidence interval. | | | | | | | | |

| **Table S5** APC model analysis of prevalence. | | | | | | | | |
| --- | --- | --- | --- | --- | --- | --- | --- | --- |
| **Group** | **Variable** | **Coef** | **SE** | **Z** | **P** | **RR** | **95%CI** | |
|  |  |  |  |  |  |  | **Lower** | **Upper** |
| **Age** |  |  |  |  |  |  |  |  |
|  | <5 | 0.595 | 0.001 | 1160.470 | <0.001 | 1.813 | 1.811 | 1.815 |
|  | 5-9 | 0.638 | 0.000 | 1404.220 | <0.001 | 1.893 | 1.891 | 1.894 |
|  | 10-14 | 0.407 | 0.000 | 1018.920 | <0.001 | 1.503 | 1.502 | 1.504 |
|  | 15-19 | 0.404 | 0.000 | 1165.950 | <0.001 | 1.498 | 1.497 | 1.499 |
|  | 20-24 | 0.279 | 0.000 | 938.000 | <0.001 | 1.322 | 1.321 | 1.323 |
|  | 25-29 | 0.073 | 0.000 | 285.870 | <0.001 | 1.076 | 1.075 | 1.077 |
|  | 30-34 | -0.061 | 0.000 | -271.130 | <0.001 | 0.941 | 0.941 | 0.942 |
|  | 35-39 | -0.076 | 0.000 | -374.950 | <0.001 | 0.927 | 0.926 | 0.927 |
|  | 40-44 | -0.029 | 0.000 | -150.060 | <0.001 | 0.971 | 0.971 | 0.971 |
|  | 45-49 | 0.052 | 0.000 | 252.390 | <0.001 | 1.054 | 1.053 | 1.054 |
|  | 50-54 | 0.112 | 0.000 | 480.400 | <0.001 | 1.119 | 1.118 | 1.119 |
|  | 55-59 | 0.014 | 0.000 | 49.060 | <0.001 | 1.014 | 1.013 | 1.014 |
|  | 60-64 | -0.138 | 0.000 | -422.820 | <0.001 | 0.871 | 0.871 | 0.872 |
|  | 65-69 | -0.287 | 0.000 | -750.010 | <0.001 | 0.751 | 0.750 | 0.751 |
|  | 70-74 | -0.392 | 0.000 | -880.370 | <0.001 | 0.676 | 0.675 | 0.676 |
|  | 75-79 | -0.448 | 0.001 | -862.850 | <0.001 | 0.639 | 0.638 | 0.639 |
|  | 80-84 | -0.447 | 0.001 | -726.690 | <0.001 | 0.639 | 0.639 | 0.640 |
|  | 85-89 | -0.356 | 0.001 | -466.540 | <0.001 | 0.700 | 0.699 | 0.701 |
|  | 90-94 | -0.232 | 0.001 | -216.810 | <0.001 | 0.793 | 0.791 | 0.795 |
|  | 95+ | -0.108 | 0.002 | -58.630 | <0.001 | 0.898 | 0.894 | 0.901 |
| **Period** |  |  |  |  |  |  |  |  |
|  | 1992~1996 | 0.133 | 0.000 | 827.420 | <0.001 | 1.143 | 1.142 | 1.143 |
|  | 1997~2001 | 0.088 | 0.000 | 801.450 | <0.001 | 1.092 | 1.092 | 1.092 |
|  | 2002~2006 | 0.039 | 0.000 | 553.510 | <0.001 | 1.039 | 1.039 | 1.040 |
|  | 2007~2011 | -0.022 | 0.000 | -313.390 | <0.001 | 0.979 | 0.979 | 0.979 |
|  | 2012~2016 | -0.088 | 0.000 | -805.680 | <0.001 | 0.916 | 0.916 | 0.916 |
|  | 2017~2021 | -0.151 | 0.000 | -925.070 | <0.001 | 0.860 | 0.859 | 0.860 |
| **Birth cohort** |  |  |  |  |  |  |  |  |
|  | 1897-1901 | -0.388 | 0.006 | -60.520 | <0.001 | 0.678 | 0.670 | 0.687 |
|  | 1902-1906 | -0.338 | 0.003 | -117.530 | <0.001 | 0.713 | 0.709 | 0.718 |
|  | 1907-1911 | -0.291 | 0.002 | -172.530 | <0.001 | 0.747 | 0.745 | 0.750 |
|  | 1912-1916 | -0.249 | 0.001 | -202.430 | <0.001 | 0.779 | 0.778 | 0.781 |
|  | 1917-1921 | -0.196 | 0.001 | -193.520 | <0.001 | 0.822 | 0.821 | 0.824 |
|  | 1922-1926 | -0.165 | 0.001 | -190.860 | <0.001 | 0.848 | 0.847 | 0.850 |
|  | 1927-1931 | -0.123 | 0.001 | -159.720 | <0.001 | 0.884 | 0.883 | 0.885 |
|  | 1932-1936 | -0.083 | 0.001 | -118.400 | <0.001 | 0.921 | 0.919 | 0.922 |
|  | 1937-1941 | -0.056 | 0.001 | -87.750 | <0.001 | 0.946 | 0.945 | 0.947 |
|  | 1942-1946 | -0.038 | 0.001 | -65.480 | <0.001 | 0.963 | 0.962 | 0.964 |
|  | 1947-1951 | -0.028 | 0.001 | -55.200 | <0.001 | 0.972 | 0.971 | 0.973 |
|  | 1952-1956 | -0.011 | 0.000 | -23.420 | <0.001 | 0.989 | 0.989 | 0.990 |
|  | 1957-1961 | 0.004 | 0.000 | 9.550 | <0.001 | 1.004 | 1.003 | 1.005 |
|  | 1962-1966 | 0.020 | 0.000 | 60.840 | <0.001 | 1.021 | 1.020 | 1.021 |
|  | 1967-1971 | 0.041 | 0.000 | 149.520 | <0.001 | 1.042 | 1.042 | 1.043 |
|  | 1972-1976 | 0.055 | 0.000 | 248.780 | <0.001 | 1.057 | 1.056 | 1.057 |
|  | 1977-1981 | 0.059 | 0.000 | 344.420 | <0.001 | 1.061 | 1.060 | 1.061 |
|  | 1982-1986 | 0.067 | 0.000 | 527.070 | <0.001 | 1.069 | 1.069 | 1.070 |
|  | 1987-1991 | 0.085 | 0.000 | 849.280 | <0.001 | 1.089 | 1.089 | 1.089 |
|  | 1992-1996 | 0.126 | 0.000 | 1117.880 | <0.001 | 1.134 | 1.134 | 1.134 |
|  | 1997-2001 | 0.182 | 0.000 | 1158.880 | <0.001 | 1.199 | 1.199 | 1.200 |
|  | 2002-2006 | 0.243 | 0.000 | 1154.830 | <0.001 | 1.275 | 1.274 | 1.276 |
|  | 2007-2011 | 0.300 | 0.000 | 1115.800 | <0.001 | 1.350 | 1.349 | 1.350 |
|  | 2012-2016 | 0.360 | 0.000 | 1086.140 | <0.001 | 1.433 | 1.432 | 1.434 |
|  | 2017-2021 | 0.423 | 0.000 | 1023.210 | <0.001 | 1.526 | 1.525 | 1.527 |
| APC, age-period-cohort. SE, standard error. RR, relative risk. CI, confidence interval. | | | | | | | | |

| **Table S6** APC model analysis of DALYs. | | | | | | | | |
| --- | --- | --- | --- | --- | --- | --- | --- | --- |
| **Group** | **Variable** | **Coef** | **SE** | **Z** | **P** | **RR** | **95%CI** | |
|  |  |  |  |  |  |  | **Lower** | **Upper** |
| **Age** |  |  |  |  |  |  |  |  |
|  | <5 | 0.529 | 0.003 | 151.600 | <0.001 | 1.697 | 1.686 | 1.709 |
|  | 5-9 | 0.607 | 0.003 | 196.420 | <0.001 | 1.835 | 1.823 | 1.846 |
|  | 10-14 | 0.436 | 0.003 | 160.660 | <0.001 | 1.547 | 1.539 | 1.555 |
|  | 15-19 | 0.413 | 0.002 | 175.660 | <0.001 | 1.512 | 1.505 | 1.519 |
|  | 20-24 | 0.292 | 0.002 | 144.540 | <0.001 | 1.339 | 1.334 | 1.345 |
|  | 25-29 | 0.088 | 0.002 | 50.470 | <0.001 | 1.092 | 1.088 | 1.096 |
|  | 30-34 | -0.047 | 0.002 | -30.850 | <0.001 | 0.954 | 0.951 | 0.957 |
|  | 35-39 | -0.065 | 0.001 | -46.340 | <0.001 | 0.937 | 0.935 | 0.940 |
|  | 40-44 | -0.015 | 0.001 | -11.270 | <0.001 | 0.985 | 0.982 | 0.987 |
|  | 45-49 | 0.071 | 0.001 | 48.980 | <0.001 | 1.073 | 1.070 | 1.076 |
|  | 50-54 | 0.132 | 0.002 | 80.720 | <0.001 | 1.141 | 1.137 | 1.145 |
|  | 55-59 | 0.033 | 0.002 | 17.360 | <0.001 | 1.034 | 1.030 | 1.038 |
|  | 60-64 | -0.120 | 0.002 | -52.670 | <0.001 | 0.887 | 0.883 | 0.891 |
|  | 65-69 | -0.273 | 0.003 | -102.500 | <0.001 | 0.761 | 0.757 | 0.765 |
|  | 70-74 | -0.380 | 0.003 | -122.700 | <0.001 | 0.684 | 0.680 | 0.688 |
|  | 75-79 | -0.447 | 0.004 | -123.670 | <0.001 | 0.639 | 0.635 | 0.644 |
|  | 80-84 | -0.458 | 0.004 | -106.610 | <0.001 | 0.633 | 0.628 | 0.638 |
|  | 85-89 | -0.382 | 0.005 | -71.440 | <0.001 | 0.682 | 0.675 | 0.690 |
|  | 90-94 | -0.277 | 0.008 | -36.590 | <0.001 | 0.758 | 0.747 | 0.769 |
|  | 95+ | -0.137 | 0.013 | -10.540 | <0.001 | 0.872 | 0.850 | 0.895 |
| **Period** |  |  |  |  |  |  |  |  |
|  | 1992~1996 | 0.156 | 0.001 | 140.870 | <0.001 | 1.168 | 1.166 | 1.171 |
|  | 1997~2001 | 0.094 | 0.001 | 123.820 | <0.001 | 1.098 | 1.097 | 1.100 |
|  | 2002~2006 | 0.036 | 0.000 | 74.980 | <0.001 | 1.037 | 1.036 | 1.038 |
|  | 2007~2011 | -0.028 | 0.000 | -59.440 | <0.001 | 0.972 | 0.971 | 0.973 |
|  | 2012~2016 | -0.097 | 0.001 | -129.220 | <0.001 | 0.908 | 0.907 | 0.909 |
|  | 2017~2021 | -0.161 | 0.001 | -143.300 | <0.001 | 0.851 | 0.849 | 0.853 |
| **Birth cohort** | |  |  |  |  |  |  |  |
|  | 1897-1901 | -0.320 | 0.043 | -7.370 | <0.001 | 0.726 | 0.667 | 0.791 |
|  | 1902-1906 | -0.314 | 0.020 | -15.730 | <0.001 | 0.731 | 0.703 | 0.760 |
|  | 1907-1911 | -0.281 | 0.012 | -24.010 | <0.001 | 0.755 | 0.738 | 0.773 |
|  | 1912-1916 | -0.250 | 0.009 | -29.340 | <0.001 | 0.779 | 0.766 | 0.792 |
|  | 1917-1921 | -0.199 | 0.007 | -28.610 | <0.001 | 0.819 | 0.808 | 0.831 |
|  | 1922-1926 | -0.169 | 0.006 | -28.510 | <0.001 | 0.845 | 0.835 | 0.854 |
|  | 1927-1931 | -0.132 | 0.005 | -24.940 | <0.001 | 0.876 | 0.867 | 0.886 |
|  | 1932-1936 | -0.096 | 0.005 | -20.100 | <0.001 | 0.908 | 0.900 | 0.917 |
|  | 1937-1941 | -0.076 | 0.004 | -17.380 | <0.001 | 0.927 | 0.919 | 0.935 |
|  | 1942-1946 | -0.063 | 0.004 | -15.970 | <0.001 | 0.939 | 0.932 | 0.946 |
|  | 1947-1951 | -0.055 | 0.004 | -15.650 | <0.001 | 0.947 | 0.940 | 0.953 |
|  | 1952-1956 | -0.034 | 0.003 | -11.100 | <0.001 | 0.966 | 0.960 | 0.972 |
|  | 1957-1961 | -0.016 | 0.003 | -6.020 | <0.001 | 0.984 | 0.979 | 0.989 |
|  | 1962-1966 | 0.004 | 0.002 | 1.740 | 0.082 | 1.004 | 0.999 | 1.009 |
|  | 1967-1971 | 0.029 | 0.002 | 15.490 | <0.001 | 1.030 | 1.026 | 1.034 |
|  | 1972-1976 | 0.048 | 0.002 | 31.220 | <0.001 | 1.049 | 1.046 | 1.052 |
|  | 1977-1981 | 0.056 | 0.001 | 47.170 | <0.001 | 1.057 | 1.055 | 1.060 |
|  | 1982-1986 | 0.069 | 0.001 | 78.310 | <0.001 | 1.071 | 1.069 | 1.073 |
|  | 1987-1991 | 0.090 | 0.001 | 129.120 | <0.001 | 1.094 | 1.093 | 1.096 |
|  | 1992-1996 | 0.144 | 0.001 | 185.100 | <0.001 | 1.155 | 1.154 | 1.157 |
|  | 1997-2001 | 0.201 | 0.001 | 185.070 | <0.001 | 1.223 | 1.220 | 1.225 |
|  | 2002-2006 | 0.264 | 0.001 | 181.780 | <0.001 | 1.302 | 1.299 | 1.306 |
|  | 2007-2011 | 0.319 | 0.002 | 172.260 | <0.001 | 1.376 | 1.371 | 1.381 |
|  | 2012-2016 | 0.372 | 0.002 | 162.800 | <0.001 | 1.451 | 1.444 | 1.457 |
|  | 2017-2021 | 0.409 | 0.003 | 142.530 | <0.001 | 1.505 | 1.496 | 1.513 |
| DALYs, disability-adjusted life years. APC, age-period-cohort. SE, standard error. RR, relative risk. CI, confidence interval. | | | | | | | | |


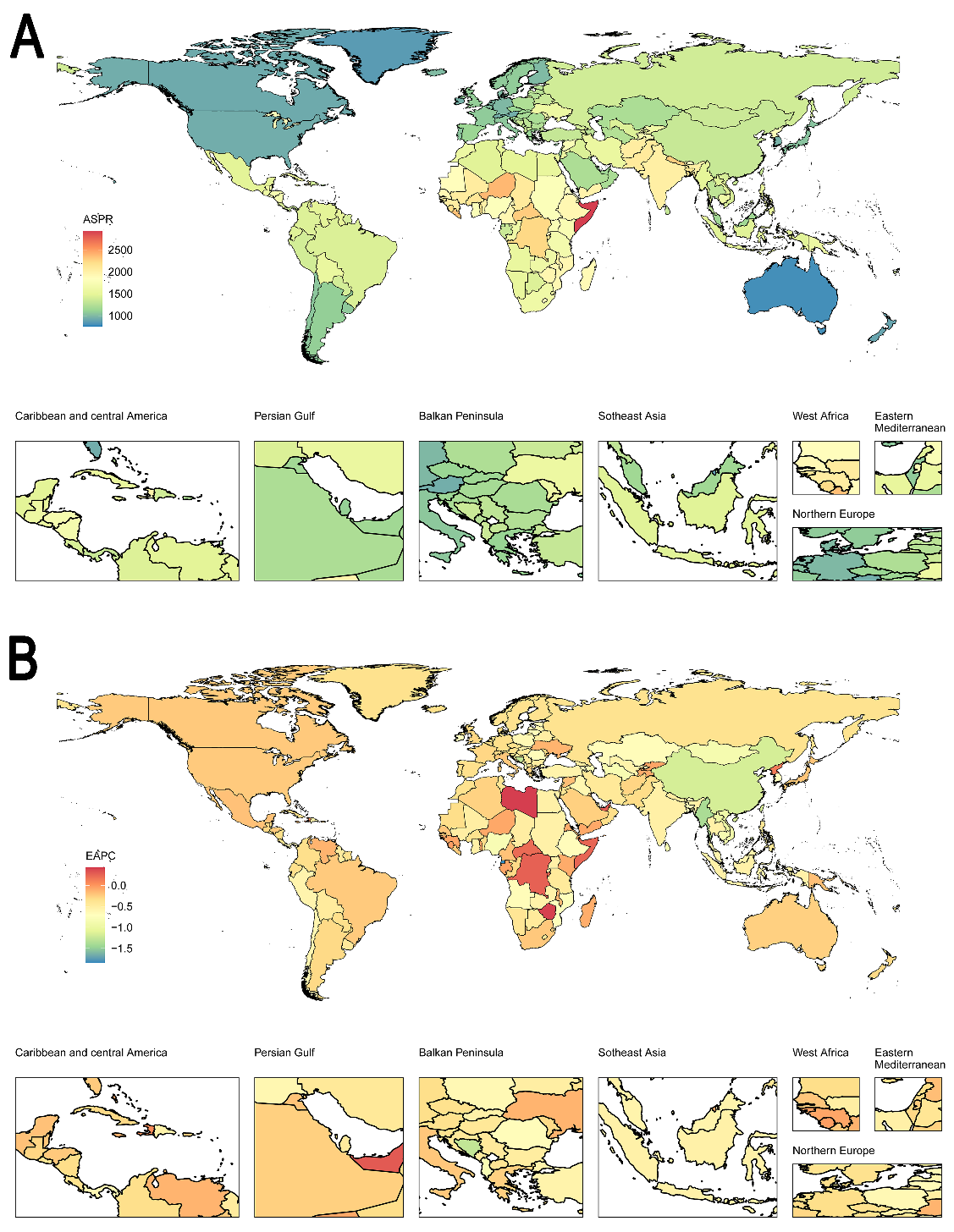


**Figure S1** Global distribution in ASPR and trends in EAPC for both sexes in 204 countries or territories. **A**, the ASPR per 100,000 in 2021. **B**, the EAPC of ASPR from 1992-2021. Panel A highlights the geographic distribution of ASPR, with regions in red showing higher prevalence rates and those in blue showing lower rates. Panel B displays the trends in ASPR, with red regions indicating an increasing trend in prevalence and blue regions showing a decreasing trend. This figure emphasizes the regional variability in disease prevalence and trends over time, highlighting areas of concern for public health interventions.

ASPR, age-standardized prevalence rate. EAPC, estimated annual percentage change.


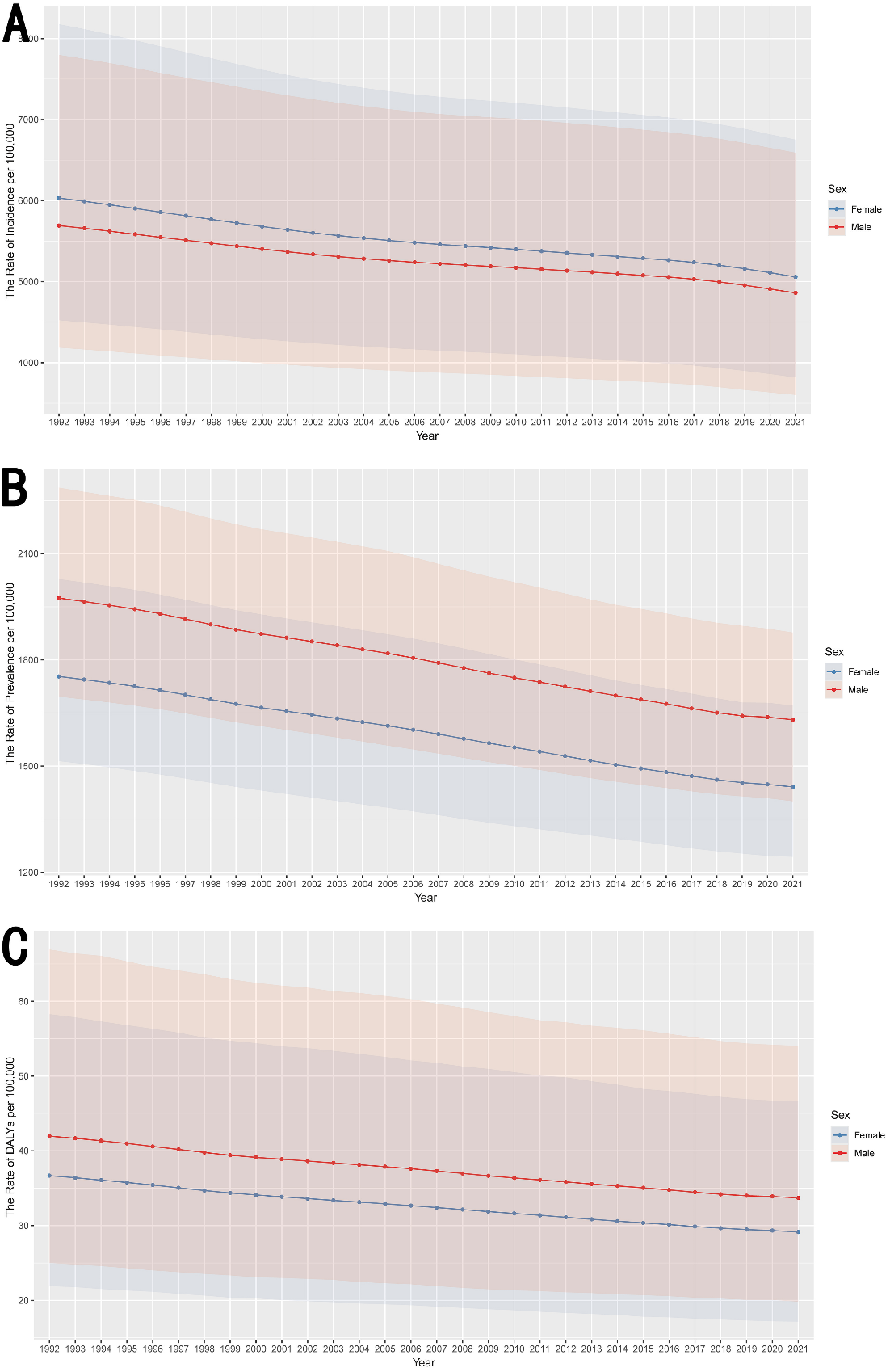


**Figure S2** Rates of otitis media on incidence, prevalence, and DALYs from 1992 to 2021. **A**, incidence. **B**, prevalence. **C**, DALYs. The figures indicate a consistent decline in the incidence, prevalence, and DALYs associated with otitis media, with males generally showing higher rates than females. The shaded areas represent the 95% confidence intervals.

DALYs, disability-adjusted life years.


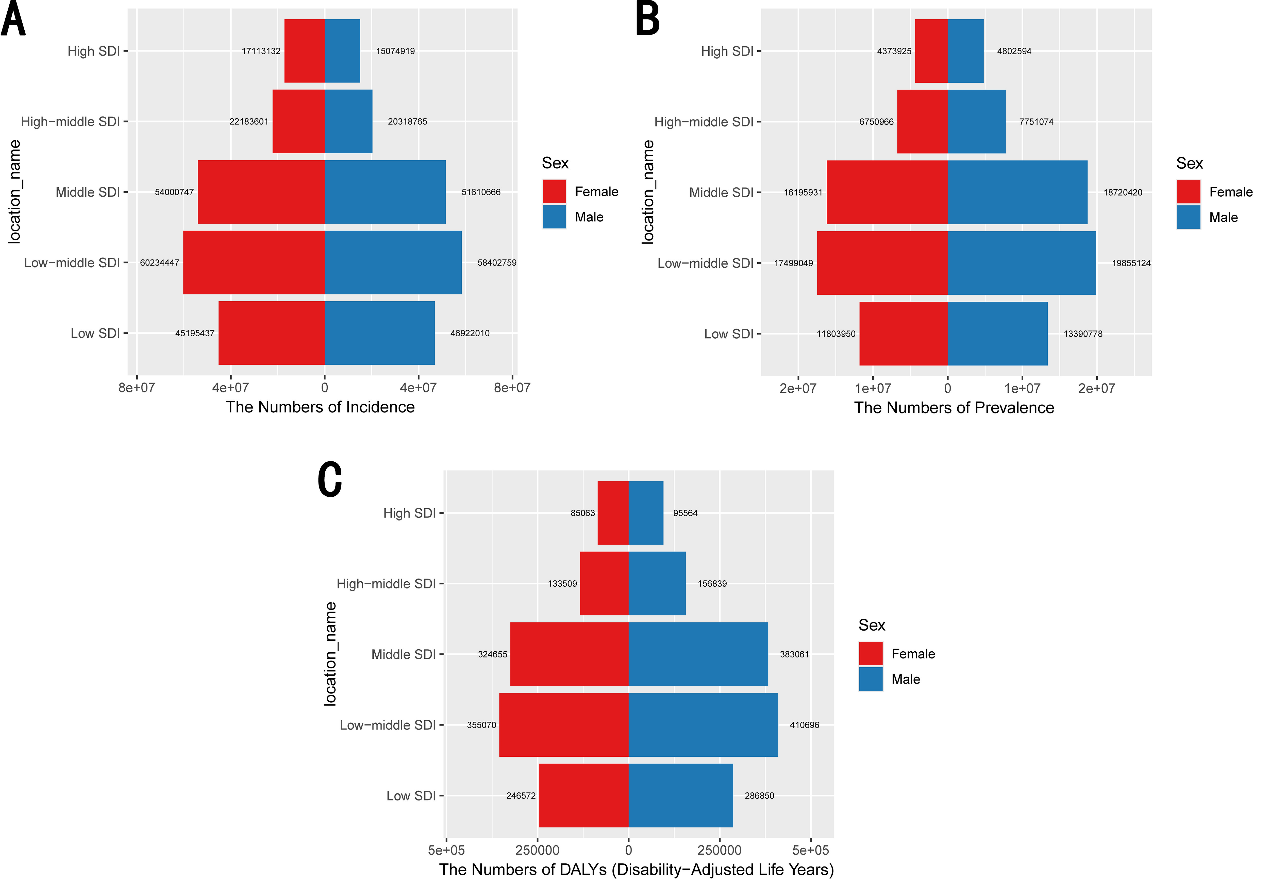


**Figure S3** Cases of otitis media on incidence, prevalence, and DALYs in different SDI. **A**, incidence. **B**, prevalence. **C**, DALYs. Panels A, B, and C show the numbers of incidence, prevalence, and DALYs across different age groups, revealing a higher burden among younger populations and in males. Panels D, E, and F present the burden by SDI levels, indicating that lower SDI regions have the highest burden of otitis media, particularly in terms of incidence and DALYs.

DALYs, disability-adjusted life years. SDI, socio-demographic index.


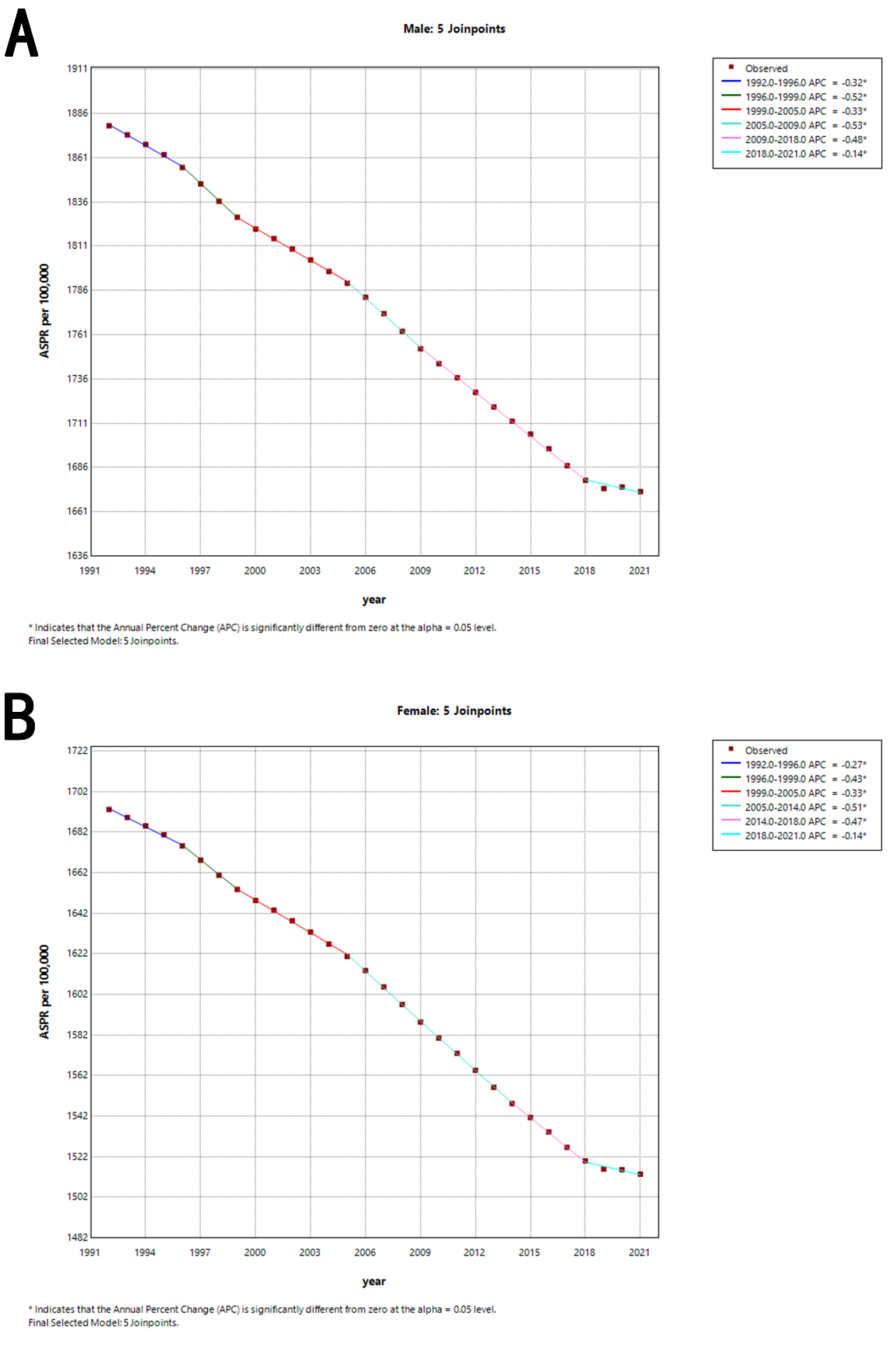


**Figure S4** Joinpoint regression analysis of the sex-specific ASPR for otitis media from 1992 to 2021. **A**, ASPR for males. **B**, ASPR for females. The graphs show periods of significant change in ASPR, identified through joinpoint regression analysis, with the APC values indicating the rate of change during each period.

ASPR, age-standardized prevalence rate. APC, annual percentage change.


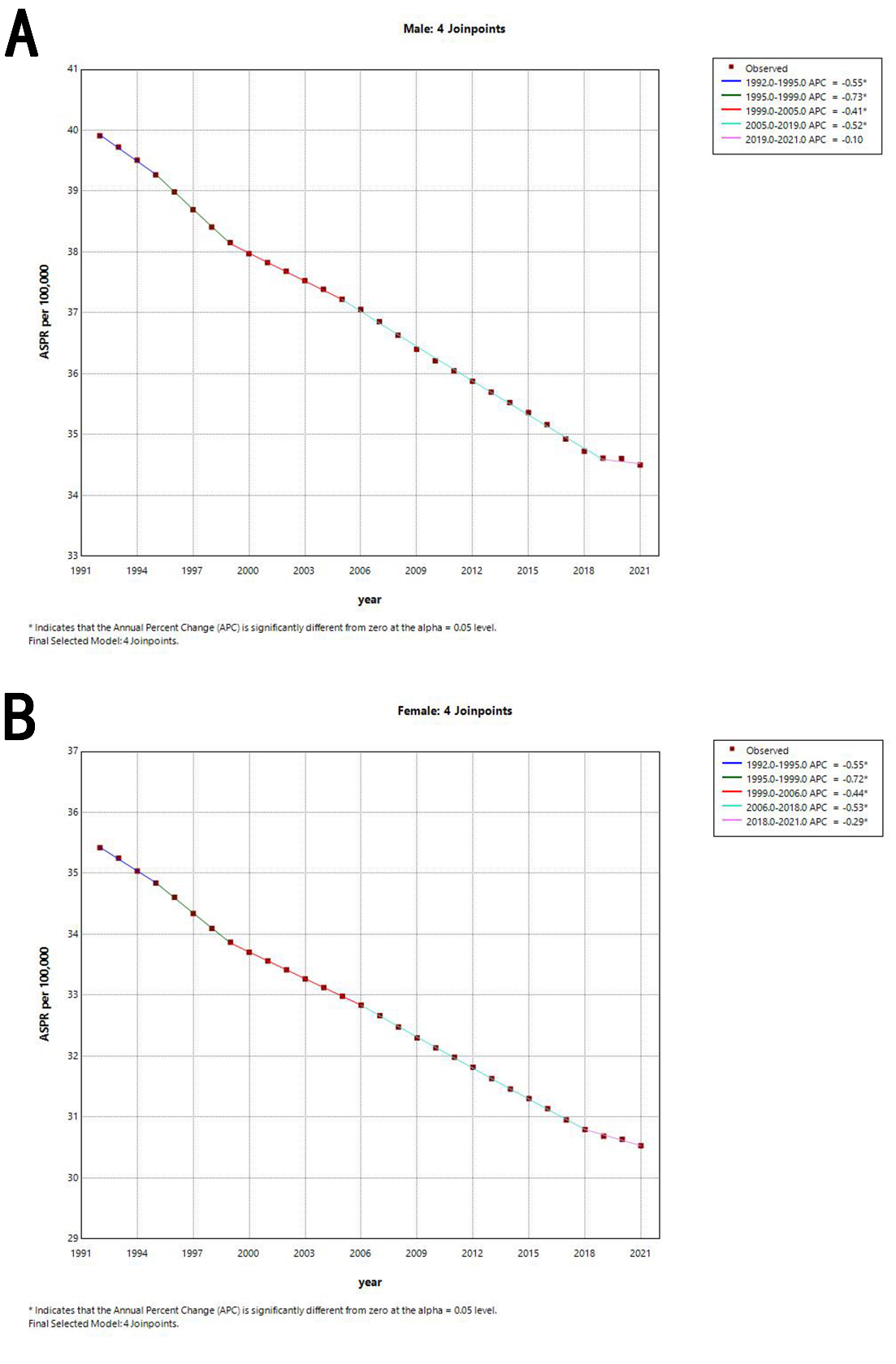


**Figure S5** Joinpoint regression analysis of the sex-specific ASDR for otitis media from 1992 to 2021. **A**, ASDR for males. **B**, ASDR for females. The graphs show periods of significant change in ASDR, identified through joinpoint regression analysis, with the APC values indicating the rate of change during each period.

ASDR, age-standardized DALY rate. APC, annual percentage change. DALY, disability-adjusted life year.


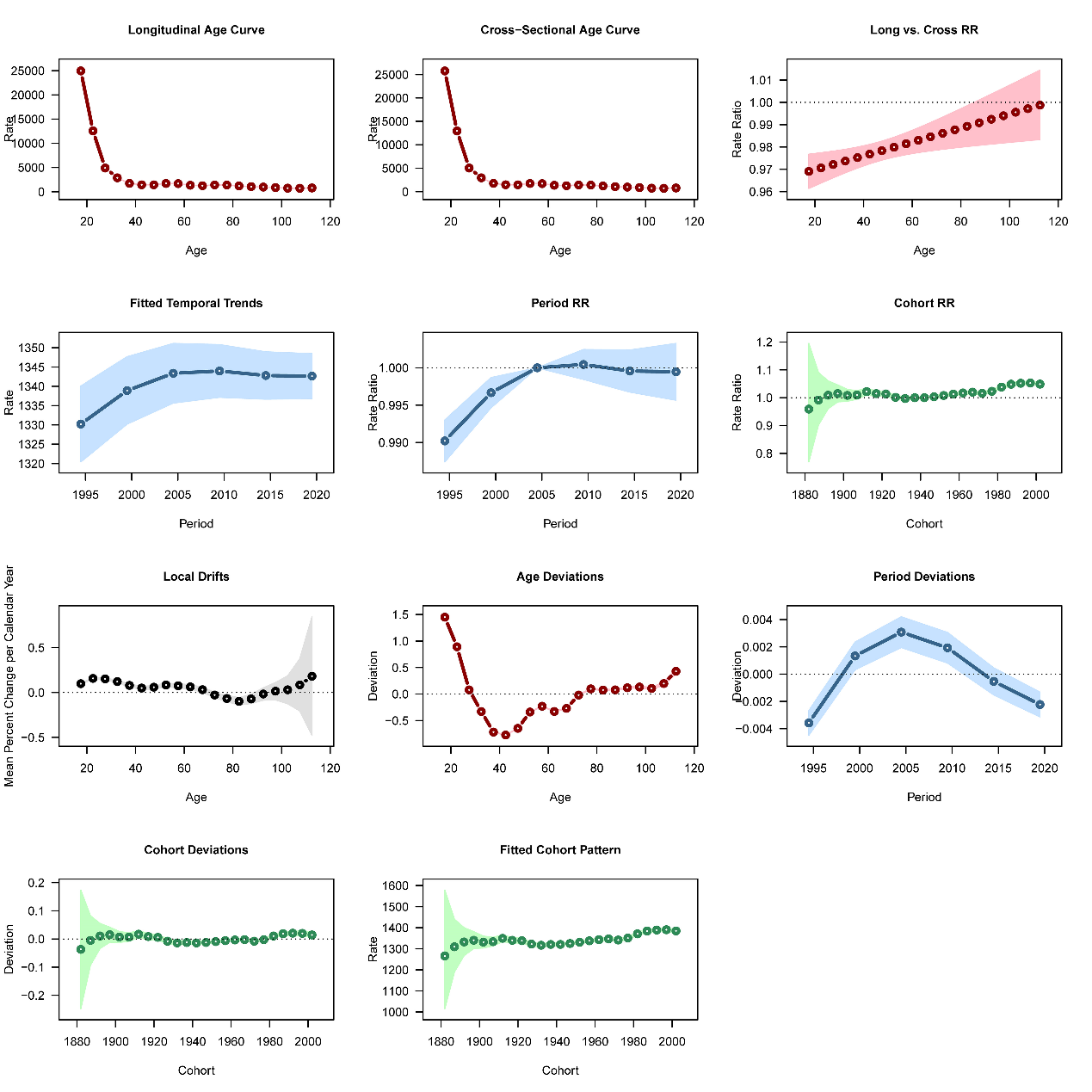


**Figure S6** Age, period and cohort effects on incidence of otitis media. The figure shows longitudinal and cross-sectional age curves, temporal trends, and relative risks associated with different periods and birth cohorts. The analysis reveals significant age effects, with the highest incidence in early childhood, and period effects suggesting a peak in risk around the mid-2010s. Cohort effects appear relatively stable, with little variation in risk across different birth cohorts. The deviation plots highlight the discrepancies between observed and expected trends, offering insights into the factors influencing otitis media incidence over time.


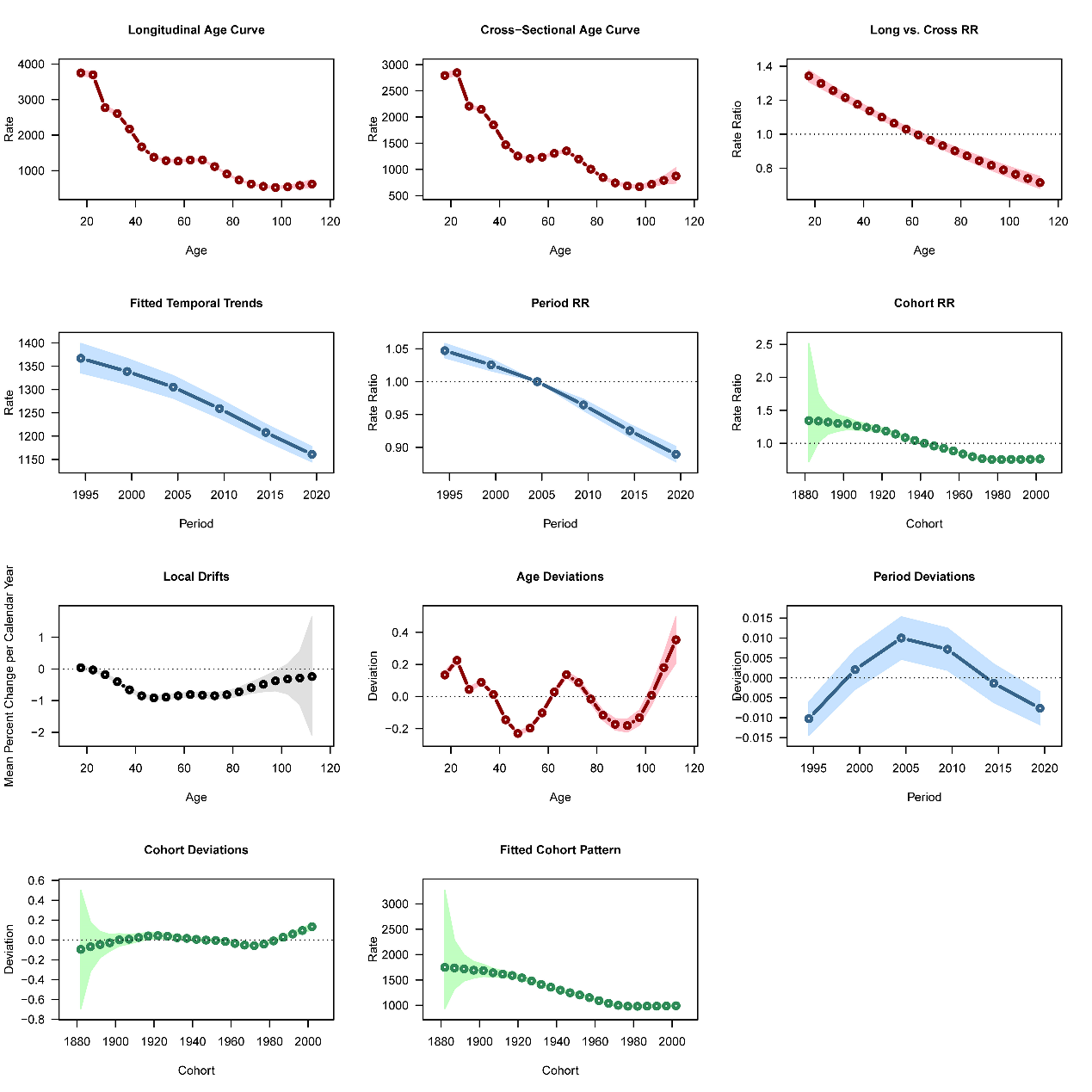


**Figure S7** Age, period and cohort effects on prevalence of otitis media. The figure includes longitudinal and cross-sectional age curves, temporal trends, and relative risks associated with different periods and birth cohorts. The analysis highlights significant age effects, with the highest prevalence in younger age groups, and period effects indicating a decline in prevalence risk over time. Cohort effects suggest a steady decrease in risk for more recent birth cohorts. The deviation plots illustrate differences between observed and expected prevalence trends, providing a deeper understanding of the factors influencing otitis media prevalence over time.


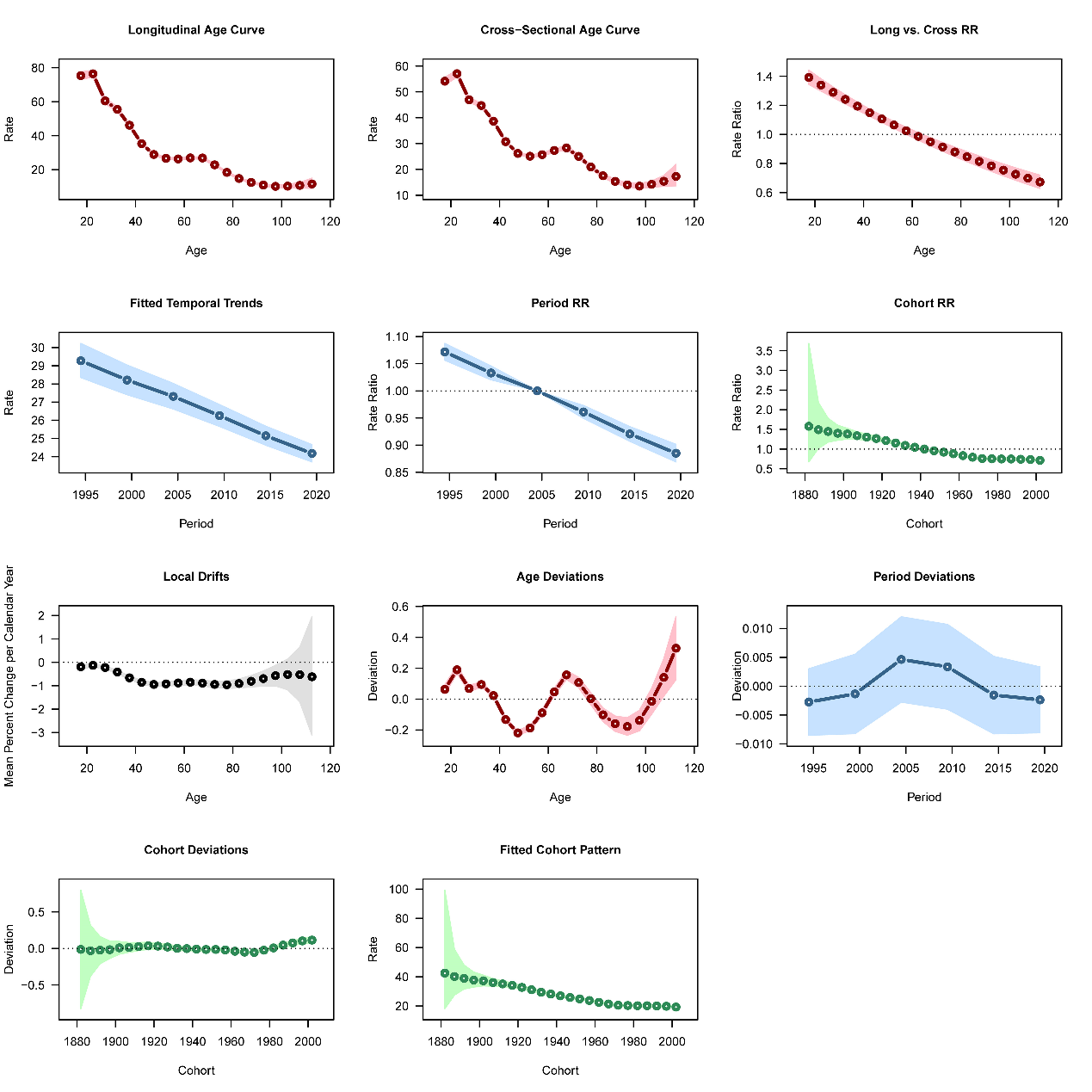


**Figure S8** Age, period, and birth cohort effects on DALYs of otitis media. The figure includes longitudinal and cross-sectional age curves, temporal trends, and relative risks associated with different periods and birth cohorts. The analysis shows significant age effects, with DALYs concentrated in younger age groups, and period effects indicating a steady decline in DALY risk over time. Cohort effects suggest a substantial reduction in risk for more recent birth cohorts, reflecting improvements in healthcare and prevention strategies. Deviation plots highlight small but noticeable shifts in disease burden over time, particularly around the turn of the century.

DALY, disability-adjusted life year.


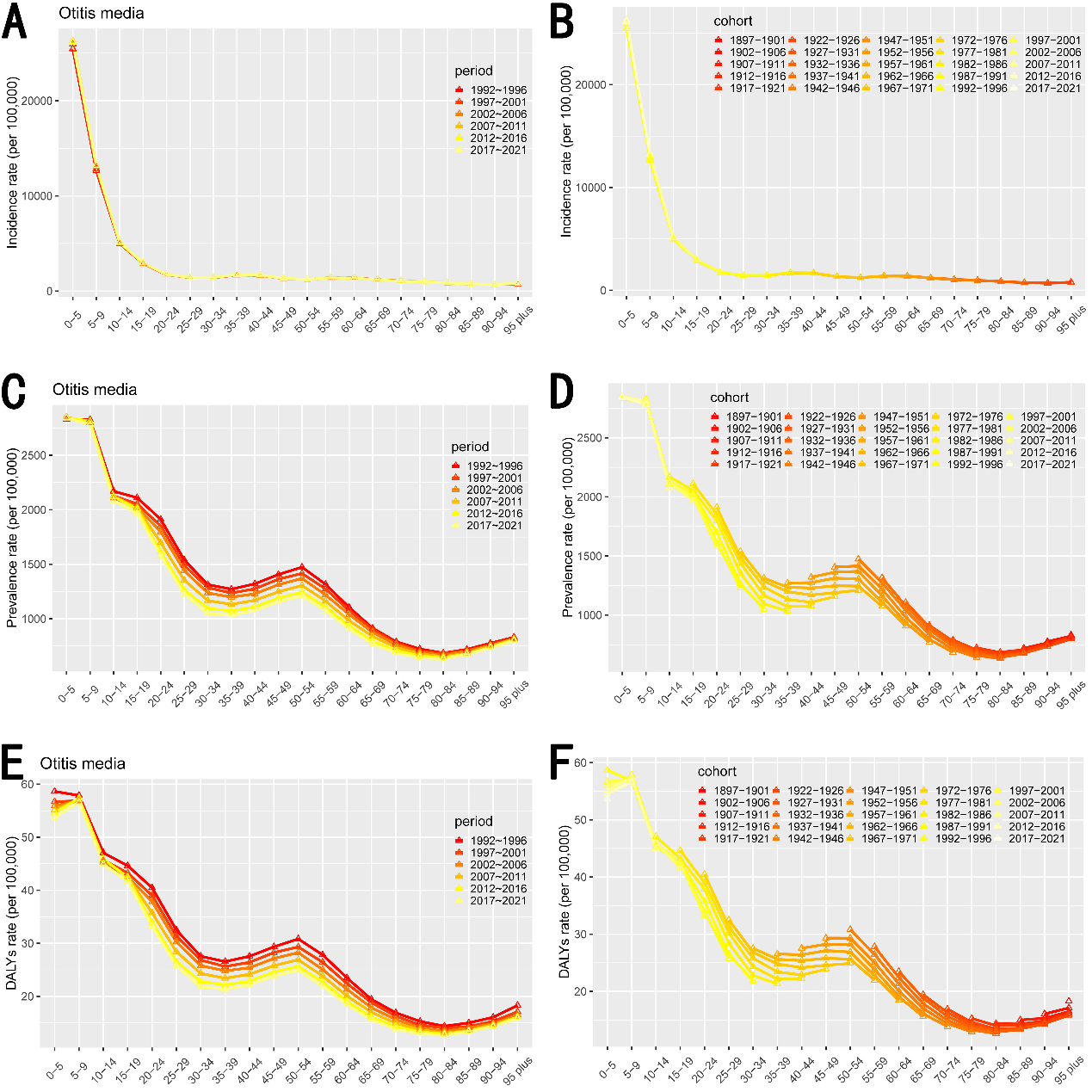


**Figure S9** Age-specific period and birth cohort effects on incidence, prevalence, and DALYs of otitis media. **A**, period effects on incidence. **B**, birth cohort effects on incidence. **C**, period effects on prevalence. **D**, birth cohort effects on prevalence. **E**, period effects on DALYs. **F**, birth cohort effects on DALYs. It reveals that the highest burden of otitis media is concentrated in the youngest age groups (<5 years), with a consistent decline in incidence, prevalence, and DALYs as age increases. There are slight variations across periods and cohorts, with more recent cohorts experiencing lower rates, particularly in older age groups. This figure underscores the importance of focusing on younger populations for prevention and intervention strategies.

DALY, disability-adjusted life year.


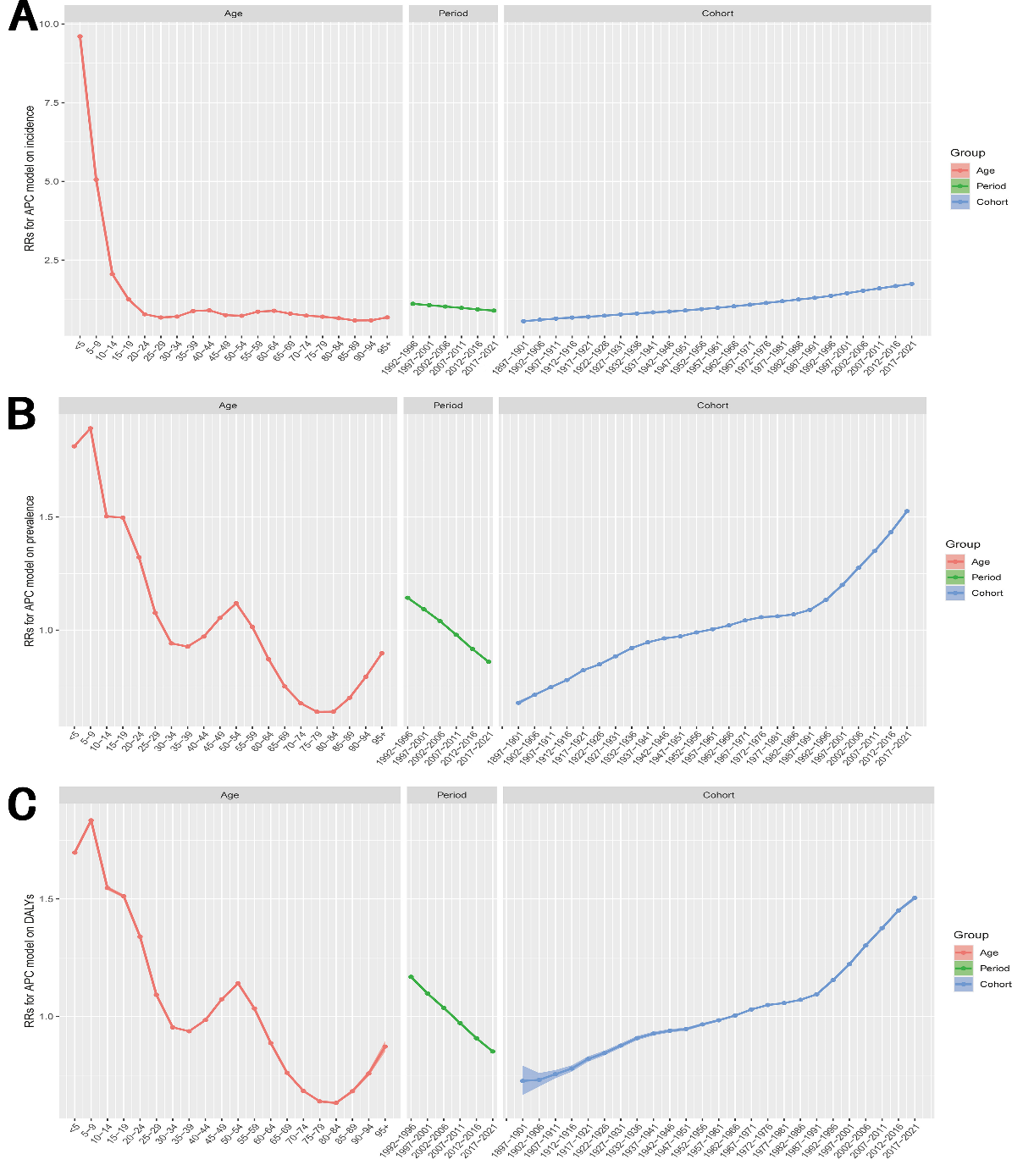


**Figure S10** The age, period, and birth cohort effects on the relative risks (RRs) for incidence, prevalence, and DALYs of otitis media. **A**, incidence. **B**, prevalence. **C**, DALYs. It reveals that the age effect shows a marked decrease in relative risk as age increases, particularly after early childhood. Period effects are relatively stable, with slight declines observed in prevalence and DALYs. Cohort effects indicate a substantial increase in relative risk for individuals born after 1940, suggesting a higher burden of otitis media in more recent cohorts. These trends highlight the importance of considering age, period, and cohort factors in understanding the epidemiology of otitis media and planning public health interventions.

DALY, disability-adjusted life year.


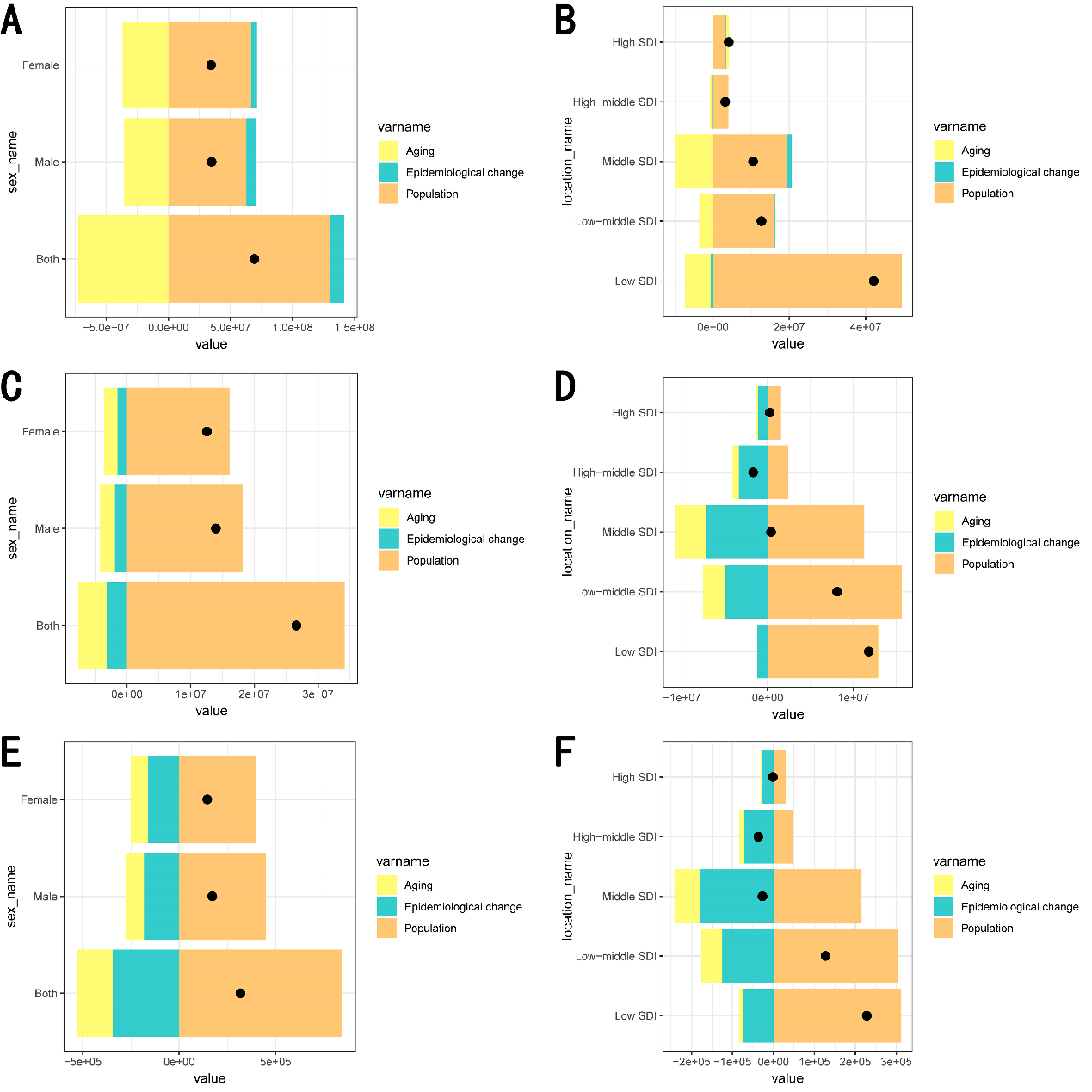


**Figure S11** Decomposition analysis of incidence, prevalence, and DALYs of otitis media. **A**, incidence stratified by sex. **B**, incidence stratified by SDI levels. **C**, prevalence stratified by sex. **D**, prevalence stratified by SDI levels. **E**, DALYs stratified by sex. **F**, DALYs stratified by SDI levels. Panels A and B highlight that population growth is the primary driver of increased incidence, especially in lower SDI regions. Panels C and D show a similar pattern for prevalence, with population growth again being the dominant factor. Panels E and F indicate that population growth significantly contributes to rising DALYs, particularly in low SDI areas, while aging and epidemiological changes play varying roles depending on sex and SDI.

DALY, disability-adjusted life year.


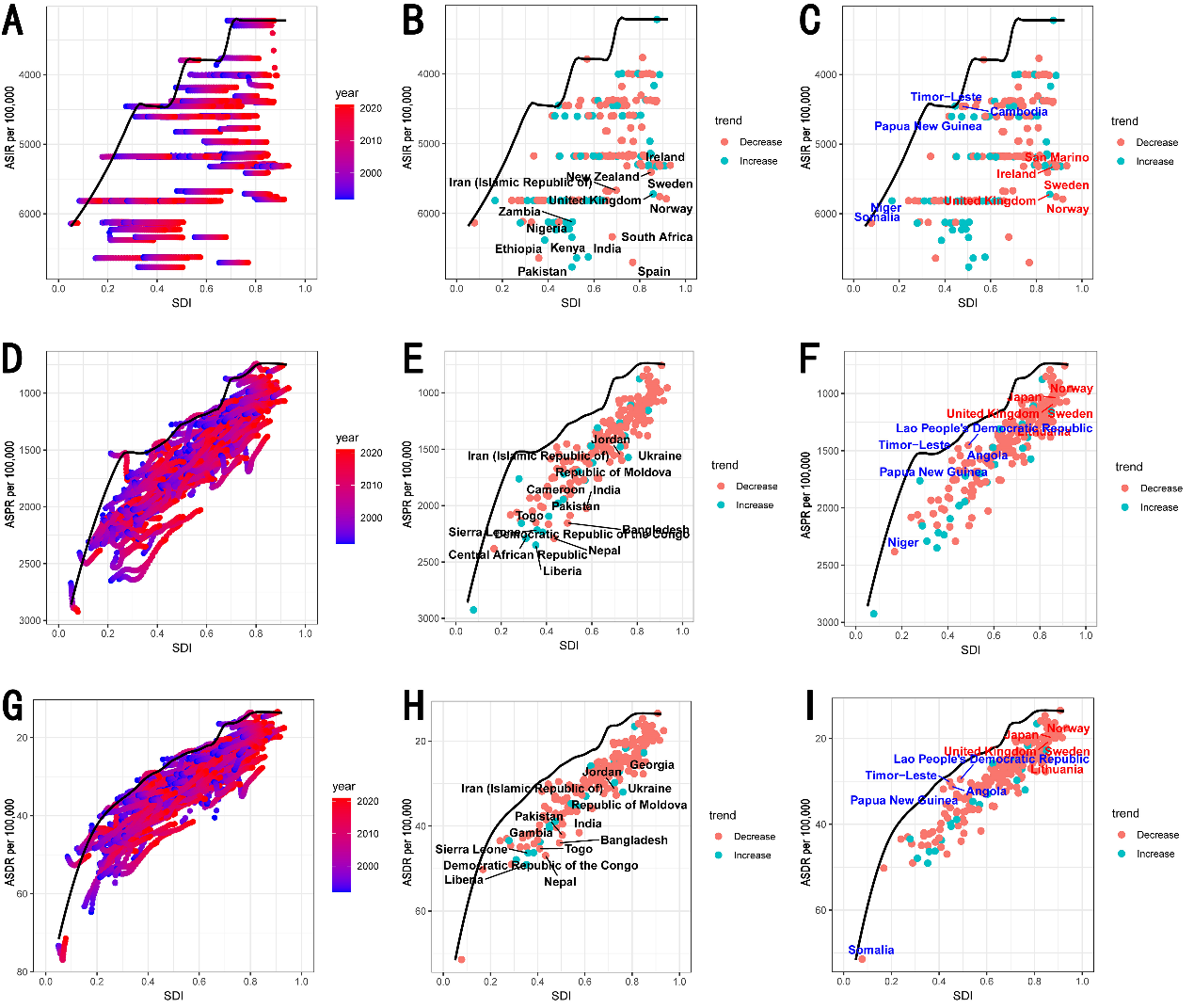


**Figure S12** Frontier analysis of ASIR, ASPR, and ASDR in 2021. **A, B, and C** Frontier analysis for ASIR. **D, E, and F** Frontier analysis for ASPR. **G, H, and I** Frontier analysis for ASDR. The color gradient from red to purple indicates the passage of time, while green and red dots indicate whether the trend is decreasing or increasing, respectively. Countries with low SDI generally show higher rates, but some have achieved significant improvements over time. Countries like Norway and Sweden show increasing ASIR despite high SDI, whereas countries like Niger show significant decreases. High-SDI countries like Japan maintain lower ASDR rates, while countries like Somalia show significant decreases despite lower SDI. Ireland and Papua New Guinea have diverging trends on ASIR despite similar SDI.

ASIR, age-standardized incidence rate. ASPR, age-standardized prevalence rate. ASDR, age-standardized DALY rate. DALY, disability-adjusted life year. SDI, socio-demographic index.


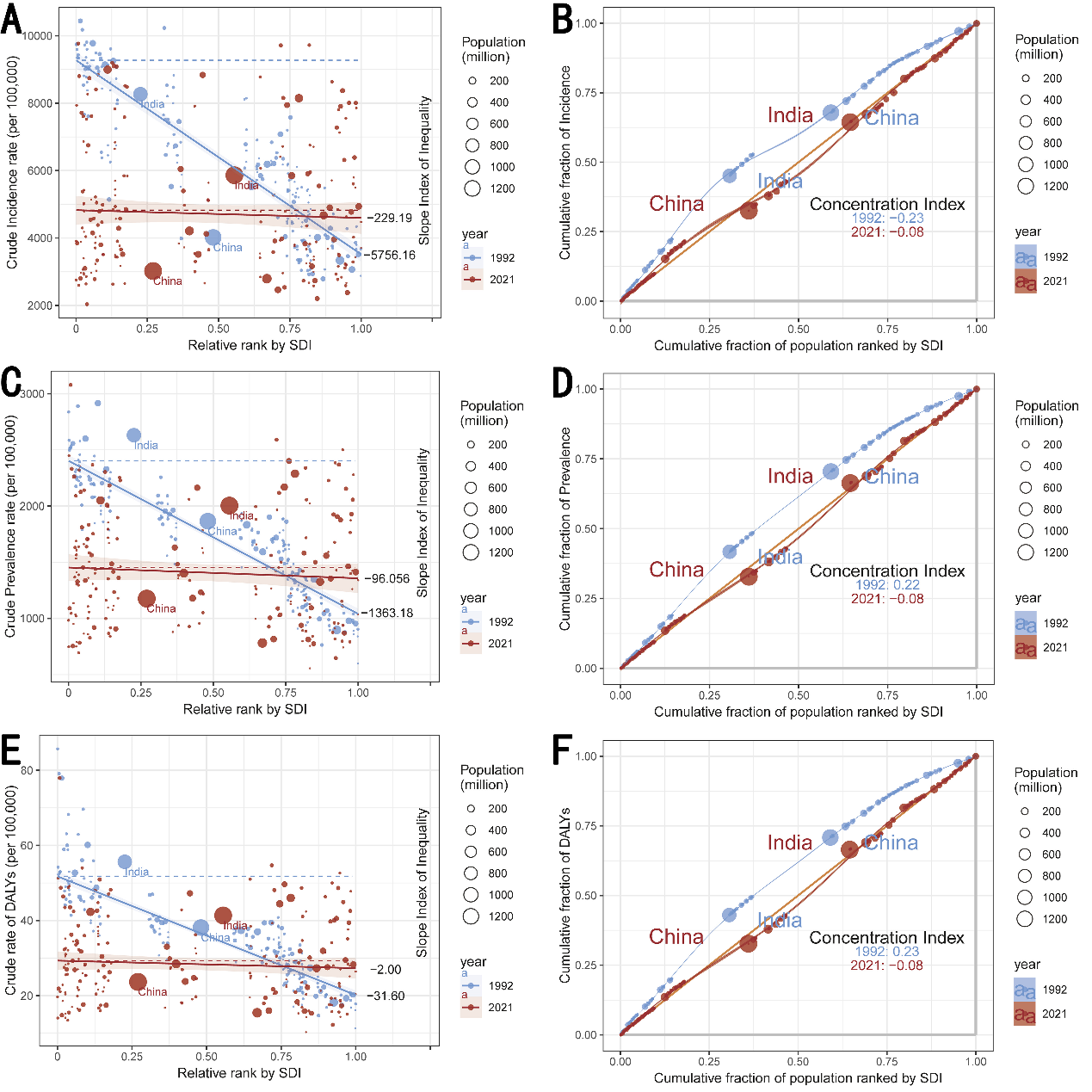


**Figure S13** Health inequality analysis of incidence, prevalence, and DALY rates from 1992 to 2021. **A,** slope index analysis of inequality on incidence. **B**, concentration index analysis on incidence. **C,** slope index analysis of inequality on prevalence. **D**, concentration index analysis on prevalence. **E,** slope index analysis of inequality on DALY rates. **F**, concentration index analysis on DALY rates. Notably, the 2021 data indicate that while the overall burden remains high, there have been marginal improvements in reducing inequality, as evidenced by the flatter slopes. The decreasing Concentration Index from 1992 to 2021 suggest that global efforts to address health inequality in otitis media have yielded some success, though targeted interventions are still necessary to further reduce these disparities, especially in low-SDI regions.

DALY, disability-adjusted life year. SDI, socio-demographic index.


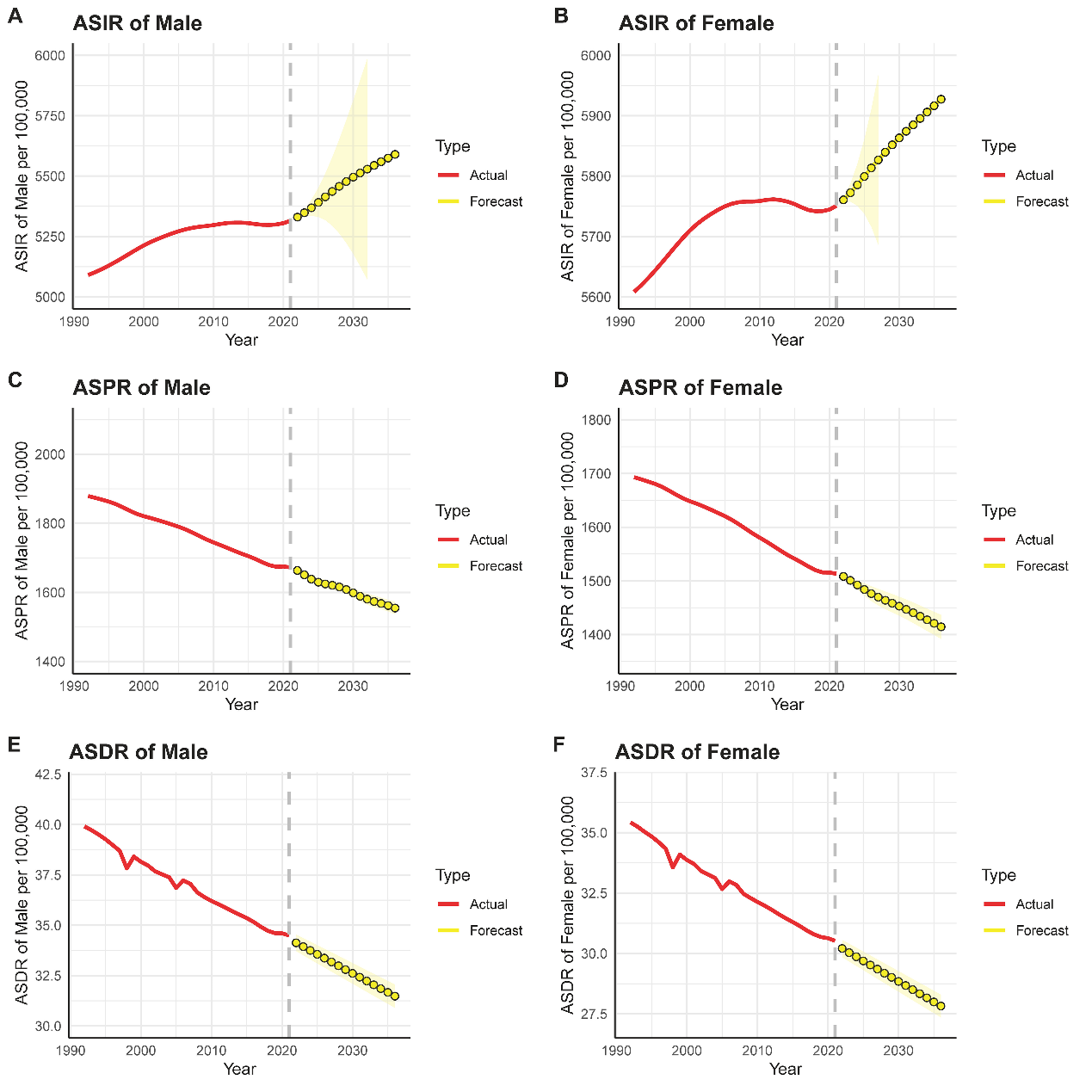


**Figure S14** Autoregressive integrated moving average (ARIMA) model predictions of ASIR, ASPR, and ASDR for males and females. **A**, ASIR for males. **B**, ASIR for females. **C**, ASPR for males. **D**, ASPR for females. **E**, ASDR for males. **F**, ASDR for females. It highlights a projected increase in ASIR and a decrease in ASPR and ASDR for both sexes, with varying degrees of confidence in the predictions.

ASIR, age-standardized incidence rate. ASPR, age-standardized prevalence rate. ASDR, age-standardized DALY rate. DALY, disability-adjusted life year.


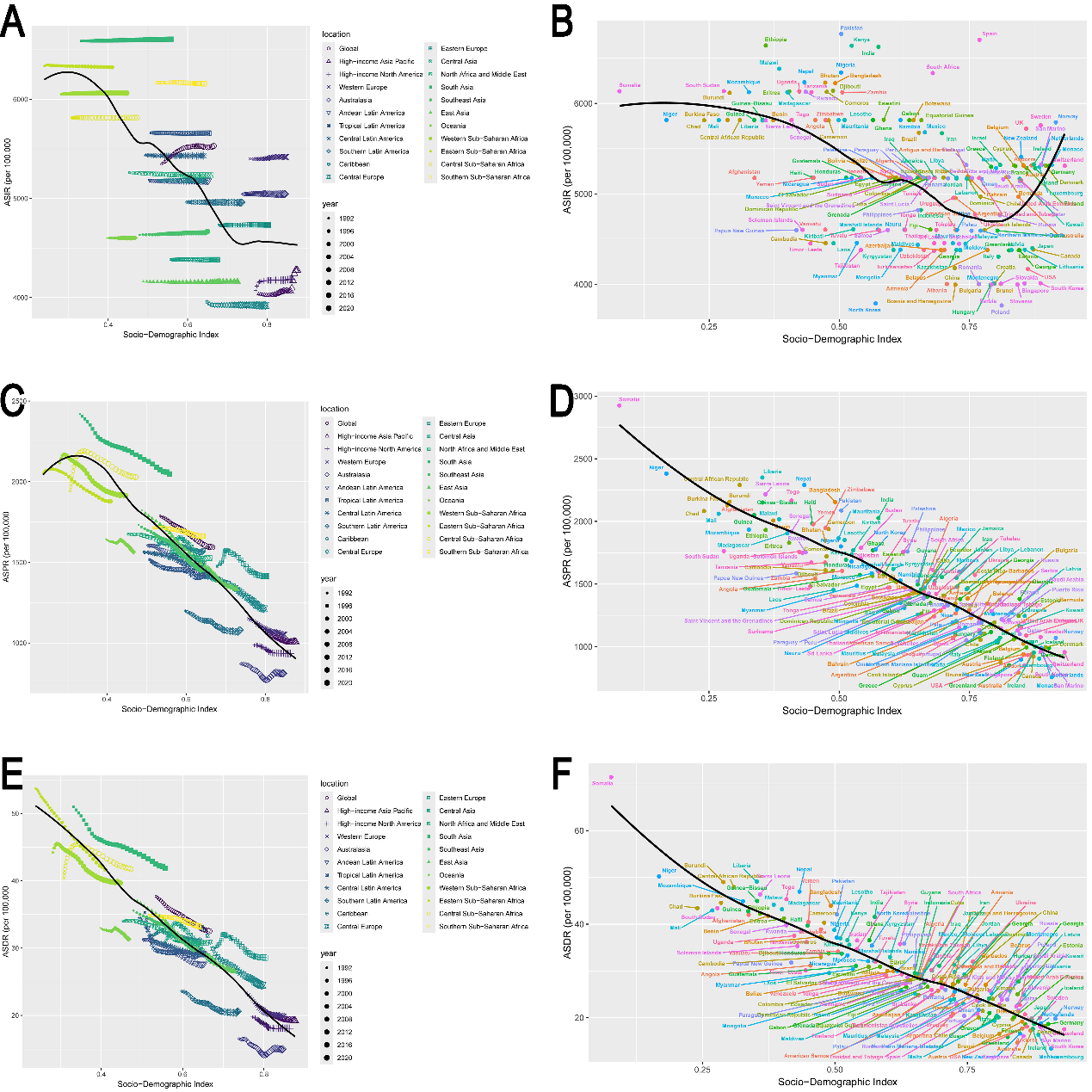


**Figure S15** The correlation between SDI and global burden of otitis media. **A, C,** and **E** The change trends and correlation of ASIR, ASPR, ASDR and SDI from 1992 to 2021 in 21 regions. **B, D,** and **F** The correlation between ASIR, ASPR, ASDR and SDI of 2021 in 204 countries or territories. It suggested that 21 regions or 204 countries (or territories) with higher SDI are associated with lower prevalence rates of ASIR, ASPR, and ASDR.

ASIR, age-standardized incidence rate. ASPR, age-standardized prevalence rate. ASDR, age-standardized DALY rate. DALY, disability-adjusted life year. SDI, socio-demographic index.
